# Distributed and dynamical communication: a mechanism for flexible cortico-cortical interactions and its functional roles in visual attention

**DOI:** 10.1038/s42003-024-06228-z

**Published:** 2024-05-08

**Authors:** Shencong Ni, Brendan Harris, Pulin Gong

**Affiliations:** School of Physics, University of Sydney, Sydney, NSW Australia

**Keywords:** Network models, Attention

## Abstract

Perceptual and cognitive processing relies on flexible communication among cortical areas; however, the underlying neural mechanism remains unclear. Here we report a mechanism based on the realistic spatiotemporal dynamics of propagating wave patterns in neural population activity. Using a biophysically plausible, multiarea spiking neural circuit model, we demonstrate that these wave patterns, characterized by their rich and complex dynamics, can account for a wide variety of empirically observed neural processes. The coordinated interactions of these wave patterns give rise to distributed and dynamic communication (DDC) that enables flexible and rapid routing of neural activity across cortical areas. We elucidate how DDC unifies the previously proposed oscillation synchronization-based and subspace-based views of interareal communication, offering experimentally testable predictions that we validate through the analysis of Allen Institute Neuropixels data. Furthermore, we demonstrate that DDC can be effectively modulated during attention tasks through the interplay of neuromodulators and cortical feedback loops. This modulation process explains many neural effects of attention, underscoring the fundamental functional role of DDC in cognition.

## Abstract (web summary)

Flexible interareal communication mechanism based on realistic spatiotemporal dynamics of propagating waves of neural population activity, showing how modulating this communication explains various neural effects of attention.

## Introduction

Brain functions ranging from perception to cognition and behavior fundamentally depend on flexible communication between cortical areas^1–3^. Understanding the mechanisms underlying flexible interareal communication is thus of central importance to systems and computational neuroscience. One prevalent view suggests that cortical areas interact through a low-dimensional subspace^4^ within which population activity in one cortical area is functionally related to that in another area. This subspace-based communication has been observed in a variety of cortical areas^4–6^. However, the circuit mechanisms underlying the emergence of cortical communication subspaces and their ability to rapidly reconfigure to facilitate flexible interareal communication remain unclear.

Another view proposes that interareal synchrony or coherence of gamma oscillations can coordinate interactions of neural assemblies to route information between different brain areas^2^. Empirical evidence has consistently demonstrated that enhanced gamma-frequency communication is critical for routing task-relevant neural messages during cognitive processes, such as attention^7,8^, memory^9^, and learning^10^. Classical studies suggest that gamma oscillations are regular and sustained phenomena, a notion that holds true only when experimental measurements are averaged over many trials. However, recent studies evaluating data at the level of single trials have increasingly demonstrated that gamma activity occurs intermittently as variable bursts^11–13^, exhibiting large fluctuations. While the bursting properties of gamma activity might be beneficial for interareal interactions^14^, previous studies have mainly focused on the temporal characteristics of gamma activity. Yet, a growing body of evidence indicates that gamma activity, and brain activity in general, unfolds not only in time but also in space, exhibiting rich and complex spatiotemporal wave-like activity patterns^15,16^, which potentially enable a powerful mechanism for facilitating flexible interareal communication. Furthermore, the fundamental question of whether and how the two prevalent views — the communication subspace and oscillation synchrony — can be unified to yield a more comprehensive and fundamental understanding of cortico-cortical communication remains unresolved.

Here, we propose a mechanism based on the coordinated interactions of rich and complex spatiotemporal dynamics of propagating wave patterns, enabling the implementation of interareal communication in a fundamentally distributed and dynamic manner. We illustrate this mechanism, referred to as distributed and dynamical communication (DDC), by developing a canonical large-scale cortical circuit model involving two cortical areas connected through bottom-up (feedforward) and top-down (feedback) connections, forming cortico-cortical loops. Our findings demonstrate that localized wave patterns emerging from this large-scale circuit can capture and explain a wide variety of neural dynamics observed at the individual neuron and circuit levels. These dynamics include correlated neural variability, gamma bursts nested within theta oscillations, as well as complex propagating dynamics (specifically, anomalous superdiffusion) of neural activity patterns.

We demonstrate that the coordinated interactions of these wave patterns within the cortical areas, on one hand, account for the emergence of transient synchronization of gamma bursts, which plays a crucial role in facilitating interareal communication. On the other hand, these interactions explain the formation and reconfiguration of communication subspaces, providing a mechanism for flexible switching between different subspaces, as observed in ref. ^17^. By exploiting the rich spatiotemporal dynamics of the cortical circuits, rather than relying solely on regular and sustained oscillatory activities as in conventional studies, our DDC mechanism effectively integrates the perspectives of communication subspaces and oscillation synchrony, providing a unified account of interareal communication. Importantly, we illustrate that the DDC mechanism offers crucial functional advantages, such as the ability to flexibly and rapidly route neural responses to multiple stimuli across cortical areas. Through the analysis of Neuropixels data from the Allen Institute^18^, we further validate the key predictions of the DDC mechanism, including the interrelations between gamma burst synchrony, theta-gamma coupling, and subspace-based interareal communications.

Furthermore, we elucidate the essential role of DDC in brain functions, particularly in cued top-down attention. Specifically, we demonstrate that DDC can be effectively modulated during cued attention tasks through the interplay between neuromodulators (e.g., acetylcholine) and cortico-cortical loops. This modulation process provides a unified account of a range of neural effects of attention that otherwise remain disjointed in previous studies. These effects include the classical observation of biased competition^19,20^, local modulation of cortical states^21^, reductions in neural variability^22^ and spike-count correlations^23,24^, increased gamma synchrony between sensory and association areas (e.g., V4 and frontal eye field)^8^, as well as increased theta-gamma coupling triggered by attention cues^25^. These results prompt a reconceptualization of cued attention as an emergent property arising from the orchestrated modulation of DDC across distinct cortical areas. This perspective offers a DDC-based framework for understanding how attention, a central brain function, is implemented in large-scale neural circuits.

## Results

### A spiking neural circuit model with interconnected cortical areas

**Fig. 1:**
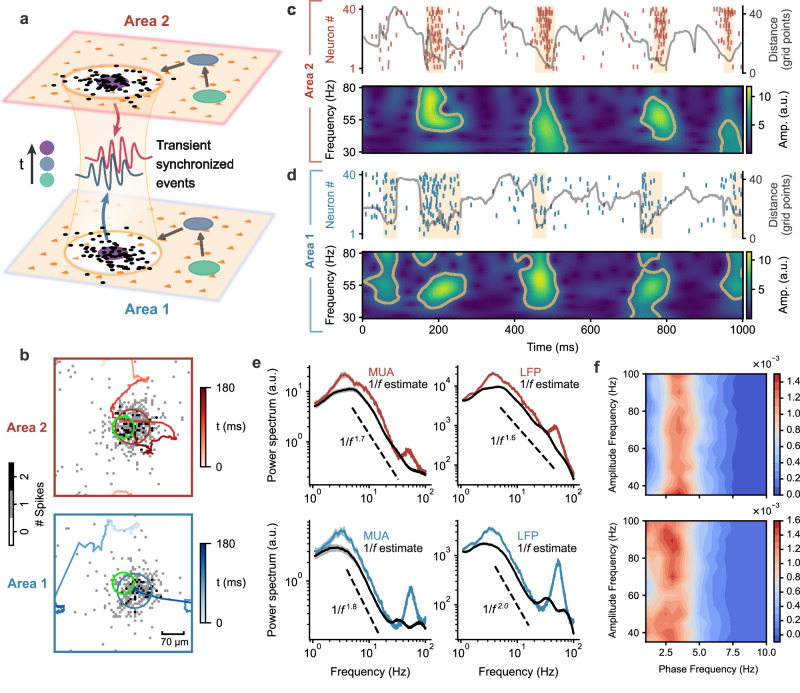
Spatiotemporal dynamics of localized wave patterns emerging in the two-area neural circuit can explain neural dynamics at the individual-neuron and population levels. **a** Schematic of the circuit model with two interconnected areas. Each network consists of excitatory (orange triangles) and inhibitory (orange circles) neurons. Spiking activity patterns (black dots) in both areas exhibit intermittent synchronization at different topographically aligned regions (big circles, color-coded over time). During synchronized events, local population neural activities, particularly in the gamma band, exhibit transient phase locking between areas (blue and red curves). **b** Snapshot of localized spiking activity patterns (marked by blue and red circles) in area 1 (bottom) and area 2 (top). Excitatory neuron spike counts over a 10 ms period are indicated by gray dots. Trajectories of the center of mass of patterns over the preceding 180 ms are shown as lines, with colors representing time. The green circle indicates the region of neurons sampled for analyses in **c**–**e**. **c** Top: Raster plot of spiking activity (vertical lines) of a local group of excitatory neurons at the center of area 2 (green circle in **b**, only 40 out of 80 neurons' spikes are shown), showing transitions between On (marked by yellow epochs) and Off states. The gray line shows the distance between the center of mass of the localized spiking activity pattern and the center of area 2. Bottom: Wavelet time-frequency spectrogram of the MUA at the center of area 2 during the same period as the raster plot, showing gamma bursts (circled by yellow lines) aligned with On states. **d** Same as (**c**) but for area 1. **e** Average power spectrum of MUA and LFP at the centers of area 1 (bottom row) and area 2 (top row). Two distinct peaks appear in the theta and gamma bands. The black lines show the separated arrhythmic 1/*f* components, with the exponents indicated by the dashed lines. The shaded regions around the lines represent ± 1 SEM (*n* = 30 random network realizations). **f** Average comodulograms across 30 network realizations quantifying theta phase-gamma amplitude coupling in the LFP of areas 1 (bottom) and 2 (top). Source data can be found in Supplementary Data 1.

We develop a canonical neural circuit model comprising a lower area (area 1) and a higher area (area 2) positioned along the hierarchical organization of the cortex (Fig. 1a). The model incorporates several well-established properties of the cortex, including distance-dependent coupling^26^, balanced excitation and inhibition^27,28^, and neural firing adaptation^29^. In addition, our model incorporates the heterogeneity of local circuits across cortical areas, with excitatory synaptic strengths increasing from sensory to association areas^30^. The two areas in the model are coupled through feedforward and feedback connections, thus interacting with each other through cortico-cortical loops. Our model serves as a mechanistic framework for understanding the flexible communication between any interconnected brain regions operating at different levels within the cortical structural hierarchy. For instance, it allows us to quantitatively characterize the interactions between a sensory area (V4) and an association area (frontal eye field) during top-down attention tasks, as illustrated below. Both cortical areas are close to the transition state between different cortical states, as we have previously identified^31^; we refer to this transition state as the dynamical working regime of our model.

Within the dynamical working regime, individual neurons in both areas fire sparsely and irregularly during spontaneous activity; the firing rates of neurons in area 1 and area 2 are 6.06 ± 0.01 and 7.39 ± 0.03 Hz (mean ± SEM, *n* = 60 networks), respectively. The coefficient of variation (CV) of interspike intervals and the Fano factor (the ratio of the variance of spike counts to mean spike counts) of these neurons display broad distributions, with mean values exceeding 1 (CV in area 1: 1.114 ± 0.004, CV in area 2: 1.193 ± 0.005; Fano factor in area 1: 1.315 ± 0.008, Fano factor in area 2: 1.764 ± 0.012, mean ± SEM, *p* < 10^−30^ for all mean values compared to 1, two-sided one-sample t-test, *n* = 60 networks, see “Methods” section); these values are quantitatively comparable to those measured experimentally^32^. Note that neural variability in the higher area (i.e., area 2) is significantly greater than that in the lower area (i.e., area 1) (*p* < 10^−21^ for CV and *p* < 10^−42^ for Fano factor, two-sided paired t-test, *n* = 60 networks). This heterogeneity of neural dynamics across cortical regions is consistent with the observations of increasing neural variability along the cortical hierarchy, as reported in ref. ^33^.

We find that inactivating distant sources of either feedforward or feedback input to area 2 and area 1 leads to a significant decrease in the spiking variability of their neurons. Specifically, disconnecting the top-down connection results in a significant decrease in the Fano factor in area 1 to 1.227 ± 0.007 (mean ± SEM, *p* < 10^−34^, two-sided paired t-test, *n* = 60 networks). Similarly, disconnecting the bottom-up connection leads to a reduction in the Fano factor in area 2 to 1.559 ± 0.010 (mean ± SEM, *p* < 10^−41^, two-sided paired t-test, *n* = 60 networks). This is consistent with previous experimental studies that have reported reduced spiking variability upon inactivation of either feedforward or feedback input to cortical visual areas in alert primates^34^. These results indicate that the cortico-cortical loops in our model are as effective as those observed in experimental studies, thus providing a quantitative modeling framework for revealing the mechanisms of cortico-cortical interactions and communication.

### Propagating wave patterns emerging from the neural circuit capture a wide range of realistic neural dynamics

Despite the variable spikes of individual neurons, coherent localized activity patterns (wave packets) emerge at the circuit level within the dynamical working regime. Our previous study has demonstrated that these patterns exhibit rich and complex dynamics, serving as a spatiotemporal mechanism for bottom-up stimulus-driven visual attention^35^. This mechanism explains a variety of neural features of visual bottom-up attention, including superdiffusive Lévy motion and theta oscillations, which facilitate flexible switching between exploitation and exploration^35^, a hallmark feature of flexible attention sampling. Building upon these findings, our current study explores the fundamental role of the rich spatiotemporal dynamics of wave patterns in enabling flexible communication between interconnected cortical areas along the cortical hierarchy.

Figure 1b shows a coherent, localized spiking pattern emerging in area 1 and 2 circuits, respectively. This localized pattern hovers around one location for a while and then switches to another location, exhibiting clusters of small movement step-sizes that are intermittently interspersed by long jumps (Supplementary Fig. 1 and Supplementary Movie 1). This intermittent propagation of the localized activity patterns can be characterized as superdiffusive Lévy motion (Supplementary Fig. 2a), as in our previous study^35^. Notably, superdiffusive Lévy motion has been demonstrated to underlie the propagation of neural population activity in the MT area of monkeys^36^ as well as in the hippocampus of mice^37,38^. We first illustrate that the propagating wave patterns capture a range of realistic neural dynamics. By tracking the center of mass of these wave patterns (see Methods), we find that when a pattern dwells around one location, spiking activity at the corresponding location exhibits vigorous ensemble, which then return to a relatively quiescent state after the pattern moves away (top rows in Fig. 1c and d); consequently, neurons fluctuate between phases of vigorous (On state) and faint (Off state), resulting in bursting activity. Note that it has been shown that such On-Off transitions in spontaneous activity are a general feature across multiple brain regions of behaving monkeys^21,39^.

To characterize these bursts, we detect them by thresholding the instantaneous multiunit activity (MUA), which represents the average firing rate of a local group of excitatory neurons in a circular region with a radius of 5 grid points positioned at the network center (indicated by the green circle in Fig. 1b, see Methods for burst detection details, which are consistently applied throughout this study). Given that the bursts arise from localized wave patterns, the radius of this region for MUA definition is chosen according to the spatial scale of these patterns (~8 grid points); any radius close to the patterns’ spatial scale would yield similar burst detection results. Our analysis reveals that in area 1, the duration of the On state of bursts is *t*_on_ = 54.46 ± 1.98 ms (mean ± SEM, *n* = 30 networks), while the Off state has a duration of *t*_off_ = 280.44 ± 13.89 ms (distributions are shown in Supplementary Fig. 3a); these durations are quantitatively comparable to those measured in the spontaneous activity of macaques V4^21^, with *t*_on_ = 97 ± 36 ms and *t*_off_ = 118 ± 47 ms (mean ± SD). In the area 2 circuit, we find that the dynamical patterns can similarly explain the burst-like, On-Off transitions of spiking activity, with *t*_on_ = 43.60 ± 0.66 ms and *t*_off_ = 302.62 ± 14.53 ms (distributions are shown in Supplementary Fig. 3a).

In both areas, the bursts of spiking arising from the local pattern dynamics are associated with gamma bursts in either local field potential (LFP; which is calculated based on the sum of incoming synaptic currents of excitatory neurons; see “Methods” section) or MUA. To demonstrate this association, we perform wavelet transform-based time-frequency analysis on LFP and MUA (see “Methods” section), we find that during the transient epochs of spiking bursts, there exist gamma bursts in both LFP and MUA (bottom rows in Fig. 1c and d, only the spectrograms for MUA are shown). Our further statistical analysis of the duration and power of these gamma bursts indicates that they exhibit large fluctuations, as measured in experimental studies (Supplementary Fig. 4). On average, these gamma bursts would give rise to a gamma peak in the power spectrum of MUA and LFP in both areas (Fig. 1e). As in other spiking neural circuits, in our model the gamma activity emerges via the pyramidal-interneuron gamma (PING) mechanism.

The spiking bursts (i.e., On states) caused by the dynamical wave patterns in both areas occur ≈3 times per second (Fig. 1c and d), indicating the presence of theta oscillations^35^. This is supported by the power spectrum analysis of both LFP and MUA, which exhibits a theta peak sitting on top of 1/*f* activity; the 1/*f* component can be separated from the oscillatory activity by using the irregular resampling method developed in ref. ^40^ (Fig. 1e). In our model, theta oscillations arise from the mechanism of spike frequency adaptation (SFA), leading to the formation of an oscillatory activity pattern known as a ‘breather’, as described in dynamical systems theory.

To gain a theoretical understanding of this mechanism, we use a firing rate model that incorporates the firing rate adaptation mechanism and the overall coupling structure of the spiking neural circuit model. Through a dynamical stability analysis for the stationary localized activity pattern in response to perturbations (see Supplementary Methods 1), it is shown that the onset of instability of this pattern leads to the onset of localized oscillatory pattern (breather). The analytically obtained oscillatory frequency at the onset of instability (*f*_*c*_) increases with the firing rate adaptation strength, with ${f}_{c}=\scriptstyle\sqrt{\left[k-(\tau /{\tau }_{{{{{{{{\rm{A}}}}}}}}})\right]/(\tau {\tau }_{{{{{{{{\rm{A}}}}}}}}})}/(2\pi )$, where *k* is the adaptation strength, *τ* is the decay time constant of firing rate, and *τ*_A_ is the decay time constant of adaptation^41^ (Supplementary Fig. 5b). We further validate this prediction in our full spiking neural circuit model; as shown in Supplementary Fig. 6, the frequency of theta activity in the circuit model increases as SFA increases, indicating that neural adaptation is the mechanism underlying the formation and modulation of theta oscillations.

As illustrated above, the On-Off state alternation in the slow time scale results in theta oscillations while the neuronal fluctuation in the fast time scale within the On state gives rise to the gamma bursts^35^. This suggests a coupling between the theta and gamma activities. To quantify such theta-gamma coupling in our model, we calculate the phase-amplitude coupling modulation index^42^ for LFP in both areas (see Methods); this index measures the intensity of the modulation of the amplitude of oscillations at one frequency band by the phase of oscillations at another frequency band. As shown in the theta phase - gamma amplitude comodulograms (Fig. 1f), the phase-amplitude coupling is strongest between the amplitude of ≈70 Hz oscillation and the phase of ≈3 Hz oscillation, indicating the gamma amplitude is modulated by the theta phase. These results indicate that the theta-gamma coupling in our spiking neural circuit is an intrinsic, emergent property, unlike existing modeling studies^43^ in which one oscillatory component (i.e., theta) was externally imposed on circuit models that only generate another (i.e., gamma). As illustrated below, gamma bursts orchestrated by theta activity play a crucial functional role in preventing interference when routing multiple stimuli across cortical areas.

### Distributed dynamical communication based on wave pattern interactions

We next elucidate that how the coordinated interactions of the localized wave patterns with rich and complex spatiotemporal dynamics in areas 1 and 2 enable communication to occur in a fundamentally distributed and dynamical way, providing a mechanism that unifies gamma synchrony-based communication and subspace-based communication.

**Fig. 2:**
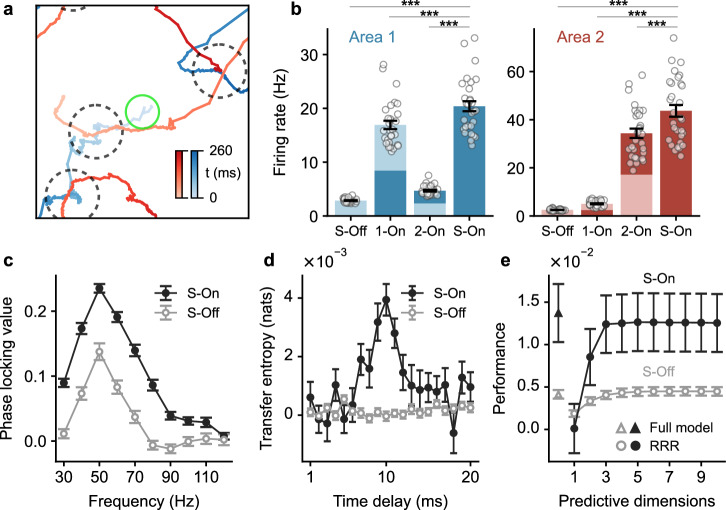
Coordinated interactions of wave patterns in two interconnected cortical areas result in gamma burst and subspace-based interareal communication. **a** Trajectories spanning 260 ms of the wave patterns in areas 1 (blue) and 2 (red) are shown within the same 2D space. Dashed circles mark synchronized events, and the green circle indicates the region of sampled neurons for On-Off states analysis in **b**–**e**. **b** Average firing rates of area 1 neurons (left panel) and area 2 neurons (right panel) sampled at the network center (green circle in **a**) during different states: S-Off (simultaneous-Off), S-On (simultaneous-On), 1-On (only area 1 in On states), 2-On (only area 2 in On states). Dots represent the average rates of individual random network realizations (*n* = 30). ***p < 10^−13^, two-sided paired t-test. Light and dark colors in each bar indicate the Off and On states, respectively, of the center region of each area. For example, the 1-On state is represented by a bar with the bottom half part dark blue/red and the top half part light blue/red. **c** Average phase locking value between MUA at the centers of areas 1 and 2 during S-Off (gray circle) and S-On states (black circle) within the frequency range of 30 to 120 Hz. **d** Average transfer entropy from area 1 to area 2 during S-Off (gray circle) and S-On states (black circle) at various time delays. **e** Average performance of predicting the spiking activity in the center of area 2 using the spiking activity in the center of area 1 through reduced-rank regression (circle) and the full model (triangle) during S-Off (gray) and S-On (black) states. All data in **b**–**e** are presented as averages across random network realizations (*n* = 30 realizations for **b**, **c**, and **e**; *n* = 60 realizations for **d**). Error bars denote ± 1 SEM. Source data can be found in Supplementary Data 1.

By tracking the trajectories of these wave patterns in both areas, we find that once they align spatially at a topographically matched position, which is indicated by those periods when the patterns in both areas are simultaneously close to a topographically aligned position (Fig. 1c and d), they interact due to the cortico-cortical loops and then become synchronized at this location for a period of time. Because of the large jumps and large fluctuations inherent in the Lévy motion, they may rapidly switch to and are synchronized at another location for another transient epoch. These transiently synchronized wave patterns give rise to simultaneous burst spiking activity occurring at the topographically aligned positions across both areas. We denote the epoch of simultaneous burst activity across the areas as the simultaneous On state (S-On) and the epoch of simultaneous faint spiking activity as the simultaneous Off (S-Off) state. As demonstrated above, the spiking bursts are associated with the gamma bursts, suggesting that the aligned spiking bursts might be associated with gamma synchronization. To confirm this, we calculate the phase locking values^44^ (PLV; measured as the mean resultant length of relative phase; see Methods) between MUAs in the centers of the two cortical areas, and find that the gamma band PLV during the S-On state is greater than that during the S-Off state, indicating strong interareal gamma synchronization during the aligned bursts activities (Fig. 2c; average PLV between 40 and 60 Hz is 0.20 ± 5 × 10^−3^ for the S-On state and 0.10 ± 7 × 10^−3^ for the S-Off state, *p* < 10^−12^, mean ± SEM, two-sided paired t-test) and the peak PLV value appears at around 50 Hz which is close to the peak gamma frequency shown in the power spectrum (Fig. 1e).

Due to the superdiffusive Lévy motion nature of the wave patterns, these synchronized events exhibit remarkable flexibility and rapid transitions among spatially distributed neural groups over time. As shown in Fig. 2a, we observe wave pattern synchronization occurring at three different positions in a mere 260 milliseconds (Fig. 2a, dashed circles). This behavior underscores the fundamentally distributed and dynamic property of these synchrony events and the resultant communication; hence, we refer to this communication mechanism, based on wave pattern interactions, as DDC.

The enhanced synchronization occurring in DDC suggests that these synchrony epochs are key time intervals during which the two cortical areas interact effectively. To illustrate such effective interactions, we examine the changes in firing rates for neurons in both areas during and outside these synchrony events (Fig. 2b). Firing rates are highest when both areas are in the On state, compared to when only one is in the On state or both are in the Off state (area 1 firing rate is 20.40 ± 0.94 Hz for S-On, 16.94 ± 0.78 Hz for area 1 On only, 4.69 ± 0.16 Hz for area 2 On only, and 2.86 ± 0.07 Hz for S-Off; area 2 firing rate is 43.67 ± 2.46 Hz for S-On, 5.04 ± 0.22 Hz for area 1 on only, 34.35 ± 1.94 Hz for area 2 On only, and 2.51 ± 0.07 Hz for S-off; mean ± SEM, *p* < 10^−13^ for all the comparisons between the mean at S-On state and other states, two-sided paired t-test). These increased firing rates occur because once spiking bursts in both areas synchronize, the bursting neurons in the lower area (area 1) activate those in the higher area (area 2), which, in turn, send descending activation to the lower area. It is intriguing to note that this scenario of amplification in firing rates bears similarities to the concept of “ignition” proposed to understand cortico-cortical interactions underlying cognitive processing^45^. However, unlike the global ignition involving the entire network, the ignition observed in our model is a local phenomenon, involving only a subset of neurons with neural networks being “sparsely" activated.

To further elucidate that in DDC, these synchronized burst events are related to interareal communication, we perform an information theoretical analysis (see Methods). Specifically, we measure the transfer entropy (TE) between MUAs in the center regions of the two areas in the bottom-up direction for both S-On and S-Off states (Fig. 2d). TE quantifies how much information can be provided by the past MUA in area 1 in predicting the future MUA in area 2. Our analysis reveals that information transfer primarily occurs during the synchronous bursts (S-On), peaking at the time delay of ≈ 10 ms, which is consistent with the interareal spike transmission delay in our model (8–10 ms, see Methods). In contrast, information transfer is low outside the synchronous epochs (S-Off) (at 10 ms time delay, TE = 0.004 ± 5 × 10^−4^ nats for S-On and TE = − 4 × 10^−5^ ± 2 × 10^−4^ nats for S-Off, mean ± SEM, *p* < 10^−8^, two-sided paired t-test; note that although the TE is theoretically non-negative, the variance in the TE estimator causes the small negative TE for S-Off).

### DDC enables the flexible formation and reconfiguration of communication subspaces

We next illustrate how the coordinated interactions of localized spiking wave patterns underlying DDC provide a dynamical mechanism for implementing subspace-based communication. To this end, we relate the fluctuations of neuron firing in two areas by linear regression. Specifically, during spontaneous activity, we simultaneously record the number of spikes in 20 ms nonoverlap windows, referred to as single-unit activity, for each neuron in the center regions of both areas. We then divide the single-unit activity time series into On state and Off state periods, and calculate the fluctuations of the single-unit activity for each neuron during these state periods by subtracting the mean single-unit activity of each neuron from the raw single-unit activity in the corresponding state periods. To test whether the fluctuations in area 1 that are predictive of area 2 reside in a low-dimensional subspace, we perform the reduced-rank regression (RRR) as in ref. ^4^ for both S-On and S-Off states (see “Methods” section). RRR is a variant of linear regression that constrains the regression weights into a low-dimensional subspace during fitting. We find that only 3 dimensions are needed for the prediction performance of RRR during S-On states to be comparable to that of the full linear regression model (ridge regression; see Methods; Fig. 2e, black circle; *p* = 0.27 for the difference in the performance at S-On states between the full linear regression model and the RRR with three dimensions, two-sided paired t-test). This result indicates that the communication is realized through a subspace with a low dimension of ≈ 3. During the S-Off state, however, the prediction performance of RRR is much lower compared to the S-On state (gray circle; *p* = 0.017 for the difference between the performance of RRR with 3 dimensions at the S-On state and S-Off state, two-sided paired t-test). These results thus suggest that the synchronized or correlated spiking burst patterns in the two areas underlie the emergence of communication subspace.

Due to the transient nature of burst pattern synchrony, the communication subspace persists for approximately 30 ± 0.72 ms (mean ± SEM). As illustrated above, burst synchrony events can flexibly shift space over time; this property thus enables natural reconfiguration and shifting of communication subspaces among different groups of neurons. The ability to flexibly switch between different neural groups for dynamical communication is a core prediction of our proposed mechanism. It is worth noting that recent investigations have revealed that distinct subsets of neurons in mouse V1 were affected by neurons in LM at different temporal moments, within a timescale of tens of milliseconds^17^, consistent with the dynamical communication mechanism proposed in our modeling study.

Taken together, our results indicate that the DDC mechanism harnesses the realistic and complex spatiotemporal dynamic of wave patterns to enable flexible interareal communication; these dynamics include transient gamma and theta oscillations with the former locked to the latter, arrhythmic 1/*f* activity accompanying these oscillations, and superdiffusive motion of wave patterns in space with heavy-tailed, non-Gaussian (Lévy) statistics, beyond either persistent or transient gamma activity as explored in existing studies. Crucially, the DDC mechanism can explain the subspace-based and gamma synchronization-based communication mechanisms, thus providing a unifying framework for understanding interareal communication.

### DDC underlies flexible interareal communications of neural responses to external inputs

We next illustrate that our DDC mechanism underlies the flexible and rapid routing of external input information across cortical areas and validate the key predictions of our mechanisms by analyzing the Allen Neuropixels data.

**Fig. 3:**
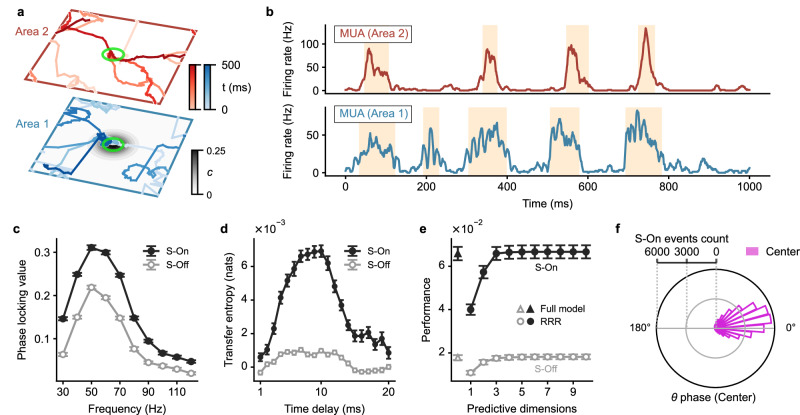
Interareal communication of input signals. **a** Trajectories of wave patterns in area 1 (blue, bottom) and area 2 (red, top) over a 500 ms duration following the introduction of an external input at the center of area 1. The strength of the input (*c*) is represented by varying shades of gray color. The wave patterns intermittently visit the region of the input. The green circle indicates the region of sampled neurons for analyses in **b**–**f**. **b** MUA at the center of area 1 (bottom) and area 2 (top) under the single-input condition. Note that the MUA continues to exhibit On-Off transitions, with the On states indicated by yellow segments. **c** Average PLV between the MUA at the centers of areas 1 and 2 (i.e., the stimulus location) during S-Off (gray circle) and S-On states (black circle) across the frequency range of 30 to 120 Hz. **d** Average TE of the MUA from area 1 to area 2 during S-Off (gray circle) and S-On states (black circle) at different time delays. **e** Average performance of predicting the spiking activity in the center of area 2 using the spiking activity in the center of area 1 through reduced-rank regression (circle) and the full model (triangle) during S-Off (gray) and S-On (black) states. **f** At the middle time point of each S-On state event at the network center (i.e., the location of the input), the theta phase of the MUA in the center of area 1 is recorded and its distribution is shown (*n* = 42, 170). Data in **c**–**e** show the averages across random network realizations (*n* = 60 realizations). Error bars indicate ± 1 SEM. Source data can be found in Supplementary Data 1.

We first add one input at the center of area 1 (Fig. 3a). Following its onset, the wave patterns exhibit prolonged presence around the center locations of both areas compared to spontaneous activity. However, the patterns still intermittently switch to other locations over time with their propagation dynamics following superdiffusive Lévy motion (Supplementary Fig. 7 and 2b). Consequently, spiking activities at the center of areas still exhibit coordinated On-Off transitions (Fig. 3b). For this scenario, consistent with the spontaneous activity, we find that the S-On state exhibits stronger gamma phase locking values (PLV) compared with the S-Off state (Fig. 3c). The average PLV between 40-60 Hz is 0.29 ± 3.5 × 10^−3^ for the S-On state and 0.19 ± 2.9 × 10^−3^ for the S-Off state (*p* < 10^−37^, two-sided paired t-test). Next, we calculate the transfer entropy (TE) as we did for the spontaneous activity. We find that the information is primarily transmitted inside the synchronized bursts (Fig. 3d). The average TE across time delays from 7 to 10 ms is TE = 8.7 × 10^−4^ nats for the S-Off and TE = 6.8 × 10^−3^ nats for the S-On (*p* < 10^−29^, two-sided paired t-test). Additionally, we find that the interareal communication occurs through a low-dimensional subspace preferentially during the S-On state in this stimulus-evoked condition, as revealed by the RRR analysis (Fig. 3e). For the S-On state, the performance for RRR is comparable to that for full linear regression (0.066 ± 0.003, mean ± SEM) when the number of dimensions is 3 (*p* = 0.84, two-sided paired t-test) and is significantly higher than that for S-Off state for all number of dimensions (*p* < 10^−18^). The interareal communication during the S-On state is coordinated by theta oscillations. We illustrate this by examining the occurrence of S-On states with respect to the theta phase of MUA (see Methods). Figure 3f shows that the S-On states primarily occur at the zero phase of the MUA’s theta oscillations (mean phase: 8.76°). This alignment arises because the period of high MUA during the On states corresponds to the zero phase of MUA theta oscillations; thus, simultaneous-On states, during which the local MUA in both areas is high, also tend to occur near the zero theta phase.

We next validate the key properties of DDC uncovered in our modeling study, including theta-gamma coupling and the connection between gamma-burst synchrony and subspace-based interareal communication, through analyzing the Allen Neuropixels visual coding dataset^18^. This dataset provides us with high-resolution electrophysiological recordings from the mouse visual cortex. While we acknowledge that the experimental data cannot capture the full two-dimensional dynamics predicted by our model due to the nature of the recording devices, there remains a strong case for comparison between our analysis of a group of neurons at the center of each area of our model and the recording probes placed at the retinotopic centers of the mouse visual cortical areas in the real data. Specifically, our analysis focuses on interareal interactions between two cortical areas, namely, the primary visual cortex (V1, or VISp) and a secondary visual area, the lateral visual cortex (LM, or VISl), which has been reported to sit adjacent to V1 in the mouse visual hierarchy^18^. We examine the responses of these areas to external stimuli, such as flashes, to substantiate our modeling predictions (see Supplementary Methods 2 for a description of the data, and further details on the methods used in this section).

**Fig. 4:**
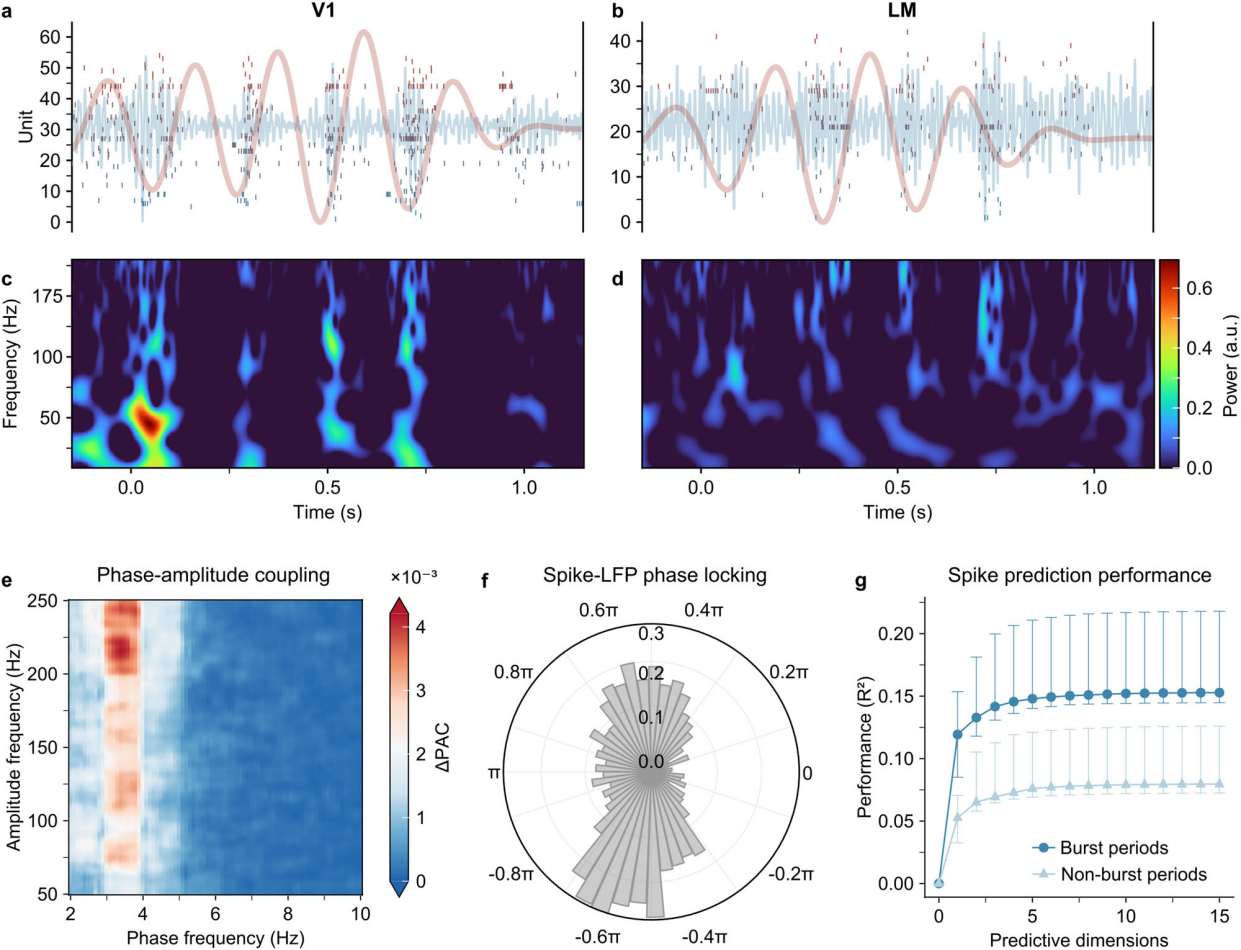
Neuropixels data exhibit the key dynamical properties of interareal interactions as predicted in our model. **a** Snapshots of the Allen Neuropixels visual coding dataset showing spiking activity (vertical bars), and LFP oscillations filtered in the gamma band (50–250 Hz, blue line) and theta band (3–5 Hz, orange line) for one trial of the flashes stimulus in the mouse primary visual area (V1). **b** Same as (**a**) but for the latero-medial visual area (LM). **c** Time-frequency spectrograms of the LFP traces in V1, after detrending the aperiodic component. **d** Same as (**c**) but for the LM. **e** Theta phase–gamma amplitude comodulogram during flash-evoked bursts in V1. **f** Distribution of Hilbert phases at each spike time during evoked theta bursts in V1 (*n* = 830, 723). **g** Median performance across mice when predicting V1 spikes from LM spiking activity using reduced-rank regression during evoked theta bursts (dark circles) and non-burst activity (light triangles). Error bars depict the upper and lower quartiles across 9 mice. Source data can be found in Supplementary Data 1.

As demonstrated in Fig. 4a and b, the neural activity in both V1 and LM responding to full-field flashes exhibits burst-like characteristics, fluctuating between On and Off states. These fluctuations occur approximately four times per second, aligning with the presence of theta oscillations predicted by our model. In the Allen Neuropixels dataset, we find that theta oscillations are transient, typically lasting for a duration between 500 ms to 1000 ms (Fig. 4a and b, red line). The presence of theta activity is further substantiated by a clear theta peak in the power spectrum of experimental local-field potentials (LFP; see Supplementary Fig. 8). Furthermore, Fig. 4a–d illustrates how spiking bursts during the On state are associated with gamma bursts in the 50–250 Hz frequency range, both of which are coupled to the theta oscillations, as predicted in our modeling study. To quantify this theta-gamma coupling, we calculate, as in our modeling study, the average phase-amplitude modulation index for LFP across nine animals. As shown in Fig. 4e, the most pronounced phase-amplitude coupling occurs between the 4Hz theta oscillations and a broad range of gamma frequencies. Similarly, spike bursts are locked to the phases of the theta oscillations (Fig. 4f).

We next examine the gamma-burst-based interareal interactions. For this purpose, as in our modeling study detailed above, we calculate the phase-locking index (PLI, measured as pairwise-phase consistency, see Supplementary Methods 2) for gamma bursts occurring within V1 and LM. Our analysis reveals that the mean phase-locking index between pairs of channels in deep layers of V1 and all channels in LM, over gamma-band frequencies between 40 Hz and 60 Hz, amounts to 0.21 ± 8 × 10^−4^ (mean ± SEM; see Supplementary Fig. 9). This level of phase-locking is significantly greater than what is observed during spontaneous activity, where the mean PLI is 0.11 ± 5 × 10^−4^ (*p* < 10^−100^, one-sided paired t-test, Benjamini-Hochberg corrected). We provide further details on the variation in phase locking across frequencies and cortical depths in Supplementary Fig. 9.

We then investigate how burst synchrony gives rise to the emergence of a low-dimensional communication subspace between visual areas. We apply a similar RRR analysis as in our modeling to the Neuropixels spike data during both theta burst and non-burst periods. To ensure that our analysis focuses on neurons responsive to the visual stimuli, we identify the 20 neurons with the highest firing rates in each region (V1 and LM). Subsequently, we apply a 100 ms-wide Hanning window to convolve spike trains, generating a time course of firing rates. We then use RRR with a regularization parameter of *λ* = 0.1 (matching our earlier methods; our results are not sensitive to changes in *λ* from 0.01–0.5) to predict the neural activity in LM from the activity in V1, and vice versa. As shown in Fig. 4g, our results indicate that spiking activity within LM during burst periods exhibits significantly greater predictability of activity in V1 within a low-dimensional space compared to the non-burst period (*p* < 10^−3^ with a communication dimension of 3, at which the median prediction performance is 91% of its maximum for the burst periods).

**Fig. 5:**
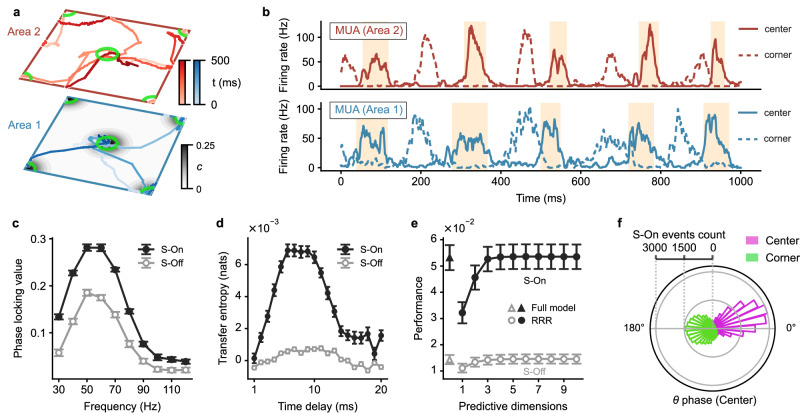
Properties of flexible and rapid routing of multiple inputs across cortical areas. **a** Trajectories of wave patterns in area 1 (blue, bottom) and area 2 (red, top) during a 500 ms interval, with two external inputs added to area 1 (one at the center and another at the corner). Plotting conventions follow those in Fig. 3a. The region of sampled neurons for analyses in **b**–**f** are indicated by the center green circle; for analyses in **b** and **f**, we also analyze the neurons within the corner green circle. **b** MUA at the center (solid line) and corner (dashed line) of area 1 (bottom) and area 2 (top). Bursting activity during On states alternates between the corner and center regions. **c**–**e** Average phase locking value (**c**), transfer entropy (**d**), and prediction performance (**e**) for the two-input condition. Plotting conventions follow those in Fig. 3c–e. Data are presented as the averages across random network realizations (*n* = 30 realizations for **c** and **e**; *n* = 60 realizations for **d**). Error bars denote ± 1 SEM. **f** Distributions of the area 1 center MUA’s theta phases recorded at the middle time point of each S-On state event occurring at the center (pink, *n* = 21, 959) and corner (green, *n* = 22, 723) of the network. Source data can be found in Supplementary Data 1.

We now elucidate that our DDC mechanism is particularly flexible in routing neural responses to multiple competing inputs through the different areas of our large-scale circuit model. For simplicity, here we use two inputs as an example, one at the center and another at the corner of area 1 simultaneously (Fig. 5a; see Methods), but multiple inputs can be similarly routed through the circuit. We find that the localized wave patterns in both areas switch between the two locations with external inputs sequentially, with occasional jumps to other locations (Supplementary Fig. 10); the propagating dynamics can still be characterized as superdiffusive Lévy motion (Supplementary Fig. 2c). As illustrated above, such wave pattern dynamics lead to On-Off transitions in the spiking activity (Fig. 5b). Similar to the scenario with a single external input, we find that the S-On state exhibits stronger gamma PLV and larger information transfer than the S-Off state (Fig. 5c and d), and that the communication still occurs through a low-dimensional subspace (dimension ≈ 3) primarily during the S-On state, as revealed by the RRR analysis (Fig. 5e).

In our DDC mechanism, gamma bursts representing the stimuli at different locations are locked to distinct phases of the theta oscillations. Figure 5f shows that, as in the single-input case, the bursts in the center region primarily happen around the zero phase of the center MUA theta phase (mean phase: 13.06°), while the bursts in the corner region tend to occur at the anti-phase with a broader distribution (mean phase: 187.22°). This result indicates that cross-frequency theta-gamma coupling in our DDC mechanism enhances the segregation of routing different stimuli, effectively preventing interference in interareal communication.

### Neural effects of cued attention emerging from DDC modulated by the interplay between ACh and cortical feedback loops

We next illustrate that the DDC based on wave pattern interactions can be effectively modulated and enhanced during cognitive functions, particularly in the context of cued top-down attention. Importantly, we demonstrate that the dynamical process responsible for such modulations provides a mechanistic account of a great variety of neural effects of attention. These effects include increases in theta-gamma coupling following cue onset^25^, the phenomenon of biased competition^19,20^, as well as reductions in neural variability^22^ and correlation^23,24^. In essence, this presents a novel DDC-based account of cued top-down attention.

**Fig. 6:**
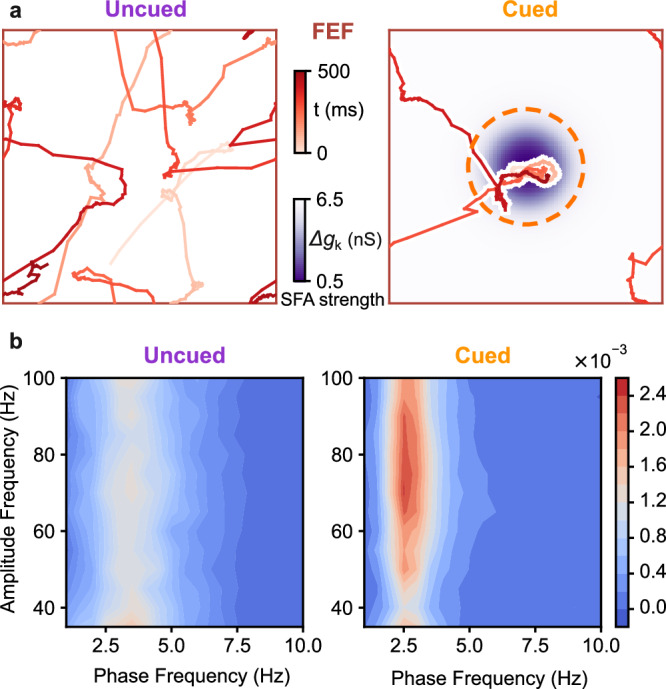
Modulation of wave pattern dynamics and theta-gamma coupling by cue presentation. **a** Trajectories of the wave pattern (red line) and the distribution of SFA strength in the FEF (represented by varying shades of purple color) under uncued (left) and cued conditions (right). In the absence of external inputs, the pattern uniformly traverses the FEF when the SFA is homogeneous (uncued). However, under the cued condition, the wave pattern visits the cued location (orange circle) for a longer duration. **b** Cross-frequency phase-amplitude comodulograms (averaged across 30 random network realizations) for the LFP at the FEF center during the uncued (left) and cued (right) conditions as shown in (**a**). Source data can be found in Supplementary Data 1.

We consider a cued attention task, in which a cue is given for one of two simultaneously presented objects that are monitored to detect a change in either object^23,46^. For this task, in our circuit model, we designate the lower area 1 as V4 (a sensory area) and the higher area 2 as FEF (an association area); indeed, there is experimental evidence showing direct connections between these two cortical areas^47^. In our attention model, two stimuli are presented to the V4 area as in the 2-input condition described above. To incorporate the effect of the cue, we take note of experimental findings indicating that cues can trigger the release of neuromodulator acetylcholine (ACh) to the frontal area^25,48^. The release of ACh can quickly target localized neural populations^49^, thus facilitating cue-driven attentional mechanisms. For this reason, Schmitz *&* Duncan^50^ proposed that ACh may serve as a key biochemical substrate underlying the rapid population coding dynamics of attention. Furthermore, we notice that among the various effects attributed to ACh, its release has been observed to reduce spike-frequency adaptation (SFA) mediated by potassium channels^51,52^. Synthesizing these empirical observations, our model assumes that the cue induces a spatially localized reduction of SFA in the excitatory neurons of FEF in the local region topographically aligned with the cue (Fig. 6a); thus, the initiation of localized SFA reduction indicates the onset of the cue in our model.

To proceed, we first demonstrate that the effect of cue-triggered ACh on the dynamics of the FEF area is consistent with experimental findings. Howe et al.^25^ has shown that even in the absence of stimulus, cue-triggered ACh can enhance theta-gamma coupling in the prefrontal cortex. To illustrate that our model can capture such enhanced theta-gamma coupling, we calculate the phase-amplitude coupling modulation index (PAC-MI) for FEF local field potential at the cued location, both before and after the cue onset without any external input in V4. As shown in Fig. 6b, the theta-gamma phase-amplitude coupling is stronger in the cued condition than in the spontaneous activity [average peak PAC-MI over all amplitude frequency (35–100 Hz)-phase frequency (1–10 Hz) combinations is 3.9 × 10^−3^ for cued and 2.7 × 10^−3^ for spontaneous, *p* < 6 × 10^−5^, two-sided paired t-test]. This result thus provides further neurophysiological validity to our model of ACh-mediated modulation of interareal communication.

**Fig. 7:**
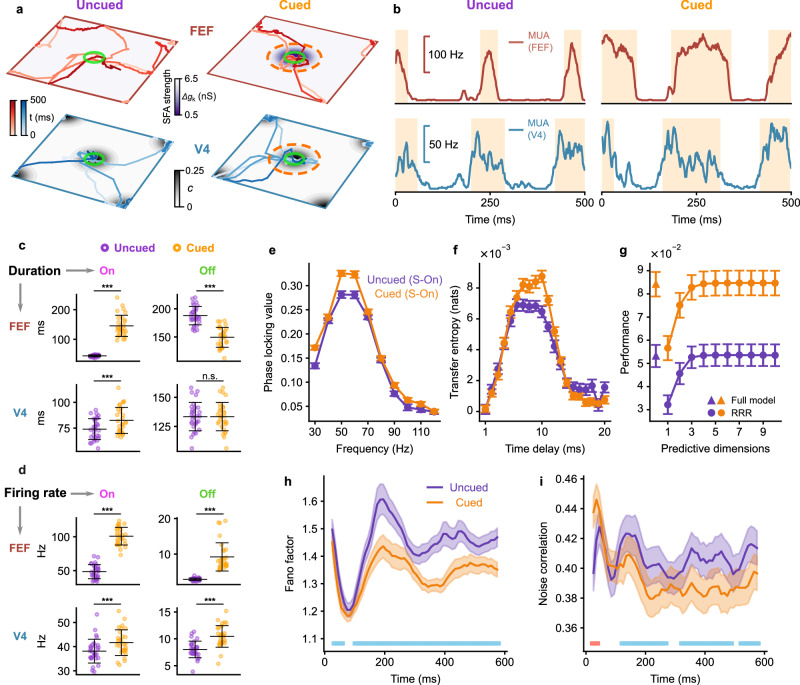
Key neural features of visual attention emerging from DDC modulation. **a** Trajectories of the wave pattern in V4 (bottom, blue) and FEF (top, red) under the uncued (left) and cued (right) conditions when two external inputs are placed at the center and corner of V4 (black). The strength of SFA in area 2 is represented by the shades of purple color. The dashed circle indicates the cue location. The green circle indicates the region of sampled neurons for analyses in **b**–**i**. **b** MUA at the center of V4 (bottom) and FEF (top) under the uncued (left) and cued (right) conditions. **c** Average durations of On states (left column) and Off states (right column) under the uncued (purple) and cued (orange) condition in V4 (bottom row) and FEF (top row). *n* = 30 network realizations. Error bars represent ± 1SD. ***p < 0.001, two-sided paired t-test. **d** Same as (**c**) but for the average firing rate. **e** Average PLV between MUA at the center of V4 and FEF (the location of the center stimulus) during the S-On state for the uncued (purple) and cued (orange) conditions, across the frequency range of 30–120 Hz. **f** Average TE of the MUA from V4 to FEF during the S-On state for the uncued (purple) and cued (orange) conditions, calculated at different time delays. **g** Average performance in predicting spiking activity at FEF center using spiking activity at V4 center through the reduced-rank regression (circle) and the full model (triangle) during the S-On state for the uncued (purple) and cued (orange) conditions. **h** Average mean-matched Fano factor of a local group of excitatory V4 neurons near the center input after its onset. The horizontal blue line indicates the period when the cue significantly reduces the Fano factor (*p* < 0.05, one-sided paired t-test). **i** Same as (**h**) but for the noise correlation of the same group of neurons. The horizontal blue (red) line indicates the period when the cue significantly reduces (increases) the noise correlation (*p* < 0.05, one-sided paired *t*-test). Data in **e**–**i** represents the average results across random realizations of networks (*n* = 30 for **e** and **g**; *n* = 60 for **f**, **h**, and **i**). Error bars represent ± 1 SEM. Source data can be found in Supplementary Data 1.

We now demonstrate that the interplay of the neuromodulator ACh and cortical feedback enables an effective modulation of coordinated interactions of the wave pattern and resultant DDC, enhancing interareal communication during cued attention. To this end, we compare the conditions with and without local SFA reduction in the center area of FEF, with the former referred to as the cued condition and the latter as uncued condition, respectively. In both conditions, two external inputs are presented: one at the center and the other at the corner of V4 (Fig. 7a). In the cued condition, we find that the localized wave pattern in FEF would stay in the central location with ACh modulation for a longer duration due to ACh modulation, which decreases SFA (Supplementary Fig. 11, Supplementary Movie 2). Consequently, the On state duration is prolonged, and the firing rate at the cued position in FEF is higher than the uncued condition (Fig. 7b-d). Specifically, the uncued condition exhibits a mean On duration (*t*_on_) of 44.14 ± 0.60 ms, whereas the cued condition demonstrates an extended On duration of 145.45 ± 6.54 ms (mean ± SEM; *p* < 10^−5^, two-sided paired t-test). Similarly, the uncued condition has a mean On rate (*r*_on_) of 48.49 ± 1.90 Hz, while the cued condition displays a higher On rate of 100.93 ± 2.38 Hz (mean ± SEM; *p* < 10^−5^, two-sided paired t-test).

Because of the feedback interactions, the localized activity pattern in FEF with the prolonged duration at the cued location would tend to drag the localized pattern in V4 to stay around the topographically aligned central area for a longer period than the uncued condition. As a result, this process leads to an increase of the On state duration in V4 (Fig. 7b and c; uncued: *t*_on_ = 73.99 ± 1.85 ms, cued: *t*_on_ = 82.48 ± 2.31 ms, mean ± SEM, *p* < 10^−5^, two-sided paired t-test), as found in V4 of monkeys during the cued attention task^21^. The top-down modulation also increases the firing rate of the On and Off state in V4 (Fig. 7d; On rate, uncued: *r*_on_ = 38.13 ± 0.90 Hz, cued: *r*_on_ = 41.65 ± 0.97 Hz, *p* < 10^−5^; Off rate, uncued: *r*_off_ = 8.02 ± 0.28 Hz, cued: *r*_off_ = 10.44 ± 0.37 Hz, *p* < 10^−5^, two-sided paired t-test), consistent with previous experimental findings^21^. In experimental studies, the effect of top-down attention on the Off duration is unclear, with different experiments obtaining distinct results^21,39^; in our model, we find no significant influence on the Off duration in V4 (uncued: *t*_off_ = 133.41 ± 2.23 ms, cued: *t*_off_ = 131.53 ± 2.82 ms, mean ± SEM, *p* = 0.20, two-sided paired t-test), but a significant reduction in the Off duration in FEF by attention (uncued: *t*_off_ = 187.84 ± 2.98 ms, cued: *t*_off_ = 149.46 ± 3.21 ms, mean ± SEM, *p* < 10^−5^, two-sided paired t-test) (Fig. 7c). In our model, the distribution of the duration of the On and Off state follows the exponential distribution (Supplementary Fig. 12a, c), comparable to On and Off durations measured in V4 of monkeys during cued attention tasks^21^.

The prolonged durations of the wave packets consequently enhance interareal communications between the sensory and association areas. As shown above, we perform gamma synchrony analysis and find that the average PLV of gamma bursts (S-On) between 40-60Hz is significantly increased during the attention task to 0.29 ± 4 × 10^−3^ (Fig. 7e; *p* < 10^−5^; two-sided paired *t*-test); this is consistent with experimental studies showing enhanced gamma synchrony between V4 and FEF during cued attention tasks^8^. In addition, our information theoretical analysis indicates that information flow quantified by TE increases to TE = 8.4 × 10^−3^ nats (S-On) (Fig. 7f; *p* < 10^−3^, two-sided paired t-test).

We also find the prediction performance of RRR for each number of dimensions is improved by attention during S-On states (*p* < 0.02, two-sided paired t-test; Fig. 7g). In addition, during S-On states in the cued condition, the prediction performance of RRR becomes indistinguishable from that of full linear regression model when the dimensions of RRR exceed 3 (subspace dimension = 4), slightly larger than the dimension observed in the absence of attention (dimension = 3), as mentioned above. Nevertheless, we note that RRR with 3 dimensions in the cued condition performs closely to the full linear regression model, achieving 98.4% of its performance. Thus, the dimension of the subspace remains relatively constant (≈3) in both cued and uncued conditions in our circuit model. This suggests that attention enhances communication between distant neuronal populations without affecting the underlying subspace, consistent with experimental findings showing that attention improves the efficacy of information flow between different brain areas without altering the subspace dimension^6^.

Our model also provides a mechanistic account of how the classical observation of biased competition of attention is implemented in large-scale neural circuits. According to biased competition^19^, the firing rate of a given object will be reduced when a second object is presented. The allocation of attention to the first object will restore its firing rate. To verify biased competition in our model, we compare the neuronal response to one input with that of two concurrently presented inputs. As shown in Supplementary Fig. 13a, compared to introducing only one input at the network center, we find that adding an additional input at nearby position reduces the response in V4 to the original center input from 27.36 ± 0.77 Hz to 23.15 ± 0.70 Hz (mean ± SEM; *p* < 10^−24^, two-sided paired t-test; green vs purple). This reduction occurs due to the lateral inhibition between the response to each input. Specifically, adding an additional input decreases the pattern’s probability to visit the original center input (Supplementary Fig. 13b), resulting in an average reduction in the firing rate at the center input. However, this reduction can be selectively compensated by allocating the cue to the center input, with the response increasing from 23.15 ± 0.70 Hz to 28.12 ± 0.86 Hz (*p* < 10^−25^; purple vs orange). As demonstrated above, triggered by cue, the pattern in FEF spends longer time dwelling the cued location, which increases the burst duration in FEF (Fig. 7c and Supplementary Fig. 13c). Due to the cortical feedback interactions, the top-down inputs to the V4 cued region are enhanced, increasing the probability that the V4 pattern visits the cued input (Supplementary Fig. 13b), and on average, leading to an increase in firing rate for the cued input.

During the cued attention task, it is important to note that the localized wave patterns in both V4 and FEF areas continue to exhibit superdiffusive Lévy displacement in space (Supplementary Fig. 2d). This indicates that that the fundamental stability of cortical circuits remains largely intact and stays within the dynamical working regime. However, what changes during the cued attention task is the local modulation of wave pattern dynamics through an interplay between acetylcholine (ACh) and cortical feedback. This modulation primarily enhances the dwelling time of these wave patterns at the cued location. Due to the intrinsic long jumps inherent in Lévy motion, the wave patterns still possess the potential to occasionally shift to other locations and synchronize there. This mechanism effectively prevents an excessive concentration of interareal communication solely on the cued object, thus preserving flexibility in interareal interactions.

Taken together, these results demonstrate that flexible cognitive functions, such as cued top-down attention, emerge from the modulation of DDC based on wave pattern interactions, and that the interplay between neuromodulators such as acetylcholine and cortical feedback plays an essential role in facilitating this modulation.

### Reductions in neural variability and correlation

We next illustrate that the coordinated spatiotemporal dynamics underlying the modulated DDC provide an explanation for the reductions in neural variability and correlation commonly observed during attention tasks^23,24^. We find that the neurons near the cued location in the V4 area of our circuit model exhibit an attention-induced reduction in neural variability, which is quantified by the mean-matched Fano factor (see Methods). On average across the 600 ms response period following the cue onset, the Fano factor is reduced by 5%, declining from 1.45 to 1.34 (*p* < 10^−13^, two-sided paired t-test). Upon further investigation into the time-resolved Fano factor values, we note that this reduction primarily happens during the later phase of the response (Fig. 7h), which is in line with experimental observations^23^. We then calculate the correlated variability in spike counts of V4 neurons, commonly referred to as noise correlation, between pairs of neurons across trials; we find an attention-related reduction in noise correlation, with a decrease from 0.41 to 0.39, averaged across the response period (*p* < 10^−3^, two-sided paired t-test). Similar to the Fano factor, the temporal profile of noise correlation demonstrates that the significant attention-related reduction of the noise correlation is also evident during the later phase (Fig. 7i).

The localized wave patterns in our model represent a substantial source of correlated neural variability. The modulation of these wave pattern dynamics, as demonstrated in the context of attention tasks, can thus account for the observed reductions in correlated variability. To elucidate these aspects, we employ a mathematical model capable of capturing the complex spatiotemporal dynamics of these localized wave patterns. The model is described by a stochastic differential equation driven by Lévy motion with a momentum term^31^

1 $$d{{{{{{{{\bf{x}}}}}}}}}_{t}=\gamma b\left({{{{{{{{\bf{x}}}}}}}}}_{t}\right)dt+\beta {{{{{{{{\bf{v}}}}}}}}}_{t}dt+{\gamma }^{1/\alpha }d{L}_{t}^{\alpha },$$

2 $$d{{{{{{{{\bf{v}}}}}}}}}_{t}=\beta b\left({{{{{{{{\bf{x}}}}}}}}}_{t}\right)dt,$$

where **x**_*t*_ is the coordinate of the pattern trajectory, **v**_*t*_ is the momentum term, *β* is the damping coefficient, $b\left({{{{{{{{\bf{x}}}}}}}}}_{t}\right)$ is the drift term related to the energy (probability) landscape (see below and Methods), *γ* = 100 is the strength of the noise, ${L}_{t}^{\alpha }$ is the Lévy motion with step sizes over a time period *dt* = 1 ms following a symmetric alpha stable distribution $S\alpha S\left(\alpha ,\,d{t}^{\frac{1}{\alpha }}\right)$, possessing a power-law tail with a tail index 1 < *α* ≤ 2. The momentum term **v**_*t*_ is responsible for generating temporal oscillations in the trajectory of the pattern, with the frequency of the oscillations controlled by the damping coefficient *β*.

**Fig. 8:**
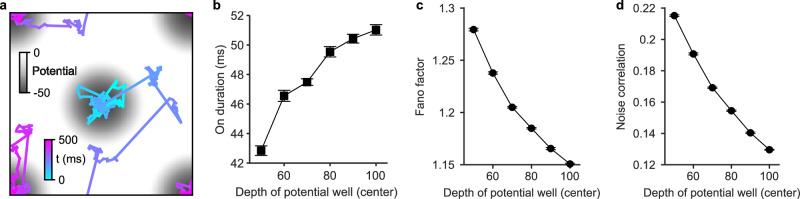
A mathematical model explaining the attention modulation of correlated neural variability. **a** Trajectory of a random walker of the mathematical model over a duration of 500 ms. Two potential wells with equal depth (50) are located at the center and corner. The value of potential is represented by the shades of the gray color. **b** Average On duration at the center well as a function of the depth of the center potential well, representing different strengths of top-down inputs. Error bars denote ± 1 SEM (*n* = 20 trials). **c** Average Fano factor of 80 neurons within the center potential well as a function of the center potential well depth. Data shows the grand average across 20 trials; error bars denote ± 1 SEM. **d** Same as **c** but for the noise correlation. Source data can be found in Supplementary Data 1.

In the mathematical model, we assign the tail index to *α* = 1.2, similar to the tail index characterizing the superdiffusive Lévy motion of localized wave patterns emerging in the neural circuit model (Supplementary Fig. 2). Additionally, we set *β* = 1 to capture their oscillatory aspect (i.e., theta oscillations); other values close to these would generate qualitatively similar results. When devoid of external inputs, the pattern or random walker governed by this mathematical formulation traverses a flat energy landscape. Incorporating two input objects into the model is mathematically equivalent to introducing two wells to the landscape: one at the center and another at the corner (see “Methods” section; Fig. 8a and Supplementary Fig. 14a).

As demonstrated above, the top-down inputs triggered by the neuromodulator ACh increase the probability that the wave pattern visits the cued position. In the mathematical model, this effect is modeled as deepening the potential well at the cued location (Supplementary Fig. 14d). We find that deepening the potential well at the center-cued location in the mathematical model leads to an increase in the duration of the pattern at the corresponding position (Fig. 8b). Nevertheless, the pattern’s global behavior continues to exhibit superdiffusive characteristics. These results from the mathematical model are consistent with the spiking neural circuit model, thus providing further confirmation that during attention tasks, the wave pattern’s state undergoes local modulation while retaining its inherent spatiotemporal attributes.

To quantitatively assess the spiking activity within this mathematical construct, we assume that the instantaneous firing rate profile in the network conforms to a two-dimensional Gaussian bump centered on the trajectory of the pattern, plus a baseline firing rate (see “Methods” section; Supplementary Fig. 14b and c). To model the attention-induced firing rate increase at the cued position, we allow the baseline firing rate at the cued potential well to increase with the potential well depth. We then examine the modulation of the spiking activity in this mathematical model by computing the Fano factor and noise correlation for a group of neurons in the cued region under different top-down input strengths, corresponding to different well depths in the model. As shown in Fig. 8c, the increasing depths result in a decreasing Fano factor from 1.28 ± 6 × 10^−3^ (mean ± SD, uncued; well depth = 50) to 1.15 ± 3 × 10^−3^ (well depth = 100) (*p* < 10^−20^, two-sided unpaired t-test); this means that stronger top-down effects lead to a greater reduction in neural variability. We find that the noise correlation shows a similar trend of reduction (Fig. 8d), with its value decreasing from 0.22 ± 2 × 10^−3^ to 0.13 ± 1 × 10^−3^ (*p* < 10^−18^, two-sided unpaired t-test). Taken together, these results thus indicate that the local modulation of wave pattern dynamics, while preserving their inherent spatiotemporal properties, can account for the observed reductions of neural variability and correlation.

## Discussion

In this study, we have introduced a flexible interareal communication mechanism (i.e., DDC) to understand the functional interactions among different cortical areas and their fundamental roles in cognitive processes such as attention. DDC harnesses realistic, complex spatiotemporal dynamics and their coordinated interactions to communicate information efficiently and flexibly between distinct cortical areas. These coordinated interactions not only give rise to gamma burst-mediated communication but also provide a mechanistic account of the flexible formation and reconfiguration of communication subspaces. As a result, the DDC mechanism unifies gamma-based and subspace-based views, significantly advancing our understanding of interareal communication. As we have illustrated, our DDC mechanism provides profound functional advantages, such as rapid and flexible switching between spatially distributed communication subspaces. This facilitates spatial and temporal multiplexing, enabling flexible and efficient routing of neural responses to multiple objects through cortical areas. In addition, we have elucidated that the dynamical processes underlying modulated DDC account for a great variety of neural effects observed during cued attention tasks, thus revealing the functional significance of DDC in cognition. Our DDC model generates novel testable predictions about interareal communication, which have been confirmed through the analysis of the Allen Institute Neuropixels dataset.

Our DDC mechanism provides a novel perspective on the roles of realistic complex dynamics of neural population activity, unfolding in both time and space, in cortico-cortical interactions; this extends the existing models primarily based on temporal correlation/synchrony of either sustained^3^ or bursting oscillations^14^. In particular, as we have illustrated, localized wave patterns (i.e. wave packets) and their interactions serve as neural substrates for implementing DDC. These wave patterns exhibit rich spatiotemporal dynamics that can capture and explain a great variety of neural dynamics. They hover around one location for a while and then move or switch to another location in an intermittent manner. This propagation of wave patterns can be characterized as a type of nonstationary motion (i.e., Lévy motion). Propagating wave patterns have been ubiquitously observed at both circuit and whole-brain levels^15,16^. Notably, it has been demonstrated that Lévy motion underlies the propagation of neural activity patterns in the MT area of marmoset monkeys^36^. Additionally, neural activity patterns in the hippocampus exhibit hallmark features of super-diffusive Lévy motion^37,38^; in fact, a super-diffusive mathematical model has been employed to model such motions in ref. ^53^.

As we have illustrated, when localized spiking wave patterns shift to a specific location, the local field potential (LFP) in that area exhibits transient gamma bursts, which are nested within theta oscillations. These oscillations, instead of being regular clock cycles of some kind, exhibit substantial variability and non-stationary properties, coexisting with aperiodic 1/*f* fluctuations^35^. Such variable features of neural oscillations have consistently been observed in neural population activity across various recording modalities, whether during spontaneous activity or task-related conditions^11,13,54,55^. Rather than being considered noise or detrimental to cognitive processing, as conventionally assumed, these variable features represent essential functional characteristics of DDC. In our large-scale circuit model of DDC, once wave packets with complex spatiotemporal dynamics in interconnected cortical areas become topographically aligned, they engage in interactions through feedforward-feedback loops. As we have demonstrated, these interactions give rise to the synchrony (phase locking) of gamma bursts, which are crucial for transferring information as quantified by information theory analysis. This indicates that synchronized bursts play an essential role in coordinating interareal interactions and communication. It is interesting to note that during these interactions of wave packets, the firing rates of local groups (a subset) of neurons in both areas undergo significant amplification, displaying behavior reminiscent of “ignition”. The relevance of ignition in DDC supports the hypothesis that ignition plays an essential role in brain functions, particularly conscious stimulus processing^56^. In addition, as we have illustrated, the interactions of wave packets provide a dynamic circuit mechanism for explaining the emergence of communication subspaces^4^. Thus, DDC unifies the two prominent perspectives (gamma-based and subspace-based) for understanding interareal communication.

Crucially, the rich spatiotemporal dynamics displayed by these wave patterns, including occasional long jumps inherent to their superdiffusive propagation motion, endow them with the ability to rapidly shift to other locations. This propagation property, as shown in our previous study, offers a potent solution to the long-standing challenge of sampling and representing multimodal probabilistic distributions^31^. In the context of interareal interactions, this property allows the localized wave patterns in different cortical areas to momentarily align at various distributed locales, naturally giving rise to transitions between distinct synchronized neural groups and communication subspaces; it is important to note that such transitions occur rapidly, often within tens of milliseconds, a time scale relevant to behavior. The DDC model, based on wave pattern interactions, thus provides a mechanism for flexible and rapid transitions between communication subspaces; this capacity constitutes a hallmark feature of DDC and forms a key testable prediction. It is worth noting that in ref. ^17^, casual manipulation of two cortical areas along the cortical hierarchy (V1 and LM) in the mouse cortex was conducted — it was observed that the patterns of influence on the target population can change rapidly. Different subsets of neurons were affected at distinct moments in time, typically within ≈ 50 milliseconds. This finding strongly suggests the presence of dynamically switching communication subspaces, as found in our study.

Another key property of DDC is the coordination of gamma burst-based interareal interactions by theta oscillations. In particular, we have demonstrated that gamma bursts, representing different objects, become entrained with distinct phases of theta oscillations. In DDC, this theta-gamma coupling prevents potential interference when routing neural responses to different objects across cortical areas. Previous studies have proposed the role of fast gamma oscillations nested within slower oscillations such as theta or alpha in coordinating interareal interactions^57,58^. However, these studies have often focused on normative models without specifying their underlying neural circuit mechanisms^58^ or assumed the imposition of one oscillatory element externally. For instance, in ref. ^57^, the theta component is introduced as an external input. In contrast, the cross-frequency coupling is an intrinsic and emergent property of our circuit model, without the imposition of any external modulating inputs. Specifically, we have elucidated that neural firing adaptation underlies the genesis of theta oscillations^35^, a neurophysiological mechanism that we have analytically derived and subsequently validated in our spiking neural circuit model. This result, in turn, suggests that biophysical mechanisms capable of modulating neural adaptation, such as acetylcholine^51,52^, might exert modulation over theta oscillations. In light of this theoretical prediction, it is interesting to note that recent studies have revealed the role of acetylcholine in modulating theta oscillations that support memory formation^59^.

DDC also generates novel experimentally testable predictions on the interrelated burst synchrony-based subspace communication and their rich spatiotemporal dynamics. Through analyzing the Allen Neuropixels visual coding dataset^18^, we have found that neural responses to flashes in V1 and LM exhibit spiking bursts that are associated with gamma bursts. These gamma bursts are coupled with theta oscillations and are accompanied by an arrhythmic 1/*f* component, as predicted by our modeling study. These gamma bursts are synchronized, as quantified by the high phase locking index, for transmitting stimulus information across the cortical hierarchy. We have further found that these epochs of synchronized bursts correspond to the emergence of communication subspaces, thus confirming our prediction of their mechanistic relationship. Importantly, recent research has shown that gamma activity plays a pivotal role in facilitating the communication of distinct visual features during perception within the awake mouse visual system^60^. It would be interesting to investigate whether such gamma-based communication is mechanistically linked to dynamical communication subspaces by conducting a similar analysis as in our study. Nevertheless, it is worth pointing out that the limited spatial coverage of Neuropixels recordings precludes the direct examination of propagating wave patterns. To comprehensively illustrate how coordinated interactions of wave patterns underlie interareal communication as proposed in our study, an ideal approach would involve combining imaging studies that cover spatially extended areas with massive multi-unit recordings. Such an approach would enable the visualization and recording of neural activity at both the population and individual neuron levels, allowing for analysis using the methods as employed in our modeling study.

What is the functional significance of the DDC mechanism, which harnesses coordinated interactions among wave packets with rich and complex spatiotemporal dynamics? In the framework of DDC, spiking bursts and their accompanying gamma bursts serve as vital components in facilitating interareal communication. In contrast to sustained activity patterns, the sporadic bursting firing mode exhibit notable energy efficiency^61,62^. As a result, it provides an efficient means for broadcasting information across different cortical regions. It is interesting to note that the concept of packaging information into discrete bursts for efficient distributed communication has parallels in telecommunications^63^. Importantly, as we have demonstrated, DDC provides a flexible and rapid mechanism for the formation and reconfiguration of distinct communication subspaces. This fundamental property enables various sensory inputs to be routed through the cortical hierarchy in a spatial and temporal multiplexing manner. This feature could prove particularly powerful in conveying information across cortical areas, especially in situations characterized by dynamically changing sensory inputs or varying task demands. To further illustrate the computational power of DDC, it would be interesting to extend our model to incorporate these changing conditions, as explored in experimental studies^64^.

The functional significance of DDC is further underscored by our finding that the modulation processes within DDC provide a versatile mechanism for the emergence of flexible cognitive functions, such as cued top-down attention. As we have demonstrated, the modulated dynamics of wave patterns and their interactions result in enhanced communication during attentional processes, characterized by increased synchrony-based and subspace-based information transfer. Notably, increased gamma synchrony has been previously observed in the V4 and FEF regions of the primate brain during attention^8^, alongside attention-driven enhancements in subspace-based communication within the monkey brain^6^. In addition to capturing these enhanced communication phenomena in experimental studies, we have, to the best of our knowledge, provided the first unified account of a wide range of neural features associated with visual attention through modulated wave pattern interactions. These include increases in spike bursts at the attended location^21^, reductions in neural variability^22^, decreases in spike-count correlations^23,24^, enhanced theta-gamma coupling^25,65^, as well as the classical observation of biased competition^20^. Due to this unified account, which would otherwise not be achievable using existing models^66–68^, DDC holds significant conceptual implications for understanding the neural circuit mechanisms underlying attention; rather than being confined to a specific brain region, attention may be better conceptualized as an emergent property arising from modulated DDC across different brain areas.

What drives the modulation of DDC? Our study has revealed that the interplay between neuromodulators, such as acetylcholine (ACh), and feedback projections, constitute an effective driver for modulating cortico-cortical interactions and communication. This mechanism is grounded in empirical evidence demonstrating that cue-triggered ACh release can rapidly target specific neural populations in the frontal area^25,48^, as also noted in ref. ^50^, and that feedback connections are ubiquitous in the brain^69,70^. As in our model, the release of ACh has been observed to reduce spike-frequency adaptation (SFA)^51,52^. This reduction in adaptation mediated by ACh effectively modulates dynamic wave patterns, particularly by extending their durations at the cued location. This, in turn, leads to enhanced communication, providing an explanation for the neural features associated with cued attention, as we have demonstrated. This modulation mechanism of DDC thus unveils the essential role played by ACh in mediating attention, consistent with proposals in ref. ^50^. It also opens intriguing possibilities that regulating the adequate levels of ACh might be relevant for optimizing attentional performance^71,72^, with deviations in these levels potentially contributing to attention disorders^73^. Given the prevalence of propagating wave patterns^16^, the ubiquity of feedback loops^69^, and the essential roles of ACh in various cognitive processes^74,75^, our findings suggest that the modulated DDC underlying attention, as elucidated in our study, might have broad applications in understanding other cognitive functions.

## Methods

### A spiking neural circuit model involving two cortical areas with feedforward and feedback interactions

The canonical neural circuit model consists of two interconnected cortical areas: area 1 (*β* = 1) and area 2 (*β* = 2), representing regions in the lower and higher cortical hierarchy, respectively. Each area consists of an excitatory neuron group (*α* = e) with *N*_e_ excitatory neurons and an inhibitory neuron group (*α* = i) with *N*_i_ inhibitory neurons; *N*_e_ = 4096 and *N*_i_ = 1024. Here, we use the notation *αβ* to denote a neuron group in area *β* with neuron type *α*, the notation ${m}_{j}^{\alpha \beta }$ to denote a variable *m* (for instance, this can be membrane potential or current) of the *j*^*th*^ neuron in group *αβ*, and the notation ${m}_{{j}^{{\prime} }j}^{{\alpha }^{{\prime} }{\beta }^{{\prime} }\alpha \beta }$ for a variable *m* regarding the connection between the ${j}^{{\prime} th}$ presynaptic neuron from group ${\alpha }^{{\prime} }{\beta }^{{\prime} }$ and the *j*^*th*^ postsynaptic neuron in group *αβ*. The membrane potential ${V}_{j}^{\alpha \beta }$ of the *j*^*th*^ neuron in group *αβ* follows:

3 $$C\frac{d{V}_{j}^{\alpha \beta }\left(t\right)}{dt}=-\,{g}_{{{{{{{{\rm{L}}}}}}}}}\,\left({V}_{j}^{\alpha \beta }\left(t\right)\,-\,{V}_{{{{{{{{\rm{L}}}}}}}}}\right)+{I}_{j,{{{{{{{\rm{k}}}}}}}}}^{\alpha \beta }\left(t\right)+{I}_{j,{{{{{{{\rm{rec}}}}}}}}}^{\alpha \beta }\left(t\right)+{I}_{j,{{{{{{{\rm{ext}}}}}}}}}^{\alpha \beta }\left(t\right),$$

where *α* ∈ {e, i}, *β* ∈{1, 2}, *j* ∈ {1, 2, …, *N*_*α*_}, the membrane capacitance *C* = 0.25 nF, the reversal potential for the leak current *V*_L_ = −70 mV, and the leak conductance *g*_L_ = 16.7 nS and 25 nS for excitatory and inhibitory neurons, respectively. When the membrane potential reaches the threshold *v*_T_ = −50 mV, a spike is generated, and the membrane potential is reset to *v*_r_ = −70 mV for a refractory period *τ*_ref_ = 4 ms. The potassium current for the spike-frequency adaptation of excitatory neurons is

4 $${I}_{j,{{{{{{{\rm{k}}}}}}}}}^{{{{{{{{\rm{e}}}}}}}}\beta }\left(t\right)=-{g}_{j,{{{{{{{\rm{k}}}}}}}}}^{{{{{{{{\rm{e}}}}}}}}\beta }\left(t\right)\left({V}_{j}^{{{{{{{{\rm{e}}}}}}}}\beta }\left(t\right)-{V}_{{{{{{{{\rm{k}}}}}}}}}\right),$$

where the reversal potential *V*_k_ = −85 mV. The dynamics of the potassium conductance are described by

5 $$\frac{d{g}_{j,{{{{{{{\rm{k}}}}}}}}}^{{{{{{{{\rm{e}}}}}}}}\beta }\left(t\right)}{dt}=-\frac{{g}_{j,{{{{{{{\rm{k}}}}}}}}}^{{{{{{{{\rm{e}}}}}}}}\beta }\left(t\right)}{{\tau }_{{{{{{{{\rm{k}}}}}}}}}}+{{\Delta }}{g}_{{{{{{{{\rm{k}}}}}}}}}^{{{{{{{{\rm{e}}}}}}}}\beta }\,{\sum}_{i}\delta \left(t-{t}_{j,i}^{{{{{{{{\rm{e}}}}}}}}\beta }\right)$$

with *τ*_k_ = 60 ms the decay time constant and ${t}_{j,i}^{{{{{{{{\rm{e}}}}}}}}\beta }$ the time of the *i*^*th*^spike generated by neuron *j* from group e*β*. Each spike emitted by an excitatory neuron increases the conductance ${g}_{j,{{{{{{{\rm{k}}}}}}}}}^{{{{{{{{\rm{e}}}}}}}}\beta }$ by ${{\Delta }}{g}_{{{{{{{{\rm{k}}}}}}}}}^{{{{{{{{\rm{e}}}}}}}}\beta }$, with ${{\Delta }}{g}_{{{{{{{{\rm{k}}}}}}}}}^{{{{{{{{\rm{e1}}}}}}}}}$ = 1.9 nS in area 1 and ${{\Delta }}{g}_{{{{{{{{\rm{k}}}}}}}}}^{{{{{{{{\rm{e2}}}}}}}}}$ = 6.5 nS in area 2. We only include spike-frequency adaptation for excitatory neurons, i.e., ${I}_{j,{{{{{{{\rm{k}}}}}}}}}^{{{{{{{{\rm{i}}}}}}}}\beta }\left(t\right)$ = 0 mA for all inhibitory neurons, as spike-frequency adaptation has primarily been observed in pyramidal neurons^76^. The postsynaptic current of intra- and inter-areal recurrent connections is given by:

6 $${I}_{j,{{{{{{{\rm{rec}}}}}}}}}^{\alpha \beta }\left(t\right)=-{\sum}_{{\beta }^{{\prime} }}{\sum}_{{\alpha }^{{\prime} }}{\sum}_{{j}^{{\prime} }}{g}_{{j}^{{\prime} }j,{{{{{{{\rm{rec}}}}}}}}}^{{\alpha }^{{\prime} }{\beta }^{{\prime} }\alpha \beta }\left(t\right)\left({V}_{j}^{a\beta }\left(t\right)-{V}_{{{{{{{{\rm{rev}}}}}}}}}^{{\alpha }^{{\prime} }}\right)$$

where ${g}_{{j}^{{\prime} }j,{{{{{{{\rm{rec}}}}}}}}}^{{\alpha }^{{\prime} }{\beta }^{{\prime} }\alpha \beta }\left(t\right)$ is the postsynaptic conductance for the connection between presynaptic neuron ${n}_{{j}^{{\prime} }}^{{\alpha }^{{\prime} }{\beta }^{{\prime} }}$ and postsynaptic neuron ${n}_{j}^{\alpha \beta }$. The reversal potential for excitatory and inhibitory postsynaptic current are ${V}_{{{{{{{{\rm{rev}}}}}}}}}^{{{{{{{{\rm{e}}}}}}}}}$ = 0 mV and ${V}_{{{{{{{{\rm{rev}}}}}}}}}^{{{{{{{{\rm{i}}}}}}}}}$ = -80 mV, respectively. The kinetics of postsynaptic conductance are described by two coupled differential equations:

7 $$\frac{d{g}_{{j}^{{\prime} }j,{{{{{{{\rm{rec}}}}}}}}}^{{\alpha }^{{\prime} }{\beta }^{{\prime} }\alpha \beta }\left(t\right)}{dt}=-\frac{{g}_{{j}^{{\prime} }j,{{{{{{{\rm{rec}}}}}}}}}^{{\alpha }^{{\prime} }{\beta }^{{\prime} }\alpha \beta }\left(t\right)}{{\tau }_{{{{{{{{\rm{d}}}}}}}}}^{{\alpha }^{{\prime} }}}+\frac{{x}_{{j}^{{\prime} }j,{{{{{{{\rm{rec}}}}}}}}}^{{\alpha }^{{\prime} }{\beta }^{{\prime} }\alpha \beta }\left(t\right)}{{\tau }_{{{{{{{{\rm{d}}}}}}}}}^{{\alpha }^{{\prime} }}},$$

and

8 $$\frac{d{x}_{{j}^{{\prime} }j,{{{{{{{\rm{rec}}}}}}}}}^{{\alpha }^{{\prime} }{\beta }^{{\prime} }\alpha \beta }\left(t\right)}{dt}=-\frac{{x}_{{j}^{{\prime} }j,{{{{{{{\rm{rec}}}}}}}}}^{{\alpha }^{{\prime} }{\beta }^{{\prime} }\alpha \beta }\left(t\right)}{{\tau }_{{{{{{{{\rm{r}}}}}}}}}^{{\alpha }^{{\prime} }}}+{w}_{{j}^{{\prime} }j,{{{{{{{\rm{rec}}}}}}}}}^{{\alpha }^{{\prime} }{\beta }^{{\prime} }\alpha \beta }\,{\sum}_{i}\delta \left(t-{t}_{{j}^{{\prime} },i}^{{\alpha }^{{\prime} }{\beta }^{{\prime} }}-{d}_{{j}^{{\prime} }j}^{{\alpha }^{{\prime} }{\beta }^{{\prime} }\alpha \beta }\right),$$

where ${t}_{{j}^{{\prime} },i}^{{\alpha }^{{\prime} }{\beta }^{{\prime} }}$ is the time of the *i*^*th*^ spike emitted by the presynaptic neuron ${n}_{{j}^{{\prime} }}^{{\alpha }^{{\prime} }{\beta }^{{\prime} }}$, and ${d}_{{j}^{{\prime} }j}^{{\alpha }^{{\prime} }{\beta }^{{\prime} }\alpha \beta }$ is the synaptic time delay between pre- and post-synaptic neuron, which is uniformly sampled between 0.5 ms and 2.5 ms for intra-areal connections (${\beta }^{{\prime} }=\beta$) and between 8 ms and 10 ms for inter-areal connections (${\beta }^{{\prime} }\ne \beta \,$). ${w}_{{j}^{{\prime} }j,{{{{{{{\rm{rec}}}}}}}}}^{{\alpha }^{{\prime} }{\beta }^{{\prime} }\alpha \beta }$ is the synapse coupling weight, which is the amount of increase in ${x}_{{j}^{{\prime} }j,{{{{{{{\rm{rec}}}}}}}}}^{{\alpha }^{{\prime} }{\beta }^{{\prime} }\alpha \beta }\left(t\right)$ for each spike generated by the presynaptic neuron. The dynamics of ${x}_{{j}^{{\prime} }j,{{{{{{{\rm{rec}}}}}}}}}^{{\alpha }^{{\prime} }{\beta }^{{\prime} }\alpha \beta }\left(t\right)$ give rise to a rising-and-decaying time course of postsynaptic conductance ${g}_{{j}^{{\prime} }j,{{{{{{{\rm{rec}}}}}}}}}^{{\beta }^{{\prime} }{\beta }^{{\prime} }\alpha \beta }\left(t\right)$, with the rising time constant ${\tau }_{{{{{{{{\rm{r}}}}}}}}}^{{{{{{{{\rm{i}}}}}}}}}$ = ${\tau }_{{{{{{{{\rm{r}}}}}}}}}^{{{{{{{{\rm{e}}}}}}}}}$ = 1 ms and the decay constant ${\tau }_{{{{{{{{\rm{d}}}}}}}}}^{{{{{{{{\rm{e}}}}}}}}}$ = 5 ms and ${\tau }_{{{{{{{{\rm{d}}}}}}}}}^{{{{{{{{\rm{i}}}}}}}}}$ = 4.5 ms for both areas. Each neuron receives external input current ${I}_{j,{{{{{{{\rm{ext}}}}}}}}}^{\alpha \beta }\left(t\right)$ driven by Poisson spike train from which each spike induces excitatory postsynaptic current with the same kinetics as the intra- and inter-areal excitatory postsynaptic current (See ‘External inputs’ below for more details).

Each area covers a 2D space [−32, 32] × [−32, 32] with periodic boundary conditions. Both types of neurons are uniformly distributed in each area, with the distance between adjacent inhibitory neurons being twice that between adjacent excitatory neurons. We refer to the distance between excitatory neurons as 1 grid point (≈7μm). The coordinate of each neuron ${{{{{{{{\bf{y}}}}}}}}}_{j}^{\alpha \beta }=\left({y}_{j,1}^{\alpha \beta },\,{y}_{j,2}^{\alpha \beta }\right)$, where ${y}_{j,1}^{{{{{{{{\rm{e}}}}}}}}\beta }$, ${y}_{j,2}^{{{{{{{{\rm{e}}}}}}}}\beta }\,$ ∈ {−31.5, −30.5, …, 30.5, 31.5} and ${y}_{j,1}^{{{{{{{{\rm{i}}}}}}}}\beta }$, ${y}_{j,2}^{{{{{{{{\rm{i}}}}}}}}\beta }\,$ ∈ { − 30, − 28, …, 28, 30} for both areas. The two areas represent two cortical regions situated on the same cortical surface. To aid visualization and illustrate interareal connections, these areas are depicted as arranged vertically (Fig. 1a). The connection probability *P* between two neurons decays exponentially with the distance between them:

9 $$P={P}_{0}^{{\alpha }^{{\prime} }{\beta }^{{\prime} }\alpha \beta }\exp \left(-{d}_{{j}^{{\prime} }j}^{{\alpha }^{{\prime} }{\beta }^{{\prime} }\alpha \beta }/{\tau }_{P}^{{\alpha }^{{\prime} }{\beta }^{{\prime} }\alpha \beta }\right),$$

where ${P}_{0}^{{\alpha }^{{\prime} }{\beta }^{{\prime} }\alpha \beta }$ is the peak probability, ${d}_{{j}^{{\prime} }j}^{{\alpha }^{{\prime} }{\beta }^{{\prime} }\alpha \beta }=\Vert {{{{{{{{\bf{y}}}}}}}}}_{j{\prime} }^{\alpha {\prime} \beta {\prime} }-{{{{{{{{\bf{y}}}}}}}}}_{j}^{\alpha \beta }\Vert$ is the distance between presynaptic neuron ${n}_{{j}^{{\prime} }}^{{\alpha }^{{\prime} }{\beta }^{{\prime} }}$ and postsynaptic neuron ${n}_{j}^{\alpha \beta }$, with the distance measured periodically, and ${\tau }_{P}^{{\alpha }^{{\prime} }{\beta }^{{\prime} }\alpha \beta }$ is the decay constant. The values of ${P}_{0}^{{\alpha }^{{\prime} }{\beta }^{{\prime} }\alpha \beta }$ and ${\tau }_{P}^{{\alpha }^{{\prime} }{\beta }^{{\prime} }\alpha \beta }$ are as follows:

1. Intra-areal connection: ${P}_{0}^{{{{{{{{\rm{e1e1}}}}}}}}}={P}_{0}^{{{{{{{{\rm{e2e2}}}}}}}}}=0.8057$, ${P}_{0}^{{{{{{{{\rm{e1i1}}}}}}}}}={P}_{0}^{{{{{{{{\rm{e2i2}}}}}}}}}=0.6964$, ${P}_{0}^{{{{{{{{\rm{i1e1}}}}}}}}}={P}_{0}^{{{{{{{{\rm{i2e2}}}}}}}}}=0.4088$, ${P}_{0}^{{{{{{{{\rm{i1i1}}}}}}}}}={P}_{0}^{{{{{{{{\rm{i2i2}}}}}}}}}=0.5663$; ${\tau }_{P}^{{{{{{{{\rm{e1e1}}}}}}}}}={\tau }_{P}^{{{{{{{{\rm{e2e2}}}}}}}}}=7.5$, ${\tau }_{P}^{{{{{{{{\rm{e1i1}}}}}}}}}={\tau }_{P}^{{{{{{{{\rm{e2i2}}}}}}}}}=9.5$, ${\tau }_{P}^{{{{{{{{\rm{i1e1}}}}}}}}}={\tau }_{P}^{{{{{{{{\rm{i2e2}}}}}}}}}=19$, ${\tau }_{P}^{{{{{{{{\rm{i1i1}}}}}}}}}={\tau }_{P}^{{{{{{{{\rm{i2i2}}}}}}}}}=19$.
2. Inter-areal connection: ${P}_{0}^{{{{{{{{\rm{e1e2}}}}}}}}}={P}_{0}^{{{{{{{{\rm{e1i2}}}}}}}}}={P}_{0}^{{{{{{{{\rm{e2e1}}}}}}}}}={P}_{0}^{{{{{{{{\rm{e2i1}}}}}}}}}=0.4$; ${\tau }_{P}^{{{{{{{{\rm{e1e2}}}}}}}}}={\tau }_{P}^{{{{{{{{\rm{e1i2}}}}}}}}}={\tau }_{P}^{{{{{{{{\rm{e2e1}}}}}}}}}={\tau }_{P}^{{{{{{{{\rm{e2i1}}}}}}}}}=8$.

As shown above, we use the same connection strategy for the intra-areal connections in both areas, as well as for the inter-areal bottom-up and top-down connections. Neurons that are closer together in spatial coordinates, whether within the same area or across different areas, tend to have more similar receptive fields and feature sensitivities. This suggests that the connections described above lead to interactions between two areas with overlapping receptive fields, indicating retinotopically aligned interactions^77^. In our model, all presynaptic neurons are excitatory for the inter-areal connections but target both inhibitory and excitatory neurons. In addition, for the inter-areal projections, we randomly choose 50% of neurons in each excitatory neuron group as the presynaptic (source) neurons, while all excitatory and inhibitory neurons in another area can be the postsynaptic (target) neurons. Based on the aforementioned parameters of synaptic connections, we can calculate the average number of intra-areal connections received by each neuron in one group from another group (i.e., indegree, *K*_in_): ${K}_{{{{{{{{\rm{in}}}}}}}}}^{{{{{{{{\rm{e}}}}}}}}{\beta }^{{\prime} }{{{{{{{\rm{e}}}}}}}}\beta }$ = 270, ${K}_{{{{{{{{\rm{in}}}}}}}}}^{{{{{{{{\rm{e}}}}}}}}{\beta }^{{\prime} }{{{{{{{\rm{i}}}}}}}}\beta }$ = 350, ${K}_{{{{{{{{\rm{in}}}}}}}}}^{{{{{{{{\rm{i}}}}}}}}{\beta }^{{\prime} }{{{{{{{\rm{e}}}}}}}}\beta }$ = 130 and ${K}_{{{{{{{{\rm{in}}}}}}}}}^{{{{{{{{\rm{i}}}}}}}}{\beta }^{{\prime} }{{{{{{{\rm{i}}}}}}}}\beta }$ = 180, where ${\beta }^{{\prime} }=\beta \in \{1,\,2\}$. The intra-areal synaptic coupling weights ${w}_{{j}^{{\prime} }j,{{{{{{{\rm{rec}}}}}}}}}^{{\alpha }^{{\prime} }{\beta }^{{\prime} }\alpha \beta }$ for each postsynaptic neuron ${n}_{j}^{\alpha \beta }$ are randomly generated from a Gaussian distribution with a mean of

10 $${\overline{W}}_{{j}^{{\prime} }j,{{{{{{{\rm{rec}}}}}}}}}^{{\alpha }^{{\prime} }{\beta }^{{\prime} }\alpha \beta }={J}_{{{{{{{{\rm{rec}}}}}}}}}^{{\alpha }^{{\prime} }{\beta }^{{\prime} }\alpha \beta }/\sqrt{{K}_{{{{{{{{\rm{in}}}}}}}},j}^{{\alpha }^{{\prime} }{\beta }^{{\prime} }\alpha \beta }},$$

and a standard deviation that is 5% of the mean, where ${K}_{{{{{{{{\rm{in}}}}}}}},j}^{{\alpha }^{{\prime} }{\beta }^{{\prime} }\alpha \beta }$ is the indegree of neuron ${n}_{j}^{\alpha \beta }$ for the projections from the group with neuron type ${\alpha }^{{\prime} }$ in the same area ${\beta }^{{\prime} }=\beta$, and

11 $${J}_{{{{{{{{\rm{rec}}}}}}}}}^{{\alpha }^{{\prime} }{\beta }^{{\prime} }\alpha \beta }={\overline{W}}_{{{{{{{{\rm{rec}}}}}}}}}^{{\alpha }^{{\prime} }{\beta }^{{\prime} }\alpha \beta }\frac{{\sum }_{j}{K}_{{{{{{{{\rm{in}}}}}}}},j}^{{\alpha }^{{\prime} }{\beta }^{{\prime} }\alpha \beta }}{{\sum }_{j}\sqrt{{K}_{{{{{{{{\rm{in}}}}}}}},j}^{{\alpha }^{{\prime} }{\beta }^{{\prime} }\alpha \beta }}},$$

where ${\overline{W}}_{{{{{{{{\rm{rec}}}}}}}}}^{{\alpha }^{{\prime} }{\beta }^{{\prime} }\alpha \beta }$ is the overall mean of all synaptic strengths of the connections from neuron group ${\alpha }^{{\prime} }{\beta }^{{\prime} }$ to *αβ*. This setup ensures that the average intra-areal coupling weight of a postsynaptic neuron is inversely proportional to the square root of its indegree^78^. We have ${\overline{W}}_{{{{{{{{\rm{rec}}}}}}}}}^{{{{{{{{\rm{e1e1}}}}}}}}}$ = 7.857 nS, ${\overline{W}}_{{{{{{{{\rm{rec}}}}}}}}}^{{{{{{{{\rm{e1i1}}}}}}}}}$ = 10.847 nS, ${\overline{W}}_{{{{{{{{\rm{rec}}}}}}}}}^{{{{{{{{\rm{i1e1}}}}}}}}}$ = 35.534 nS, ${\overline{W}}_{{{{{{{{\rm{rec}}}}}}}}}^{{{{{{{{\rm{i1i1}}}}}}}}}$ = 45 nS, ${\overline{W}}_{{{{{{{{\rm{rec}}}}}}}}}^{{{{{{{{\rm{e2e2}}}}}}}}}$ = 11 nS, ${\overline{W}}_{{{{{{{{\rm{rec}}}}}}}}}^{{{{{{{{\rm{e2i2}}}}}}}}}$ = 13.805 nS, ${\overline{W}}_{{{{{{{{\rm{rec}}}}}}}}}^{{{{{{{{\rm{i2e2}}}}}}}}}$ = 41.835 nS, ${\overline{W}}_{{{{{{{{\rm{rec}}}}}}}}}^{{{{{{{{\rm{i2i2}}}}}}}}}$ = 50 nS. Note that we endow area 2 with stronger excitatory synaptic strengths than area 1 to emulate the cortical gradient in the excitatory coupling strengths observed in the cortex^30^. For simplicity, the coupling weights for inter-areal connections ($\beta \, \ne \, {\beta }^{{\prime} }$) are randomly generated from identical Gaussian distribution for each postsynaptic neuron without scaling the mean with the number of indegrees; the mean inter-areal coupling weights are ${\overline{W}}_{{{{{{{{\rm{rec}}}}}}}}}^{{{{{{{{\rm{e1e2}}}}}}}}}={\overline{W}}_{{{{{{{{\rm{rec}}}}}}}}}^{{{{{{{{\rm{e1i2}}}}}}}}}$ = 3.656 nS and ${\overline{W}}_{{{{{{{{\rm{rec}}}}}}}}}^{{{{{{{{\rm{e2e1}}}}}}}}}={\overline{W}}_{{{{{{{{\rm{rec}}}}}}}}}^{{{{{{{{\rm{e2i1}}}}}}}}}$ = 0.578 nS, and the standard deviation is 5% of the mean. With these biophysically realistic parameter settings, the circuit models of both cortical areas approach the transition regime between different cortical states, equipped with neural adaptation^31^. In this regime, our model, featuring emergent complex spatiotemporal dynamics of localized wave patterns, can capture a broad spectrum of realistic neural dynamics. These dynamics include transient gamma bursts nested within theta oscillations and coexisting with aperiodic 1/*f* fluctuations, which are commonly observed in neural population activity across various recording modalities, whether during spontaneous activity or task-related conditions^11,13,54,55^. Similar to our model, previous studies have investigated spatially extended, 2D neural circuit models with emergent propagating wave patterns to explain realistic neural dynamics and their computational roles^66,79–81^. Particularly, in Huang et al.^66^, the wave patterns arising from zero spatial frequency mode are global, leading to a spatially uniform modulation of noise correlations. However, our model displays localized wave patterns with a radius of ≈ 100μm, which is largely consistent with the radius (≈170 μm) of localized wave patterns reported in ref. ^36^. During attention tasks, the modulation of such localized wave patterns, resulting from the interplay between acetylcholine (ACh) and cortical feedback, induces local modulation of cortical states and dynamics, as found in ref. ^21^.

All simulations of the spiking network model are performed in the Brian2 simulator^82^ using the Euler method with time step 0.1 ms. For each random network realization, the initial membrane potential of each neuron is randomly sampled between -85 mV and -50 mV, and the connectivity is randomly generated. The duration of the simulations for each result is specified in the following sections.

### External inputs

Each neuron receives external input current ${I}_{j,{{{{{{{\rm{ext}}}}}}}}}^{\alpha \beta }\left(t\right)$ driven by a Poisson spike train with a rate equal to

12 $$\lambda \left({{{{{{{{\bf{y}}}}}}}}}_{j}^{\alpha \beta },t\right)={\lambda }_{{{{{{{{\rm{bg}}}}}}}}}+{\lambda }_{{{{{{{{\rm{sti}}}}}}}}}\left({{{{{{{{\bf{y}}}}}}}}}_{j}^{\alpha \beta },t\right),$$

where *λ*_bg_ = 1600 Hz is the rate of homogeneous background inputs for all neurons and ${\lambda }_{{{{{{{{\rm{sti}}}}}}}}}\left({{{{{{{{\bf{y}}}}}}}}}_{j}^{\alpha \beta },t\right)$ is the rate of external inputs whose spatial profile is a sum of Gaussian functions

13 $${\lambda }_{{{{{{{{\rm{sti}}}}}}}}}\left({{{{{{{{\bf{y}}}}}}}}}_{j}^{\alpha \beta },t\right)={\sum}_{l}{c}_{{{{{{{{\rm{sti}}}}}}}},l}^{\alpha \beta }{\lambda }_{{{{{{{{\rm{bg}}}}}}}}}\exp \left(-\frac{{\left\Vert {{{{{{{{\bf{y}}}}}}}}}_{j}^{\alpha \beta }-{{{{{{{{\bf{y}}}}}}}}}_{{{{{{{{\rm{sti}}}}}}}},l}^{\alpha \beta }\right\Vert }^{2}}{2{\sigma }_{{{{{{{{\rm{sti}}}}}}}}}^{2}}\right),$$

where ${{{{{{{{\bf{y}}}}}}}}}_{{{{{{{{\rm{sti}}}}}}}},l}^{\alpha \beta }$ is the center of the *l*^*th*^ external input, *σ*_sti_ = 6 is the width of each input, and ${c}_{{{{{{{{\rm{sti}}}}}}}},l}^{\alpha \beta }$ is the contrast of the *l*^*th*^ input. We add external inputs only to the excitatory neurons in area 1 (V4), i.e., ${c}_{{{{{{{{\rm{sti}}}}}}}},l}^{\alpha \beta }$ = 0 when *β* = 2 or *α* = i. The synapse coupling weights for the external inputs are 5 nS. For Fig. 3, we add 1 input at ${{{{{{{{\bf{y}}}}}}}}}_{{{\rm{sti}}},1}^{{{{{{{{\rm{e}}}}}}}}1}$ = (0, 0), with ${c}_{{{\rm{sti}}},1}^{{{\rm{e}}}1}$ = 0.25. For Figs. 5 and 7, we add 2 inputs at ${{{{{{{{\bf{y}}}}}}}}}_{{{{{{{{\rm{sti}}}}}}}},1}^{{{{{{{{\rm{e}}}}}}}}1}$ = (0, 0) and ${{{{{{{{\bf{y}}}}}}}}}_{{{{{{{{\rm{sti}}}}}}}},2}^{{{{{{{{\rm{e}}}}}}}}1}$ = (−32, −32), with ${c}_{{{{{{{{\rm{sti}}}}}}}},1}^{{{{{{{{\rm{e1}}}}}}}}}={c}_{{{{{{{{\rm{sti}}}}}}}},2}^{{{{{{{{\rm{e1}}}}}}}}}$ = 0.25.

### Tracking the wave patterns

Because there is one wave pattern in each of the two areas, we use the center of mass (CoM) of the spiking activity of excitatory neurons in a whole area as a proxy of the center of the wave pattern, denoted by ${{{{{{{{\bf{y}}}}}}}}}_{{{{{{{{\rm{c}}}}}}}}}(t)=(\,{y}_{{{{{{{{\rm{c}}}}}}}},1}(t),{{{{{{{y}}}}}}}_{{{{{{{{\rm{c}}}}}}}},2}(t))$. We have

14 $${y}_{{{{{{{{\rm{c}}}}}}}},z}(t)=arg\left({\sum}_{j=1}^{{N}_{{{{{{{{\rm{e}}}}}}}}}}{n}_{j}{e}^{i\frac{{y}_{j,z}}{32}\pi }\right)\frac{32}{\pi },$$

where *z* ∈ {1, 2}, *n*_*j*_ is the number of spikes emitted by the *j*^*th*^ excitatory neuron during a short time window [*t* − *τ*/2, *t* + *τ*/2] (*τ* = 10 ms unless otherwise stated), *y*_*j*,*z*_ is the horizontal (*z* = 1) and vertical (*z* = 2) coordinate of the *j*^*th*^ excitatory neuron, *arg*( ⋅ ) denotes the argument function, and 32 is the half-width of the side of each area.

To verify that the CoM is a reliable estimate of the location of the wave pattern, we analyze the average firing rate of neurons relative to their distance from the CoM. This analysis is conducted across four different ranges of global firing rates (0–2.5 Hz, 2.5–5 Hz, 5–7.5 Hz, and > 7.5 Hz) to assess the estimate’s robustness to fluctuations in global firing rate. We find that, irrespective of the global firing rate range, neurons’ firing rates decrease as their distance from the CoM increases. This relationship between firing rate and distance closely follows a Gaussian function with an added baseline firing rate (Supplementary Fig. 15). This indicates that the CoM effectively estimates the location of the strongest firing activity in an area and remains relatively robust to fluctuations in the global firing rate. Furthermore, this analysis supports the observation that, most of the time, there is only one dominant wave pattern.

To further illustrate the robustness of our results to different wave pattern tracking methods, we compare the CoM with an alternative wave pattern detection method described in ref. ^83^, where the instantaneous firing rate across the network is modeled as a 2D Gaussian function with a baseline rate, and the parameters are estimated using maximal likelihood estimation (see Supplementary Methods 3). This spatial profile of wave patterns gives it a packet-like property. The trajectory of the pattern generated by this method closely aligns with the CoM (Supplementary Fig. 16). Given that computing the CoM is much faster than fitting a Gaussian function to the instantaneous firing rate, we employ the CoM in our primary analyses.

### MUA, LFP proxy, power spectrum, and gamma bursts

The multi-unit activity (MUA) at position **y** in each area is calculated as the average firing rate of a local group of excitatory neurons within a radius of 5 grid points (80 neurons in total) centered at **y**, computed over a sliding time window with a duration of *t*_b_. We use *t*_b_ = 10 ms for On/Off state detection and *t*_b_ = 1 ms for the power spectrum, PLV, and transfer entropy analysis. The LFP at position **y** in area *β *is defined as the weighted sum of the synaptic current received by excitatory neurons across the area^84^

15 $${{{{{{{\rm{LFP}}}}}}}}\left({{{{{{{\bf{y}}}}}}}},t,\beta \right)={\sum}_{j=1}^{{N}_{{{{{{{{\rm{e}}}}}}}}}}\left(\left\vert {I}_{j,{{{{{{{\rm{rec}}}}}}}}}^{{{{{{{{\rm{e1e\beta }}}}}}}}}\left(t\right)\right\vert +\left\vert {I}_{j,{{{{{{{\rm{rec}}}}}}}}}^{{{{{{{{\rm{e2e\beta }}}}}}}}}\left(t\right)\right\vert +\left\vert {I}_{j,{{{{{{{\rm{rec}}}}}}}}}^{{{{{{{{\rm{i\beta e\beta }}}}}}}}}\left(t\right)\right\vert \right)\exp \left(-\frac{{\left\Vert {{{{{{{{\bf{y}}}}}}}}}_{j}^{{{{{{{{\rm{e}}}}}}}}\beta }-{{{{{{{\bf{y}}}}}}}}\right\Vert }^{2}}{2{\sigma }_{{{{{{{{\rm{LFP}}}}}}}}}^{2}}\right),$$

where ${{{{{{{{\bf{y}}}}}}}}}_{j}^{{{{{{{{\rm{e}}}}}}}}\beta }$ is the coordinate of neuron ${n}_{j}^{{{{{{{{\rm{e}}}}}}}}\beta }$, $| {I}_{j,{{{{{{{\rm{rec}}}}}}}}}^{{{{{{{{\rm{e}}}}}}}}1{{{{{{{\rm{e}}}}}}}}\beta }\left(t\right)|$ and $| {I}_{j,{{{{{{{\rm{rec}}}}}}}}}^{{{{{{{{\rm{e2e}}}}}}}}\beta }\left(t\right)|$ respectively represent the absolute values of the total intra- and inter-areal excitatory current received by neuron ${n}_{j}^{{{{{{{{\rm{e}}}}}}}}\beta }$, $| {I}_{j,{{{{{{{\rm{rec}}}}}}}}}^{{{{{{{{\rm{i}}}}}}}}\beta {{{{{{{\rm{e}}}}}}}}\beta }\left(t\right)|$ is the absolute value of the total intra-areal inhibitory current received by neuron ${n}_{j}^{{{{{{{{\rm{e}}}}}}}}\beta }$ (note that the inhibitory neurons do not project inter-areal connections in our model), and *σ*_LFP_ = 7 defines the spatial scale of the weighted sum. Both MUA and LFP signals are sampled every 1 ms (1 kHz sampling rate), and are recorded at the network center **y** = (0, 0), except for in Fig. 5, where we also record the MUA at the network corner **y** = (−32, −32).

For the power spectrum in Fig. 1e, we generate 30 random realizations of the network. In each realization, we run 50-second simulations and segment them into ten 5-second epochs. The power spectrum of the MUA and LFP during each of these epochs is calculated using the Fast Fourier transform and its arrhythmic 1/*f* component is estimated using irregular-resampling auto-spectral analysis^40^. The power spectrum and 1/*f* component are averaged across epochs for each realization, and the grand-average results across realizations are presented.

For the gamma bursts in MUA (Fig. 1c, d and Supplementary Fig. 4a,b) and LFP (Supplementary Fig. 4c, d), we generate 30 random realizations of the network. For each realization, we apply Complex Morlet wavelet transform to 200 s of MUA and LFP time series to obtain the instantaneous amplitude time series for each frequency spanning the gamma band (30–80 Hz, sampled at 2 Hz intervals). The amplitude time series is then smoothed by a Gaussian kernel with SD = 3 ms. To detect the gamma bursts, we threshold the time series for each frequency at the 85th percentile of the instantaneous amplitude pooled from all times and all gamma-band frequencies in each network realization. In Supplementary Fig. 4, the amplitude is converted to power.

### Spike frequency adaptation and cued top-down attention

For modeling the effect of the cue, we decrease the spike-frequency adaptation ${{\Delta }}{g}_{{{{{{{{\rm{k}}}}}}}}}^{{{{{{{{\rm{e2}}}}}}}}}$ of excitatory neurons near the cued region in FEF. The new value of ${{\Delta }}{g}_{{{{{{{{\rm{k}}}}}}}}}^{{{{{{{{\rm{e2}}}}}}}}}$ at coordinate ***y***_***j***_ under the cued condition is given by

16 $${{\Delta }}{g}_{{{{{{{{\rm{k}}}}}}}}}^{{{{{{{{\rm{e2}}}}}}}}}\left({{{{{{{{\bf{y}}}}}}}}}_{j}\right)={{\Delta }}{g}_{{{{{{{{\rm{k,base}}}}}}}}}^{{{{{{{{\rm{e2}}}}}}}}}-{{\Delta }}{g}_{{{{{{{{\rm{k,modu}}}}}}}}}^{{{{{{{{\rm{e2}}}}}}}}}\frac{{f}_{{{{{{{{\rm{att}}}}}}}}}\left({{{{{{{{\bf{y}}}}}}}}}_{j},{{{{{{{{\bf{y}}}}}}}}}_{{{{{{{{\rm{att}}}}}}}}},{R}_{{{{{{{{\rm{att}}}}}}}}},{\sigma }_{{{{{{{{\rm{att}}}}}}}}}\right)}{{f}_{{{{{{{{\rm{att}}}}}}}}}\left({{{{{{{{\bf{y}}}}}}}}}_{{{{{{{{\rm{att}}}}}}}}},{{{{{{{{\bf{y}}}}}}}}}_{{{{{{{{\rm{att}}}}}}}}},{R}_{{{{{{{{\rm{att}}}}}}}}},{\sigma }_{{{{{{{{\rm{att}}}}}}}}}\right)},$$

where

17 $$\begin{array}{rlr}&{f}_{att}\left({{{{{{{{\bf{y}}}}}}}}}_{j},{{{{{{{{\bf{y}}}}}}}}}_{{{{{{{{\rm{att}}}}}}}}},{R}_{{{{{{{{\rm{att}}}}}}}}},{\sigma }_{{{{{{{{\rm{att}}}}}}}}}\right)=&\\ &\left(\frac{1}{1+\exp \left(-\frac{\left\Vert {{{{{{{{\bf{y}}}}}}}}}_{j}-{{{{{{{{\bf{y}}}}}}}}}_{{{{{{{{\rm{att}}}}}}}}}\right\Vert +{R}_{{{{{{{{\rm{att}}}}}}}}}}{{\sigma }_{{{{{{{{\rm{att}}}}}}}}}}\right)}\right)\left(1-\frac{1}{1+\exp \left(-\frac{\left\Vert {{{{{{{{\bf{y}}}}}}}}}_{j}-{{{{{{{{\bf{y}}}}}}}}}_{{{{{{{{\rm{att}}}}}}}}}\right\Vert -{R}_{{{{{{{{\rm{att}}}}}}}}}}{{\sigma }_{{{{{{{{\rm{att}}}}}}}}}}\right)}\right),\end{array}$$

${{\Delta }}{g}_{{{{{{{{\rm{k,base}}}}}}}}}^{{{{{{{{\rm{e2}}}}}}}}}$ = 6.5 nS is the baseline value, **y**_att_ = (0, 0) is the center of spike-frequency modulation profile *f*_*att*_. We refer to **y**_att_ as the cued location. *R*_att_ = 8.2 and *σ*_att_ = 2.2 define the range and shape of the modulation profile, respectively. The maximum reduction in the adaptation is ${{\Delta }}{g}_{{{{{{{{\rm{k,modu}}}}}}}}}^{{{{{{{{\rm{e2}}}}}}}}}$ = 6 nS at **y**_att_, leading to the minimum adaptation across FEF equal to 0.5 nS.

### Phase-amplitude coupling

To quantify the phase-amplitude coupling between different frequency components, we calculate the modulation index (MI)^42^. We first band-pass the raw LFP at the phase-frequency band (LFP_p_) and amplitude-frequency band (LFP_a_), and then extract the instantaneous phase of LFP_p_ and instantaneous amplitude of LFP_a_ using the Hilbert transform. The phase time series LFP_p_ are divided into *N* = 20 phase intervals, and for each phase interval, we calculate the average amplitude of LFP_a_ to obtain the distribution of the amplitude with respect to the phase, given by

18 $$p({{\Phi}}_j) = \frac{{A}_{{{\Phi}}_j}}{\mathop{\sum}\nolimits_{j=1}^{N}{A}_{{{\Phi}}_j}},$$

where ${A}_{{{{\Phi }}}_{j}}$ is the average amplitude for phase interval Φ_*j*_. The MI is a measure of divergence of *p*(Φ_*j*_) from the uniform distribution, given by

19 $${{{{{{{\rm{MI}}}}}}}}=1-\frac{-\mathop{\sum }\nolimits_{j = 1}^{N}p({{{\Phi }}}_{j})\log (p({{{\Phi }}}_{j}))}{\log (N)}.$$

To eliminate the possibility of MI being caused by chance, we randomly shuffle the amplitude time series LFP_a_ and calculate the MI between the shuffled LFP_a_ and original LFP_p_; we refer to this MI calculated with shuffled LFP as MI_s_. We calculate the MI_s_ for 200 random shuffles and then subtract the average MI_s_ from the raw MI to obtain the final MI. For Fig. 1f and Fig. 6b, we generate 30 random realizations of the network. In each network realization, we calculate the MI based on 50 s of LFP for each condition [spontaneous (uncued) and cued]. The average MI across realizations is presented.

### On and Off states detection

The local On and Off states are classified based on the MUA of a local group of 80 excitatory neurons in a circular region with a radius of 5 grid points. In Fig. 1, 2, 3, 5, and 7, the group of neurons considered is at the center of networks. For Fig. 5, we also consider the neuron group located at the network corner (i.e., the location of the corner input). The MUA is computed as the mean firing rate of these neurons in the group using 10 ms bins sampled at 1 kHz. The MUA is smoothed by using the Savitzky-Golay filter before detecting the abrupt change points in the MUA using the ‘findchangepts’ function in MATLAB (MATLAB R2019b, MathWorks, Natick, MA). We refer to the smoothed MUA as ${\eta }_{1}\left(t\right)$ and the time of the *n*^*th*^ change point detected by the ‘findchangepts’ as $p{t}_{1}\left(n\right)$. The $p{t}_{1}\left(n\right)$ is a preliminary segmentation of the MUA into high and low activity phases. To improve the On-Off classification results, we derive a new signal ${\eta }_{2}\left(t\right)$ from ${\eta }_{1}\left(t\right)$, where ${\eta }_{2}\left(t\right)=\frac{1}{p{t}_{1}\left(n+1\right)-p{t}_{1}\left(n\right)}\int\nolimits_{p{t}_{1}\left(n\right)}^{p{t}_{1}\left(n+1\right)}{\eta }_{1}\left(\tau \right)d\tau$, for $t\in [p{t}_{1}\left(n\right),p{t}_{1}\left(n+1\right))$. We then segment the ${\eta }_{2}\left(t\right)$ into On and Off states by comparing ${\eta }_{2}\left(t\right)$ at each time point with a threshold *θ*_*η*_, and define a new signal ${\eta }_{01}\left(t\right)$, with ${\eta }_{01}\left(t\right)$ = 1 if ${\eta }_{2}\left(t\right)\ge {\theta }_{\eta }$ (On states) and ${\eta }_{01}\left(t\right)$ = 0 if ${\eta }_{2}\left(t\right) < {\theta }_{\eta }$ (Off states). The On-Off transition points in ${\eta }_{01}\left(t\right)$ is referred to as $p{t}_{2}\left(n\right)$. Similar to ${\eta }_{2}\left(t\right)$, using $p{t}_{2}\left(n\right)$ we can define a signal ${\eta }_{3}\left(t\right)=\frac{1}{p{t}_{2}\left(n+1\right)-p{t}_{2}\left(n\right)}\int\nolimits_{p{t}_{2}\left(n\right)}^{p{t}_{2}\left(n+1\right)}{\eta }_{1}\left(\tau \right)d\tau$, for $t\in [p{t}_{2}\left(n\right),p{t}_{2}\left(n+1\right))$. To find the appropriate value of *θ*_*η*_ we try a range of *θ*_*η*_ and find the sum of squared error ${{{{{{{\rm{SSE}}}}}}}}={\sum }_{t}{\left({\eta }_{3}\left(t\right)-{\eta }_{1}\left(t\right)\right)}^{2}$ for each *θ*_*η*_. The *θ*_*η*_ giving the minimum SSE is chosen to be the threshold for the On-Off states classification, and *t* is at On state if ${\eta }_{2}\left(t\right)\ge {\theta }_{\eta }$, and Off state if ${\eta }_{2}\left(t\right) < {\theta }_{\eta }$. We determine the best *θ*_*η*_ separately for each realization of the network.

For Fig. 2b, c, e, we generate 30 random realizations of the network, and for Fig. 2d, we generate 60 random realizations of the network. In each realization, the On-Off states detection is performed on 200 s of MUA data. For Fig. 3c–f, we generate 60 random realizations of the network. In each realization, we run 30 trials (10 s for each trial), and the On-Off states detection is performed on the combined MUA across trials. For Fig. 5c, e, f and Fig. 7c–e, g, we generate 30 random realizations of the network. In each realization, we run 20 trials (10  s for each trial) for each condition (uncued/cued), and the On-Off states detection is performed on the combined MUA across trials of the same condition. For Fig. 5d and Fig. 7f, we generate 60 random realizations of the network. In each realization, we run 30 trials (10 s for each trial) for each condition (uncued/cued) and the On-Off states detection is performed on the combined MUA across trials of the same condition. For all stimulus-evoked trials, the initial 200 ms of MUA on each trial are excluded from the analysis.

### Coordination of simultaneous-On states by theta oscillations

To examine the relationship between simultaneous-On (S-On) states and theta oscillations of local MUA (Figs. 3f and 5f), we first band-pass the MUA at the center of area 1 into the theta band (3–6 Hz) using an 8^th^ order forward-backward Butterworth band-pass filter, and then obtain the theta phase using Hilbert transform. The theta phase at the middle time point of each S-On state occurring at the network center (Figs. 3f and 5f) and corner (Fig. 5f) is recorded, and the distribution of the theta phase pooled across network realizations is shown.

### Phase synchronization

To measure the phase synchronization between the MUA in area 1 (V4) and area 2 (FEF) in the gamma band, we first use an 8^th^ order forward-backward Butterworth band-pass filter to filter the MUA in narrow band ${F}_{{{{{{{{\rm{b}}}}}}}}}=\left[{f}_{{{{{{{{\rm{c}}}}}}}}}-5{{{{{{{\rm{Hz}}}}}}}},{f}_{{{{{{{{\rm{c}}}}}}}}}+5{{{{{{{\rm{Hz}}}}}}}}\right]$, with *f*_c_ ± 5Hz being the -3dB cutoff frequency and *f*_c_ = 30, 40, …, 120Hz. We then apply the Hilbert transform to the two filtered MUAs to extract the time series of the instantaneous phase ${\phi }_{{f}_{{{{{{{{\rm{c}}}}}}}}}}^{i}\left(t\right)$ of the MUA in each area (*i* = 1 for area 1/V4 and *i* = 2 for area 2/FEF) for each *f*_c_. The phase locking value (PLV) at *f*_c_ is defined as the mean resultant length of relative phase

20 $${{{{{{{\rm{PLV}}}}}}}}=\left\vert {\langle {e}^{i[{\phi }_{{f}_{{{{{{{{\rm{c}}}}}}}}}}^{1}(t)-{\phi }_{{f}_{{{{{{{{\rm{c}}}}}}}}}}^{2}(t)]}\rangle }_{t}\right\vert ,$$

where 〈⋅〉_*t*_ denotes the averaging across time. We measure the PLV during S-On states (averaging across periods of S-On states) and S-Off states (averaging across periods of S-Off states) separately. To remove spurious phase locking due to band-pass filtering, we randomly shuffle MUA across time (white noise) 200 times and calculate the PLV between the shuffled MUA for each shuffling. Finally, we subtract the average PLV of the shuffled MUA from the PLV of the original MUA.

The simulation setup has been specified in the ‘On and Off states detection’. We perform PLV analysis for each state (S-On/S-Off) and each condition (spontaneous, uncued/cued) in each network realization and then average the PLV across realizations.

### Communication subspace

We analyze the communication subspace between two groups of excitatory neurons in a circular region with a radius of 5 grid points at the center of area 1 (V4) and area 2 (FEF) (the same group of 80 neurons used for classifying On/Off states). We first record the number of spikes in non-overlap 20 ms windows (single unit activity, SUA) for each of the 80 excitatory neurons in each group. The SUA data of area 1 (V4) and area 2 (FEF) constitute two matrices, *X*_*s*_ and *Y*_*s*_, respectively. Each matrix is a *n* × *p* matrix, where *p* = 80 represents the number of neurons and *n* is the number of data points recorded over time. Next, we calculate the average SUA of each neuron during On states (${\overline{X}}_{s}^{{{{{{{{\rm{On}}}}}}}}}$ and ${\overline{Y}}_{s}^{{{{{{{{\rm{On}}}}}}}}}$, 1 × *p* matrix) and Off states (${\overline{X}}_{s}^{{{{{{{{\rm{Off}}}}}}}}}$ and ${\overline{Y}}_{s}^{{{{{{{{\rm{Off}}}}}}}}}$), respectively. We then compute the SUA fluctuations by subtracting the average SUA from the raw SUA according to the state in which each data point resides. For instance, if the first row of *X*_*s*_ is at the On state, then the ${\overline{X}}_{s}^{{{{{{{{\rm{On}}}}}}}}}$ is subtracted from it. The fluctuations of SUA are referred to as *X*^On^, *Y*^On^ (*n*^On^ × *p* matrix, *n*^On^ is the number of data points of On states), *X*^Off^, and *Y*^Off^ (*n*^Off^ × *p* matrix, *n*^Off^ is the number of data points of Off states). To quantify the inter-areal interaction, we relate the ongoing fluctuations of SUA between two neuron groups of neurons in two areas through linear regression

21 $$Y=XB,$$

where (*X*, *Y*) ∈ {(*X*^On^, *Y*^On^), (*X*^Off^, *Y*^Off^)}, and *B* is a *p* × *p* coefficient matrix. For the full model, we use the ridge regression where $B={B}_{rdg}={\left({X}^{T}X+\lambda I\right)}^{-1}X^{T}Y$ and the predicted *Y* is *Y*_*rdg*_ = *XB*_*rdg*_. Here *I* is a *p* × *p* identity matrix and *λ* is a scalar. We determine the appropriate value of *λ* using the 10-fold cross-validation as in ref. ^4^. Briefly, in each fold, we measure the prediction performance which is defined as the normalized squared error ${{{{{{{\rm{NSE}}}}}}}}=\frac{\langle {\left(Y-{Y}_{rdg}\right)}^{2}\rangle }{\langle {\left(Y-\overline{Y}\right)}^{2}\rangle }$ (the mean squared error normalized by the variance of *Y*). The averaging operation 〈 ⋅ 〉 for NSE is applied across all target neurons and data points. *λ* is chosen as the largest *λ* for which the mean prediction performance across folds is within one SEM (measured across folds) of the best performance across different *λ* values.

To unravel the low-dimensional nature of the communication, we perform a reduced-rank regression (RRR), similar to that in ref. ^4^, where *B* = *B*_*RRR*_ = *B*_*rdg*_*VV*^*T*^. Here *V* is a *p* × *m* matrix with the column vectors as the top *m* principal component of *Y*_*rdg*_. We calculate the performance for *m* = 1–10, respectively, using 10-fold cross-validation.

The simulation setup has been specified in the ‘On and Off states detection’. We perform the subspace analysis for each state (S-On/S-Off) and each condition (spontaneous, uncued/cued) in each network realization, and then average the prediction performance across realizations.

### Transfer entropy

To quantify the information transferred from area 1 (V4) to area 2 (FEF) during different states, we measure the transfer entropy (TE) between the MUA in the two areas during S-On and S-Off states, respectively. MUA is defined as the average firing rate over 1 ms time windows of a local group of 80 excitatory neurons within 5 grid points from the network center. The sampling interval for MUA is 1 ms (1kHz sampling rate). TE measures the reduction in the uncertainty (entropy) of the future value of the MUA in area 2 (target) by knowing the past value of the MUA in area 1 (source), which is given by

22 $${{{{{{{\rm{TE}}}}}}}}=H({T}_{t+1}| {{{{{{{{\boldsymbol{T}}}}}}}}}_{t}^{k})-H({T}_{t+1}| {{{{{{{{\boldsymbol{T}}}}}}}}}_{t}^{k},{{{{{{{{\boldsymbol{S}}}}}}}}}_{t+1-u}^{l}),$$

where *H*( ⋅ ) denotes the entropy of a random variable, *T*_*t*+1_ is the MUA in area 2 at time *t* + 1, ${{{{{{{{\boldsymbol{T}}}}}}}}}_{t}^{k}=({T}_{t-k+1},{T}_{t-k+2},\ldots ,{T}_{t})$ denotes the past *k* values of the MUA in area 2 at *t*, and ${{{{{{{{\boldsymbol{S}}}}}}}}}_{t+1-u}^{l}=({S}_{t-l+2-u},{S}_{t-l+3-u},\ldots ,{S}_{t+1-u})$ denotes the past *l* values of area 1 MUA with a source-target time delay *u*. These values are calculated within the epochs of S-On states to compute TE during the S-On states and within the epochs of S-Off states to compute the TE during the S-Off states. TE can be expressed in the form of probability density functions of *T*_*t*+1_, ${{{{{{{{\boldsymbol{T}}}}}}}}}_{t}^{k}$, and ${{{{{{{{\boldsymbol{S}}}}}}}}}_{t+1-u}^{l}$ as

23 $${{{{{{{\rm{TE}}}}}}}}=\int_{{T}_{t+1},{{{{{{{{\boldsymbol{T}}}}}}}}}_{t}^{k},{{{{{{{{\boldsymbol{S}}}}}}}}}_{t+1-u}^{l}}p({T}_{t+1},{{{{{{{{\boldsymbol{T}}}}}}}}}_{t}^{k},{{{{{{{{\boldsymbol{S}}}}}}}}}_{t+1-u}^{l}){\log }_{e}\left(\frac{p({T}_{t+1}| {{{{{{{{\boldsymbol{T}}}}}}}}}_{t}^{k},{{{{{{{{\boldsymbol{S}}}}}}}}}_{t+1-u}^{l})}{p({T}_{t+1}| {{{{{{{{\boldsymbol{T}}}}}}}}}_{t}^{k})}\right)d{T}_{t+1}\,d{{{{{{{{\boldsymbol{T}}}}}}}}}_{t}^{k}\,d{{{{{{{{\boldsymbol{S}}}}}}}}}_{t+1-u}^{l}.$$

We compute the TE for different time delays *u* ranging from 1 to 20 ms. The TE is estimated using the Kraskov Algorithm^85^. We determine the optimal history sizes, *k* and *l*, from 1 to 8 ms using the Ragwitz criterion^86^. All TE analyses are performed using the information-theoretic toolkit JIDT^87^. The simulation setup has been specified in the ‘On and Off states detection’. We conduct the TE analysis for each state (S-On/S-Off) and each condition (spontaneous, uncued/cued) in each network realization, and then average the TE across realizations.

### Fano factor and noise correlation in the spiking model

To show that our circuit model exhibits variable firing activity, we calculate the coefficient of variation of interspike interval and the Fano factor of spike counts for all excitatory neurons in individual network realizations (*n* = 60 networks). 15 s of spontaneous activities are analyzed for each network. The Fano factor is computed as the ratio of the spike count variance to the mean spike count, with the spike count measured over non-overlapping time windows (window length = 50 ms). To analyze the influence of feedforward and feedback inputs on neural variability, we compare the Fano factor of spike counts before and after disconnecting interareal connections. The Fano factor and coefficient of variation of individual neurons are averaged across neurons for each network and then grand averaged across networks.

To compute the time-resolved Fano factor in Fig. 7h and noise correlation in Fig. 7i, we generate 60 random realizations of the network; in each realization, we conduct 50 trials for both the uncued and cued conditions, respectively (3000 trials for each attention condition). During each trial, we present two external inputs to the excitatory neuron group in V4 for 600 ms. These inputs are positioned at the center and corner of V4, i.e., ${{{{{{{{\bf{y}}}}}}}}}_{{{{{{{{\rm{sti}}}}}}}},1}^{{{{{{{{\rm{e}}}}}}}}1}$ **=**(0, 0) and ${{{{{{{{\bf{y}}}}}}}}}_{{{{{{{{\rm{sti}}}}}}}},2}^{{{{{{{{\rm{e}}}}}}}}1}$ **=**(−32, −32), with an input contrast ${c}_{{{{{{{{\rm{sti}}}}}}}},1}^{{{{{{{{\rm{e1}}}}}}}}}$ = ${c}_{{{{{{{{\rm{sti}}}}}}}},2}^{{{{{{{{\rm{e1}}}}}}}}}$ = 0.25. The interval between two successive trials follow a random distribution ranging from 800 to 1500 ms. For the cued condition, the reduction in the local spike frequency adaptation at FEF begins 2000 ms before the first trial and persists throughout the remaining trials.

The time-resolved Fano factor is computed using the mean-matching method^32^. Briefly, in each network realization, we select a group of 80 excitatory neurons whose distance from the center of V4 (i.e., the center input location) is <5 grid points. For each attention condition (uncued/cued), we calculate the mean and variance of spike counts across trials for each neuron in that group using a counting window of 50 ms (sampled at 10 ms intervals across the time course of the trial). For each time point, we randomly sample a subset of neurons in a way that the mean spike counts of the subset neurons at each time point and attention condition are approximately equal. Subsequently, the Fano factor at each time point is computed as the slope of the mean-variance relationship for the mean-matched subset of neurons using linear regression. This mean-matched random neuron subset sampling is repeated 100 times for each time point, and the resulting Fano factors are averaged to obtain the final mean-matched Fano factor. This analysis is conducted for each network realization, and the average Fano factor across realizations is presented. To compute the noise correlation, we select the same neuron group as in the Fano factor analysis. Noise correlation is determined as the Pearson correlation coefficient between the spike counts of two neurons across trials. We employ a spike counting window of 50 ms, sampled at 10 ms intervals throughout the trial. For each time point, we calculate the noise correlation for every pair of neurons within that group. These correlations are then averaged across neuron pairs to obtain the time-resolved noise correlation for each attention condition within each network realization. The grand average noise correlation across network realizations is presented.

### The mathematical model accounting for the attention modulation of the neural variability and correlation

We consider a 2D plane with dimensions [−32, 32] × [−32, 32], matching the size of each area of the spiking model, and we assume there are 4096 neurons uniformly distributed on this plane. The potential at position ***x*** is

24 $$\rho ({{{{{{{\bf{x}}}}}}}})=\mathop{\sum}_{i}{\rho }_{i}({{{{{{{\bf{x}}}}}}}})$$

where

25 $${\rho }_{i}({{{{{{{\bf{x}}}}}}}})=({D}_{{{{{{{{\rm{s}}}}}}}},i}+{D}_{{{{{{{{\rm{a}}}}}}}},i})\left(\frac{{\parallel {{{{{{{\bf{x}}}}}}}}-{{{{{{{{\bf{x}}}}}}}}}_{{{{{{{{\rm{p}}}}}}}},i}\parallel }^{2}}{{{\sigma }_{{{{{{{{\rm{p}}}}}}}}}}^{2}}-1\right),\,\,{{\mbox{if}}}\,\,\parallel {{{{{{{\bf{x}}}}}}}}-{{{{{{{{\bf{x}}}}}}}}}_{{{{{{{{\rm{p}}}}}}}},i}\parallel < {\sigma }_{{{{{{{{\rm{p}}}}}}}}},$$

or

26 $${\rho }_{i}({{{{{{{\bf{x}}}}}}}})=0,\,\,{{\mbox{if}}}\,\,\parallel {{{{{{{\bf{x}}}}}}}}-{{{{{{{{\bf{x}}}}}}}}}_{{{{{{{{\rm{p}}}}}}}},i}\parallel \ge {\sigma }_{{{{{{{{\rm{p}}}}}}}}},$$

where **x**_p,1_ = (0, 0) and **x**_p,2_ = (-32, -32) are the coordinates of the potential wells, *σ*_p_ =15 is the width of the potential wells, *D*_s,1_ = *D*_s,2_ = 50 is the depth of the potential well contributed by the external inputs, *D*_a,2_ = 0 and *D*_a,1_ varies from 0 to 50. The drift term *b*(**x**_*t*_) in the stochastic differential equations Eq. (1) and (2) is defined as^88^:

27 $$b({{{{{{{{\bf{x}}}}}}}}}_{t})=-\nabla \rho ({{{{{{{{\bf{x}}}}}}}}}_{t})\frac{{{\Gamma }}(\alpha -1)}{{{{\Gamma }}(\alpha /2)}^{2}},$$

where **x**_*t*_ is the coordinate of the random walker, ∇ *ρ*(**x**_*t*_) denotes the gradient of the potential landscape, Γ( ⋅ ) is the Gamma function, and the tail index *α* = 1.2. The instantaneous firing rate *r*(**x**) of a neuron at **x** is

28 $$r({{{{{{{\bf{x}}}}}}}})={A}_{r}{e}^{-\frac{{\parallel {{{{{{{\bf{x}}}}}}}}-{{{{{{{{\bf{x}}}}}}}}}_{t}\parallel }^{2}}{2{{\sigma }_{r}}^{2}}}+{A}_{0},$$

where *A*_*r*_ = 20 Hz is the amplitude of the Gaussian bump, *σ*_*r*_ = 12 is the width of the Gaussian bump, and *A*_0_ is the baseline firing rate. To simulate the attention-related increase in the firing rate, we adjust *A*_0_ based on *D*_*a*,1_:

29 $${A}_{0}={A}_{{{{{{{{\rm{b}}}}}}}}}-0.2{D}_{{{{{{{{\rm{a}}}}}}}},1}\left(\frac{{\parallel {{{{{{{\bf{x}}}}}}}}-{{{{{{{{\bf{x}}}}}}}}}_{{{{{{{{\rm{p}}}}}}}},1}\parallel }^{2}}{{{\sigma }_{{{{{{{{\rm{p}}}}}}}}}}^{2}}-1\right),\,\,{{\mbox{if}}}\,\,\parallel {{{{{{{\bf{x}}}}}}}}-{{{{{{{{\bf{x}}}}}}}}}_{{{{{{{{\rm{p}}}}}}}},1}\parallel < {\sigma }_{{{{{{{{\rm{p}}}}}}}}},$$

or

30 $${A}_{0}={A}_{{{{{{{{\rm{b}}}}}}}}},\,\,{{\mbox{if}}}\,\,\parallel {{{{{{{\bf{x}}}}}}}}-{{{{{{{{\bf{x}}}}}}}}}_{{{{{{{{\rm{p}}}}}}}},1}\parallel \ge {\sigma }_{{{{{{{{\rm{p}}}}}}}}},$$

where *A*_*b*_ = 3 Hz is the baseline rate when the attention is absent.

The On states at the center of this mathematical model (Fig. 8b) is defined as the period when the random walker **x**_*t*_ is less than 10 grid points from the center. To compute the Fano factor (Fig. 8c) and noise correlation (Fig. 8d) in the mathematical model, we run 20 trials (200 s for each trial) for each potential well depth *D*_a,1_ (from *D*_a,1_ = 0 to *D*_a,1_ = 50). The initial position of the random walker is randomized for each trial. To compute the Fano factor, we select 80 neurons within 5 grid points from the center of the model and calculate the mean and variance of spike counts over non-overlapping time windows across time (window length = 55 ms) for each neuron on each trial. The Fano factor for each neuron is obtained by dividing the spike count variance by the mean count. The Fano factor of each neuron is averaged for each trial and then grand averaged across trials to produce the results in Fig. 8c. To compute the noise correlation, we choose the same group of neurons as that in the Fano factor analysis. Noise correlation is calculated as the Pearson correlation coefficient of spike counts for neuron pairs over non-overlapping time windows across time (window length = 55 ms). We compute the noise correlation for all neuron pairs in that group for each trial and then average them to get the mean noise correlation for each trial, which is then grand averaged across trials (Fig. 8d).

### Statistics and reproducibility

For our study of spiking circuit model, we randomly generate network realizations by randomizing the initial membrane potential and the synaptic connectivity. Analyses of On/Off states duration, On/Off states firing rate, phase synchronization, communication subspace, transfer entropy, and neural variability and correlation are performed on each random network realization. The results from individual realizations are then averaged, and differences between conditions (i.e., On/Off, uncued/cued) are assessed using two-sided and one-sided paired t-test. The standard error of the mean (SEM) and standard deviation (SD) are calculated across network realizations. The number of realizations (sample size) is stated in corresponding figure captions or main text, and the simulation setup is specified in the Methods section above. For the mathematical model, we run random trials by randomizing the initial position of the random walker. Differences between groups are assessed using two-sided unpaired t-test. The number of random trials (sample size) is stated in corresponding figure captions. P-values < 0.05 are considered to be statistically significant. For our study of experimental neural data, we present both individual data from a representative mouse and cross-individual data from a selected set of nine mice (chosen according to criteria specified in Supplementary Methods 2). We report median values and interquartile ranges for prediction performance across subjects, as described in the figure captions.

### Reporting summary

Further information on research design is available in the Nature Portfolio Reporting Summary linked to this article.

### Supplementary information

Supplementary Information

Description of Additional Supplementary Files

Supplementary Data 1

Supplementary Movie 1

Supplementary Movie 2

Reporting Summary

## Data Availability

Data used in this study will be made available upon request by contacting the lead contact, Pulin Gong (pulin.gong@sydney.edu.au). The source data for the figures is provided in Supplementary Data 1.

## Code availability

Code for simulations and analysis of the spiking neural circuit model and the mathematical model is available at^89^ 10.5281/zenodo.10959801(https://github.com/BrainDynamicsUSYD/Distributed_and_dynamical_communication). Code for our analyses of experimental recordings are openly available, on request.
