# Supplementary material for: Distributed and dynamical communication: a mechanism for flexible cortico-cortical interactions and its functional roles in visual attention
Source: Commun Biol. 2024 May 8;7:550. doi: 10.1038/s42003-024-06228-z (PMC11078951; doi:10.1038/s42003-024-06228-z)
Supplement: Supplementary file 1 — Supplementary Information [file 42003_2024_6228_MOESM1_ESM.pdf]

Supplementary Information

Distributed and dynamical communication: A mechanism for flexible cortico-cortical interactions and its functional roles in visual attention

Shencong Ni[1], Brendan Harris[1], Pulin Gong[1*]

1 School of Physics, University of Sydney, NSW 2006, Australia

* *pulin.gong@sydney.edu.au*

Supplementary Methods 1: Theoretical analysis of theta oscillations

To develop a theoretical understanding of how adaptation induces theta oscillations, we consider a neural field model[1], which captures some features of our spiking neural circuit model, including neural adaptation in the form of spike frequency adaptation, distance-dependent synaptic coupling, and the emergence of localized activity patterns. Here we briefly describe this model and extend the mathematical analysis in Ref. [1] to derive the relation between the frequency of pattern oscillations and adaptation strength. The model is described by the following equations:

$$\tau\frac{\partial u}{\partial t} = -u + w * f\left(u\right) - k\varrho + I, \tag{1}$$

$$\tau_{\mathrm{A}}\frac{\partial \varrho}{\partial t} = -\varrho + u, \tag{2}$$

where $*$ denotes the convolution, $u\left(\mathbf{r},t\right)$ is the average activity (e.g., membrane potential) of a local group of neurons at position $\mathbf{r} = (r,\, \theta)$, $\varrho\left(\mathbf{r},t\right)$ provides negative feedback to u, behaving like spike frequency adaptation, with its strength controlled by k. τ=10 ms and τ_{A}=333.3 ms are the time scale of u and ϱ, respectively. $I = I_{\mathrm{p}}e^{-r^2/\sigma^2}$ is the external input, with I_{p}=1 and σ=5.2. f is the Heavyside activation function with $f\left(u\right) = H\left(u - \vartheta\right)$, where ϑ=0.15 is the activation threshold [$f(u) = 1$ for $u \geq \vartheta$, otherwise $f(u) = 0$]. w is the *Mexican hat* coupling weight function given by

$$w(r) = \frac{a_{\mathrm{e}}}{\sigma_{\mathrm{e}}^2}w_{\mathrm{K}}(r/\sigma_{\mathrm{e}}) - \frac{a_{\mathrm{i}}}{\sigma_{\mathrm{i}}^2}w_{\mathrm{K}}(r/\sigma_{\mathrm{i}}), \tag{3}$$

where $a_{\mathrm{e}} = 1$, $\sigma_{\mathrm{e}} = 1$, $a_{\mathrm{i}} = 1.4$, $\sigma_{\mathrm{i}} = 1.8$, $w_{\mathrm{K}}(r) = (2/3\pi)[K_0(r) - K_0(2r)]$ and K_0 is the modified Bessel function of the second kind. The synaptic input to $u\left(\mathbf{r},t\right)$ is computed as the convolution

$$w*f\left(u\right) = \int_{R^2} w\left(\|\mathbf{r}-\mathbf{r}^{'}\|\right) f\left(u\left(\mathbf{r}^{'},t\right)\right) d\mathbf{r}^{'}. \tag{4}$$

At the stationary state, we expect a radial symmetric profile of $u\left(\mathbf{r},t\right) = U\left(r\right)$ and $\varrho\left(\mathbf{r},t\right) = Q\left(r\right)$. The radius of the stationary activity pattern is denoted as a with $U\left(a\right) = \vartheta$, $0 < U\left(r\right) < \vartheta$ for $r > a$, and $U\left(r\right) > \vartheta$ for $0 < r < a$. The profile of $U\left(r\right)$ can be found by noting $U\left(r\right) = Q\left(r\right)$, giving rise to

$$\left(1 + k\right)U\left(r\right) = M\left(a, r\right) + I\left(r\right), \tag{5}$$

where

$$M(a, r) = \int_0^{2\pi}\int_0^a w(\|\mathbf{r} - \mathbf{r}^{'}\|)r^{'}dr^{'}d\theta. \tag{6}$$

At the boundary a we have

$$(1 + k)\vartheta = M(a, a) + I(a). \tag{7}$$

This stationary pattern corresponds to a fixed-point solution of a dynamical system, and its stability can be determined by examining the eigenvalues of the system linearized around this fixed point. The eigenvalue λ for each Fourier mode n satisfies

$$\tau\lambda + 1 + \frac{k}{\tau_{\mathrm{A}}\lambda + 1} = \frac{\mu_n\left(a\right)}{\left|U^{'}\left(a\right)\right|}, \tag{8}$$

where

$$\mu_n(a) = 2a \int_0^{\pi} w(2asin\phi)\, cos(2n\phi)\, d\phi. \tag{9}$$

By solving Equation (8) we have

$$\lambda_n^{\pm} = \frac{1}{2\tau}\left[-\Lambda_n \pm \sqrt{\Lambda_n^2 - 4\epsilon(1+k)(1-\Gamma_n)}\right], \tag{10}$$

where

$$\Lambda_n = 1 + \epsilon - \Gamma_n(1+k), \tag{11}$$

$$\Gamma_n = \frac{\mu_n(a)}{(1+k)|U'(a)|}, \tag{12}$$

$$\epsilon = \frac{\tau}{\tau_A}. \tag{13}$$

The stability of a particular mode n is determined by the sign of the real part of eigenvalues $\lambda_n^{\pm}$; a negative real part indicates stability, while a positive real part means instability. When the condition $k > \epsilon$ holds, the real part of $\lambda_n^{\pm}$ is negative if $\Lambda_n > 0$, which means mode n is stable. When $\Lambda_n = 0$, $\lambda_n^{\pm} = \pm i\sqrt{\epsilon(k-\epsilon)}/\tau = \pm i\sqrt{[k - (\tau/\tau_A)]/(\tau\tau_A)}$; thus, the real part of $\lambda_n^{\pm} = 0$ and the imaginary part of $\lambda_n^{\pm} \neq 0$, indicating the onset of oscillatory patterns known as 'breather' (Hopf bifurcation). The imaginary part of $\lambda_n^{\pm}$ corresponds to the critical Hopf frequency.

Folias & Bressloff[1] has illustrated the dependency of the stability of the response to the external input on the input strength I_p. In our study, we focus on the effect of the adaptation strength k on stability and oscillation frequency. According to Equation (7), k influences the radius of the stationary activity pattern a (Supplementary Figure 5c), which in turn affects the eigenvalues λ_n and the pattern stability through Equation (8). Our analysis reveals that the stationary pattern is stable [real($\lambda_n^{\pm}$) < 0] when the adaptation strength k is weak (Supplementary Figure 5a). As k increases, some Fourier modes start to destabilize through Hopf bifurcation [real($\lambda_n^{\pm}$) > 0]. For the model parameters considered here, mode $n = 3$ destabilizes first, followed by $n = 4$, $n = 2$, $n = 1$, and $n = 0$ (Supplementary Figure 5a). The critical Hopf frequency $\omega_c = \sqrt{[k - (\tau/\tau_A)]/(\tau\tau_A)}$ increases with k (as observed at the tangent points of the colored lines intersecting the black dashed line in Supplementary Figure 5b). By numerical simulations, we find that the oscillation frequency of the breather remains close to the Hopf critical frequency even when far from the bifurcation point (Supplementary Figure 5d). Furthermore, the frequency of the breather increases with k. This adaptation-dependent oscillation frequency is consistent with our spiking neural circuit model, in which theta frequency increases with spike frequency adaptation (Supplementary Figure 6).

Supplementary Methods 2: Experimental data

To validate our modeling predictions against experimental recordings of neural activity, we analyze the Allen Neuropixels visual coding dataset. These data were recorded using six neuropixels probes inserted at the retinotopic center of six regions of the mouse visual cortex, while the mouse viewed a range of visual stimuli[2]. Of the 58 recording sessions included in the Allen Neuropixels Visual Coding dataset, we select nine sessions (corresponding to nine mice) from across the 'Brain Observatory 1.1' and 'Functional Connectivity' stimulus sets that met the following criteria. First, we select sessions containing channels in both the primary visual area (V1) and the lateral visual area (LM, which is reported to sit adjacent to V1 in the mouse visual hierarchy[2]). We then filter for sessions that LFP recordings from all available channels, and at least 20 identified units in both V1 and LM. Finally, we remove sessions with non-wildtype mice, as well as three sessions that could not be downloaded or lack LFP data during the flashes stimulus. We infer cortical depths as the dorsal–ventral coordinate of each channel relative to the coordinate of the first channel above the cortical surface. From each probe, we discard channels within 200 μm of the cortical surface, leaving LFP data from approximately 25 cortical channels in each of V1 and LM (spaced by 40 μm), with a sampling frequency of 1.25 kHz. To demonstrate that the dynamics of real neural activity exhibit similar characteristics to our model, we perform the same analyses on the spike and LFP recordings from the Neuropixels data as for our modelling results. Our study of the neuropixels dataset reveals that our model shares many phenomena with real experimental data, especially features such as intrinsically generated theta-gamma coupling, a low-dimensional communication subspace, and inter-areal phase locking, which previous models have struggled to mechanistically unify. In this section, we detail our procedure for analysing the neuropixels data and expand on the results introduced above. All data was accessed using the AllenSDK (`https://github.com/AllenInstitute/AllenSDK`) via the AllenNeuropixels.jl (`https://github.com/brendanjohnharris/AllenNeuropixels.jl`) package.

Partitioning evoked theta bursts

Having inspected the traces of LFP signals across channels and stimuli in V1 and LM, we noted that transient theta bursts occur during all high-contrast stimuli, including flashes and natural movies, between periods without clear narrow-band theta oscillations. We chose to study the flashes stimulus, since during this condition strong theta bursts are evoked by full-field flashes (which each last for 250ms, with 2s between flashes). To partition the narrow-band theta bursts from periods of low theta power, we extract all LFP traces from each trial of the flashes stimulus. We then calculate a theta score for each trace as the self-mutual information, at a lag of 50 samples (400 ms), of the first half of the signal. The theta score, which is high when a signal contains strong slow oscillations, allows us to select trials that evoke large theta bursts even when the signal has strong nonlinear characteristics. We choose a threshold of the average self-mutual information at 0.6 to select trials that exhibit strong evoked theta bursts, and from each selected trial we take the first half of the LFP signal (corresponding to 875 ms). After following this procedure to select trials of V1 LFP data, we then selected the same time points of LM LFP data. We then concatenate these theta burst LFP traces before calculating theta-partitioned power spectra (Supplementary Figure 8), comodulograms (Figure 4e), spike-LFP phase synchronization (Figure 4f), prediction performance (Figure 4g), and inter-areal phase locking (Supplementary Figure 9).

Experimental power spectra

We now present the power spectrum of the LFP time series for the theta-burst activity, as shown in Supplementary Figure 8. First, we apply a phase stitching algorithm to ensure that our calculated spectra are free of artefacts arising from the phase discontinuities present in our partitioned, concatenated, theta-burst time series. Briefly, our phase-stitching algorithm involves the following steps. For the LFP of each theta burst, we calculate the Hilbert phase and remove the first and last tenth of samples to account for edge effects. We then concatenate successive burst time series by finding the first sample in each time series that has the same phase (within a tolerance of 0.05 radians) as the final sample from the previous time series. After applying this phase-stitching algorithm, we find that the spectrum for burst periods exhibits a theta peak at approximately 4 Hz as well as a narrow-band gamma peak at $\sim$ 55Hz (see Supplementary Figure 8), both of which sit above a $1/f$ aperiodic noise component, in close agreement with the power spectra of multi-unit activity in our computational model (Figure 1e). Code implementing the phase-stitching algorithm is available in TimeseriesTools.jl (`https://github.com/brendanjohnharris/TimeseriesTools.jl`), along with code for the Welch periodogram method used to estimate the spectral density.

LFP–LFP phase-locking index

To further demonstrate that communication between brain regions in real experimental data is enhanced during burst periods, we calculate the phase-locking index between LFP signals from V1 and LM in the Neuropixels data. Using a single representative mouse, we partition the longest interval of the flashes stimulus (see above), without phase matching the resulting time series. We then calculate the LFP-LFP phase-locking index (PLI) between all pairs of channels in V1 (19 channels) and LM (18 channels), across the 54 detected trials with strong theta bursts. To compare with the phase-locking index during non-burst periods, we select 54 windows of the LFP during the spontaneous stimulus with an equal length as the trials of the flashes stimulus, and perform the same calculation. We calculate the PLI using the pairwise-phase consistency[3] after applying band-pass filters with a width of 15 Hz and center frequencies of $30, 32, \ldots, 250$ Hz. As shown in Supplementary Figure 9a, we find that the phase locking between LFP in LM and deep layers of V1 is significantly greater during the burst periods (up to $\times 4$), particularly around the 50Hz narrow-band gamma peak ($p < 10^{-100}$ between $40 - 60$Hz, corrected with the Benjamini-Hochberg method). To illustrate this effect, we show the phase-locking index across frequencies in the deepest channels of V1 in Supplementary Figure 9b.

Supplementary Methods 3: An alternative method to track the wave patterns

In addition to tracking the wave pattern using the center of mass (CoM) of the global firing rate activity of excitatory neurons, another approach involves detecting the pattern by fitting a 2D Gaussian function to the instantaneous firing rate profile of the excitatory neurons[4]. We model the firing activity of excitatory neuron j at location $\mathbf{y}_j$ is a Poisson process with its rate r_j equal to

$$r_j(t) = b_0(t) + a_0(t)e^{(-\|\mathbf{y}_j - \mathbf{y}_c(t)\|^2/2/\sigma(t)^2)}, \tag{14}$$

where $\mathbf{y}_{\mathrm{c}}(t)$, $\sigma(t)$, and $a_0(t)$, are the center, width, and height of the Gaussian function, respectively, and $b_0(t)$ is the baseline rate. The log-likelihood of these parameters is given by

$$\ln L = \sum_{j=1}^{N_{\mathrm{e}}} [-r_j(t) + n_j \ln(r_j(t))], \tag{15}$$

where n_j is the number of spikes emitted by excitatory neuron j in a small time window $\Delta t = 10$ ms. We determine the optimal value of $\mathbf{y}_{\mathrm{c}}(t)$, $\sigma(t)$, $a_0(t)$, and $b_0(t)$ at each time point t using the maximal likelihood method. We find the estimate center of the Gaussian function $\mathbf{y}_{\mathrm{c}}(t)$ closely aligns with the CoM of the global activity (Supplementary Figure 16), as evidenced by a minimal average distance between $\mathbf{y}_{\mathrm{c}}(t)$ and CoM. Specifically, the average distances are 2.12 ± 0.03 and 1.06 ± 0.02 grid points (mean $\pm$ SEM) for area 1 and area 2, respectively, over a 10-second simulation. Consequently, our results remain robust regardless of the method employed for detecting wave patterns. Given the computational efficiency of computing the CoM compared to estimating $\mathbf{y}_{\mathrm{c}}(t)$, we prioritize the use of CoM in our main results.

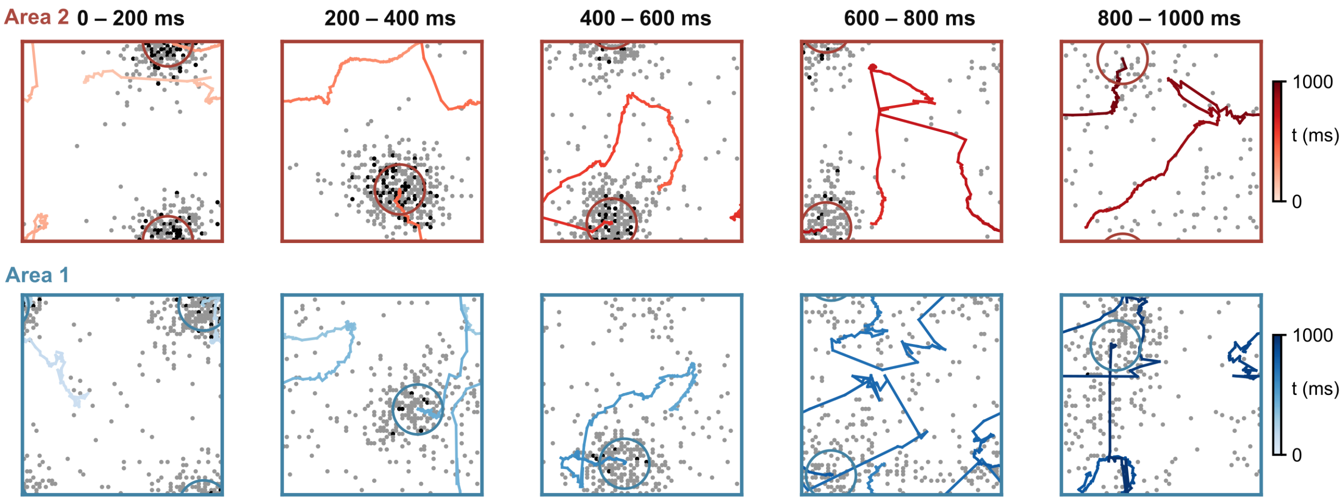

Supplementary Figure 1. Snapshots of wave pattern trajectories for spontaneous activities. Trajectories depict the center of mass movement of the wave patterns in area 1 (bottom, blue) and area 2 (top, red) over a 1000 ms interval. The spike counts of excitatory neurons during a 10 ms period are indicated by grey (1 spike) and black (2 spikes) dots.

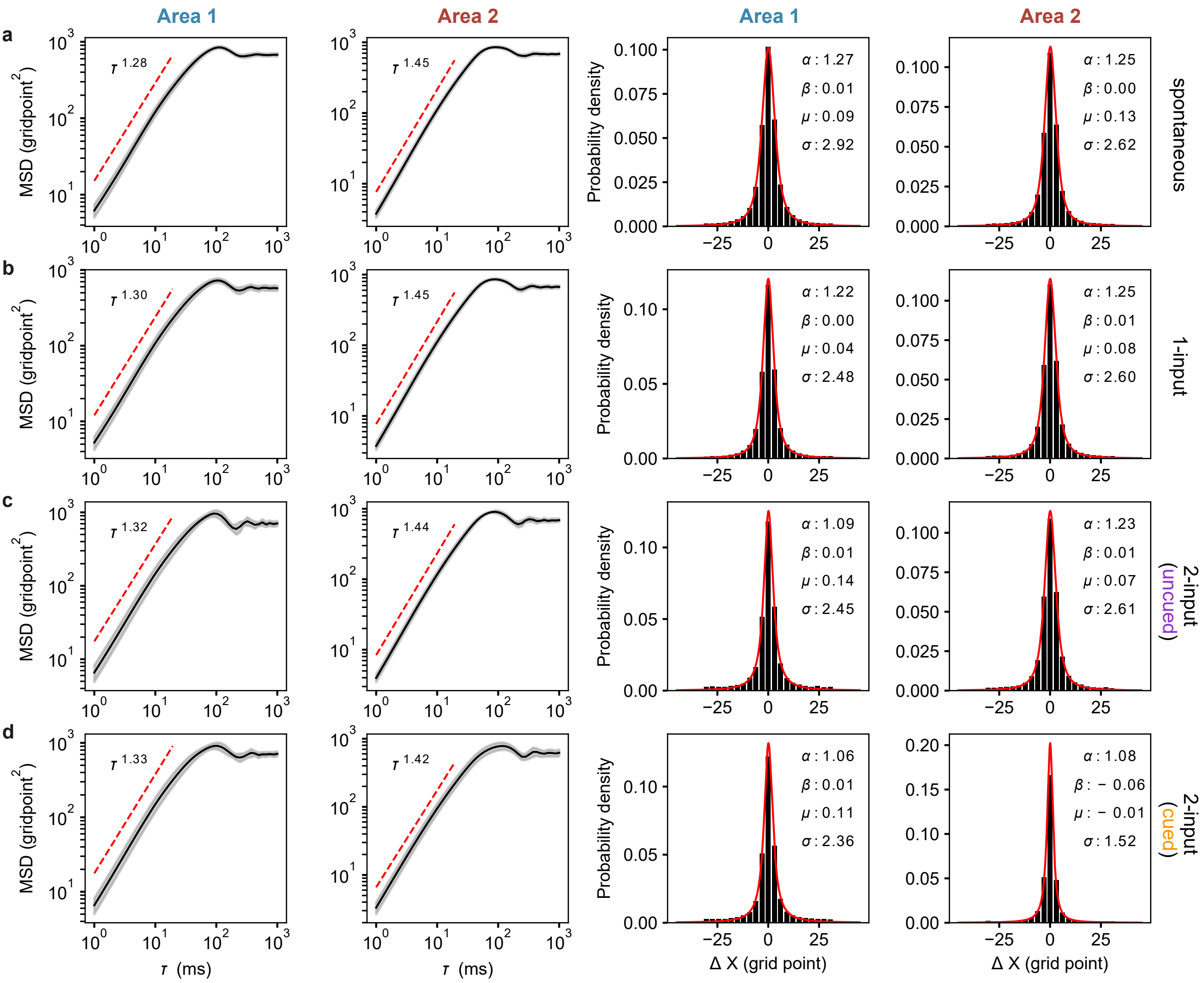

Supplementary Figure 2. Superdiffusive Lévy motion of localized spiking wave patterns. a First panel: mean squared displacement (MSD) of the pattern trajectory in area 1 during spontaneous activity. The dashed red line is a power law fit MSD$(t) \propto \tau^x$; $x > 1$ indicates superdiffusion. The shaded region represents ± 1 SD ($n = 60$ network realizations). Second panel: Same as the first panel but for area 2. Third panel: The distribution of the pattern displacement in area 1 over 10 ms on the vertical axis during spontaneous activity (59,940 data points from 60 networks). The red line is a fitted Lévy alpha-stable distribution, with best-fit parameters for the first shape parameter (α), second shape parameter (β), location (μ), and scale (σ) indicated. Fourth panel: Same as the third panel but for area 2. **b–d** Same as (**a**) but for the conditions of 1-input (**b**, $n = 60$ networks, 59,940 data points), 2-input (uncued) (**c**, $n = 30$ networks, 29,970 data points), and 2-input (cued) (**d**, $n = 30$ networks, 29,970 data points). For the 2-input (cued) condition, area 1 and area 2 represent the V4 and FEF, respectively.

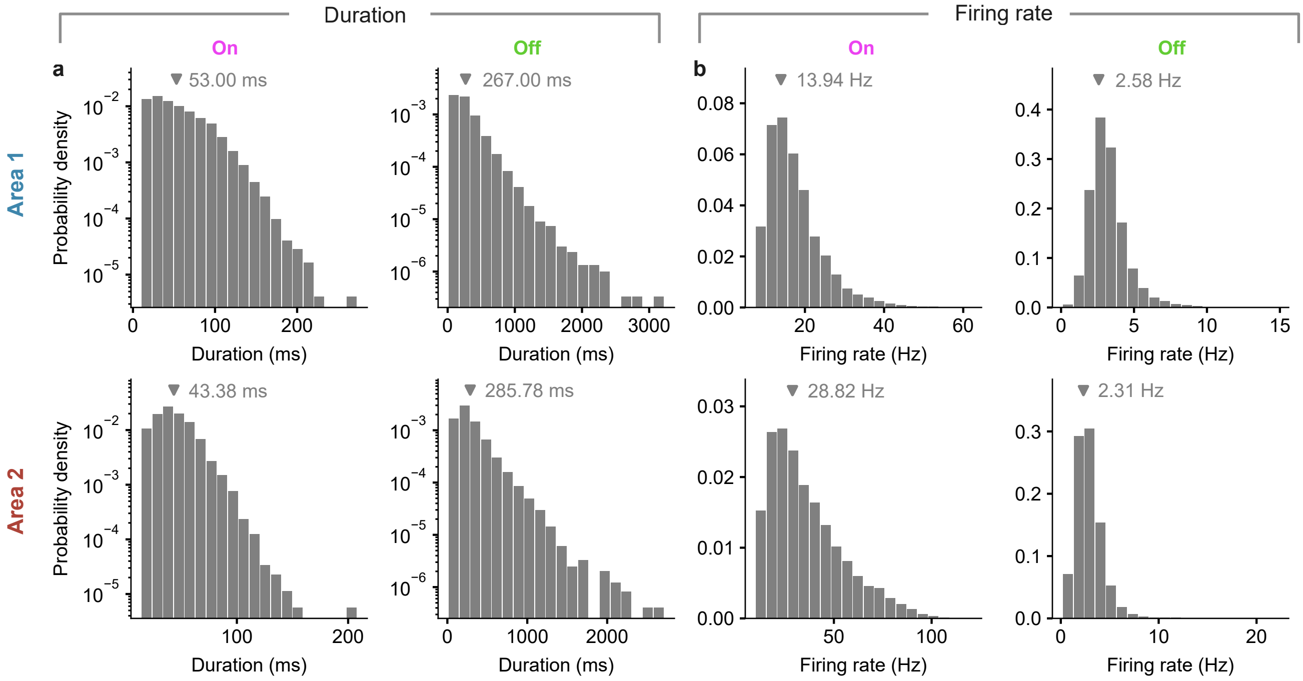

Supplementary Figure 3. Distributions of the duration and firing rate of On and Off states during spontaneous activity. a Distribution of the duration of On (left column) and Off (right column) states in area 1 (top row) and area 2 (bottom row) during spontaneous activity. The mean value of each distribution is indicated at the top (triangles). Data are from 30 random realizations of the network (200 seconds simulation for each realization; 6000 seconds in total). **b** Same as (**a**) but for the distribution of the firing rate.

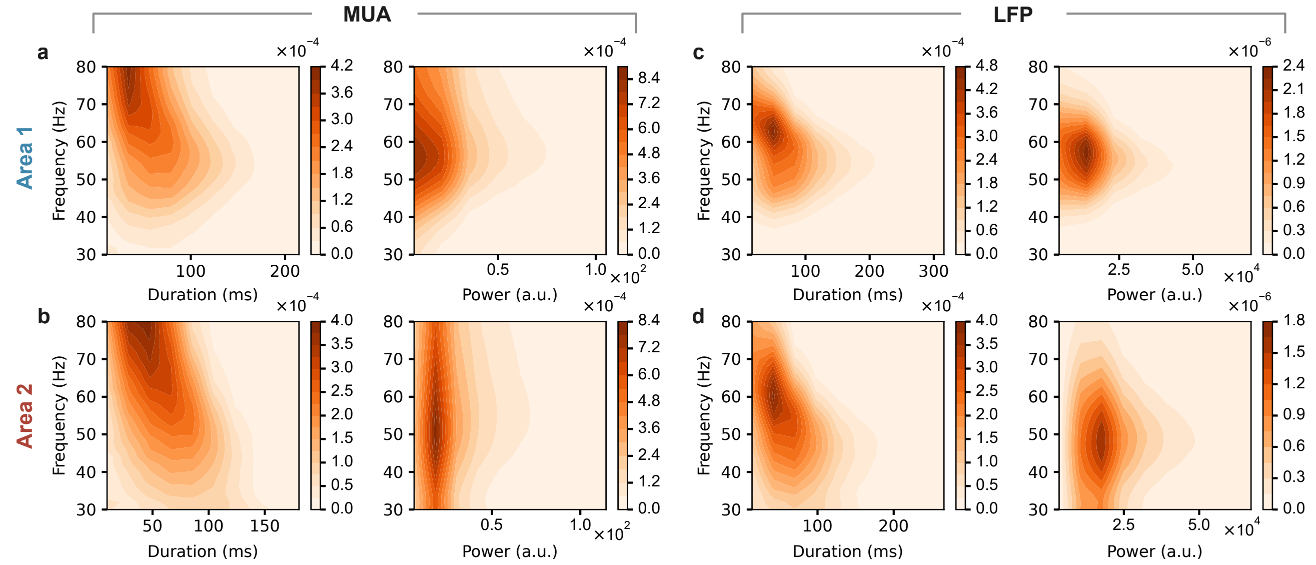

Supplementary Figure 4. Properties of gamma bursts. a Distributions of the duration (left panel) and power (right panel) of MUA gamma bursts in area 1. **b** Same as (**a**), but for area 2. **c** Distributions of the duration (left panel) and power (right panel) of LFP gamma bursts in area 1. **d** Same as (**c**) but for area 2.

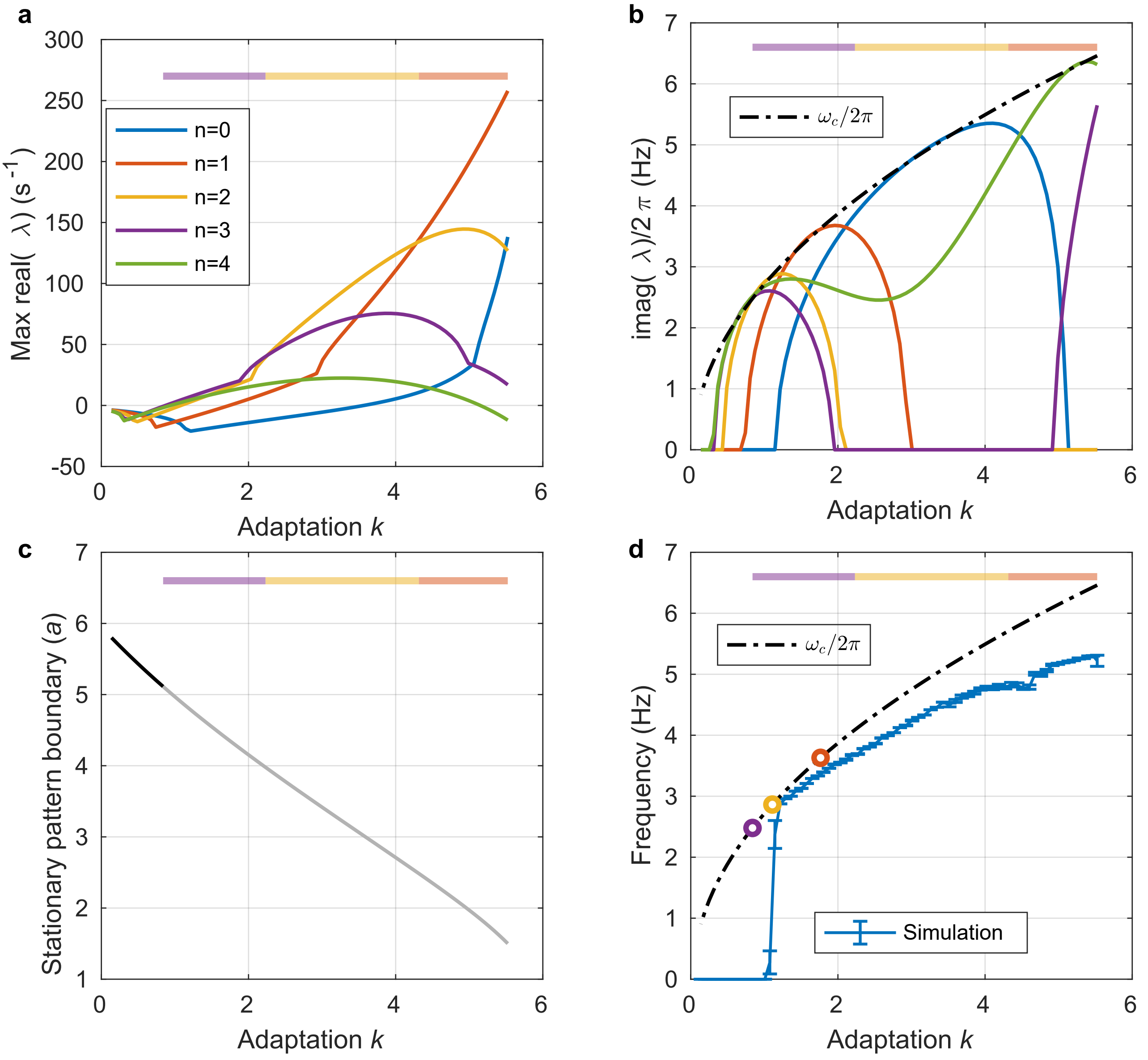

Supplementary Figure 5. Theoretical results about the effects of firing rate adaptation on the stability and oscillation properties of activity patterns in a neural field model. a Real part of eigenvalues [real(λ)] for each Fourier mode (shown in different colors) as a function of adaptation strength k. Only the larger real(λ) is shown if there are two real eigenvalues. The top horizontal line shows the region of k in which a particular mode has the largest positive real part of eigenvalues and thus dominates the oscillation (shown in the same color but with lower brightness as those for the eigenvalues; the same applies to the other panels in this figure). **b** Same as (**a**), but for the imaginary part of eigenvalues imag(λ). The black dashed line shows the theoretic critical Hopf frequency ω_c as a function of k. Note that the imag(λ) for each mode is tangent to ω_c at the bifurcation point. **c** Radius of the boundary (a) of the stationary localized activity pattern. The boundary is defined as the curve where the activity of neurons is equal to the activation threshold (see Supplementary Methods). The black line indicates that the stationary activity pattern is stable, while the grey line indicates instability. **d** Oscillation frequency of the activity pattern, measured as the frequency of the activity fluctuation at the pattern boundary, for different k in numerical simulations (blue line, error bars represent ± 1 SEM, $n = 20$ trials), compared to the critical Hopf frequency ω_c for three dominant modes (color dots on the black dash line).

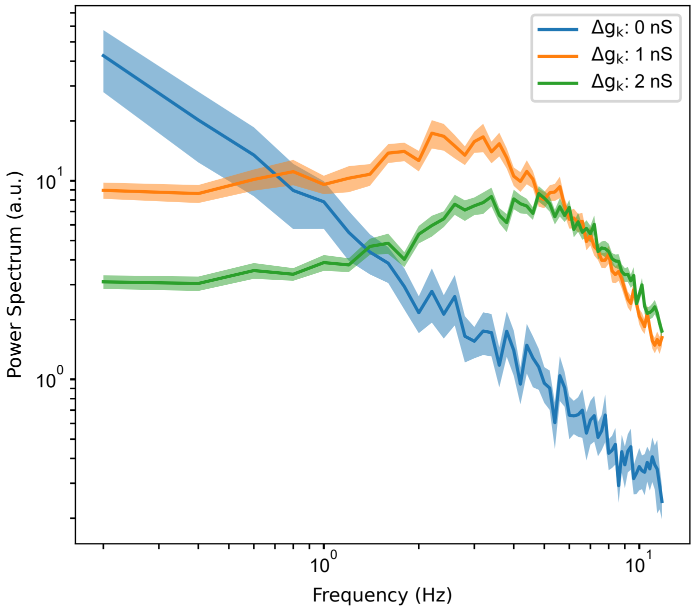

Supplementary Figure 6. Oscillation frequency of neural population activity in the spiking model increases with spike frequency adaptation. Power spectrum of the MUA in the center of area 1 for the adaptation strengths $\Delta g_{\mathrm{k}} = 0$ nS (blue), 1 nS (orange), and 2 nS (green). One input is added to the center of area 1 (input strength $c = 0.25$). Area 1 and area 2 are disconnected. Shaded regions indicate ± 1 SEM ($n = 200$ network realizations; power spectrum is calculated on 5-second MUA data for each network).

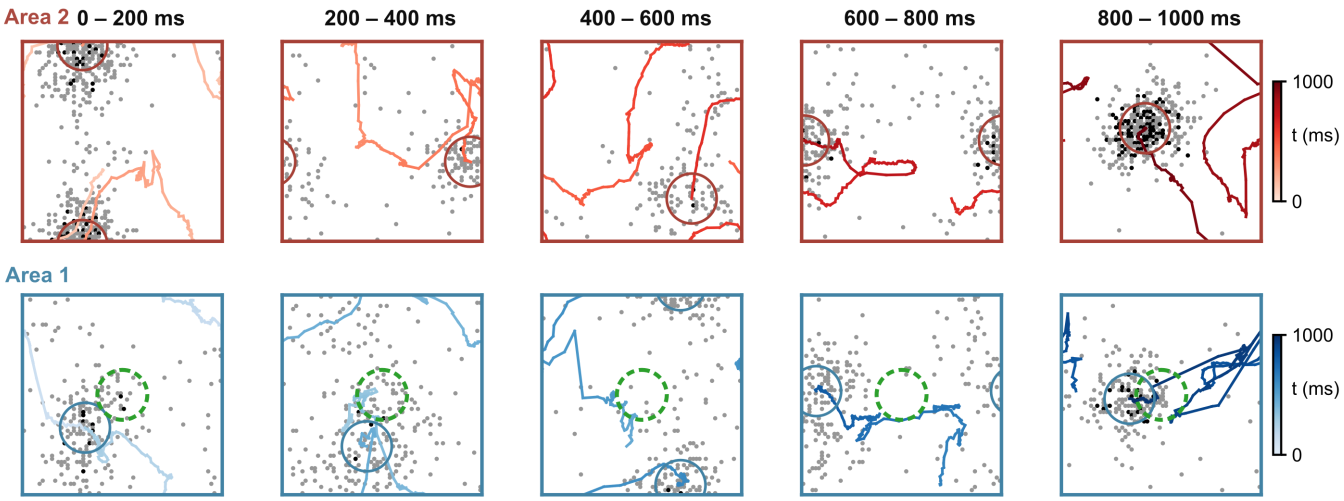

Supplementary Figure 7. Snapshots of wave pattern trajectories for the 1-input condition. Trajectories show the center of mass movement of the wave patterns in area 1 (bottom, blue) and area 2 (top, red) over a 1000 ms interval. The dashed green circle at the area 1 indicates the location of an external input. The spike counts of excitatory neurons during a 10 ms period are denoted by grey (1 spike) and black (2 spikes) dots.

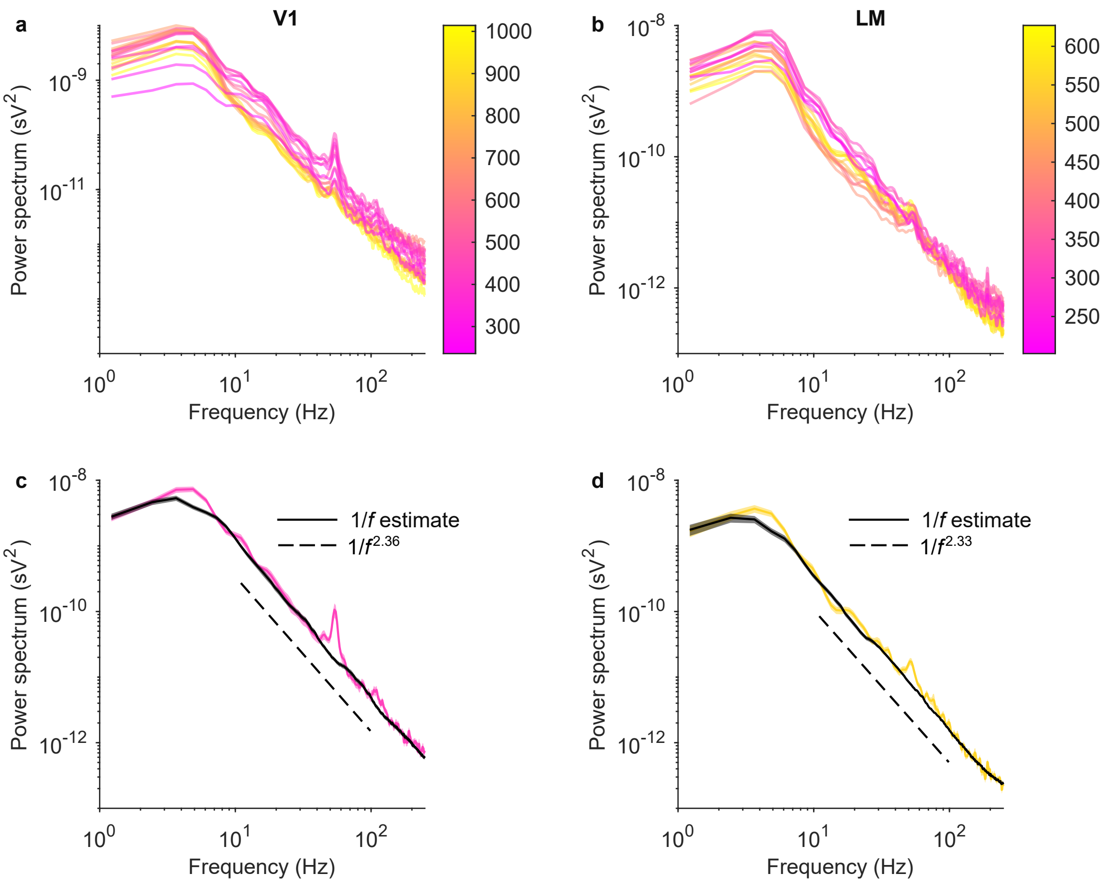

Supplementary Figure 8. Power spectrum of LFP data from a representative subject during flash stimuli for V1 and LM. a–b Power spectrum of phase-stitched burst LFP exhibit a theta peak at $\sim$ 4Hz and a gamma peak at $\sim$ 55Hz that sit above a $1/f$ arrhythmic component. Power spectrum are shown across channels, with channel depth indicated by the line color, for V1 (**a**) and LM (**b**). The narrow-band gamma peak is weaker in LM than V1, but LM also exhibits a high-band gamma peak at $\sim$ 195Hz. **c** The power spectrum of one example channel in V1 (**a**) and its separated arrhythmic $1/f$ component (black line). The exponent of the $1/f$ component is indicated by the dashed line. **d** Same as (**c**) but for one example channel in LM (**b**). For **a–d**, 24 seconds of phase-stitched LFP data are divided into 30 segments (800 ms each), and the power spectrum and its $1/f$ component is calculated for each segment and then averaged. Shaded regions in **c–d** indicate $\pm$1 SEM.

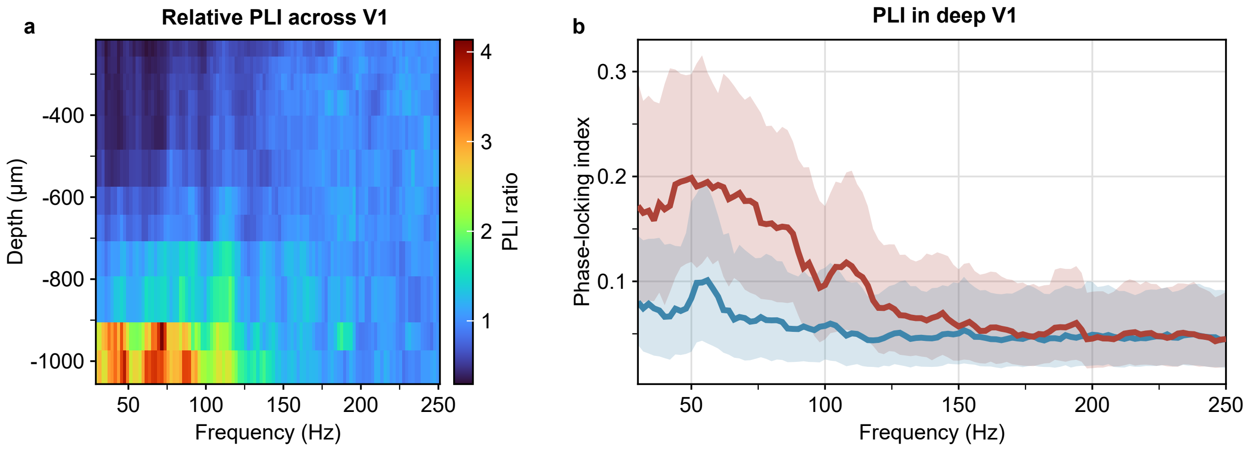

Supplementary Figure 9. LFP–LFP phase locking is enhanced during burst periods. a We calculate the LFP–LFP phase-locking index between V1 and LM LFP traces for all pairs of channels in a single representative subject. The ratio of the median phase-locking index (averaged over trials) during burst periods (flash stimuli) and non-burst periods (spontaneous stimuli) is shown across frequencies and channels of V1. Phase locking at $\sim$ 50Hz and $\sim$ 190Hz is both enhanced in deeper layers of V1. **b** The phase-locking index in deep layers of V1 (depth $< -900\,\mu$m) across frequencies. Solid lines depict the median phase-locking index over channels and windows, during burst periods (red) and non-burst periods (blue). Shaded bands indicate the interquartile range ($n = 19$ channels).

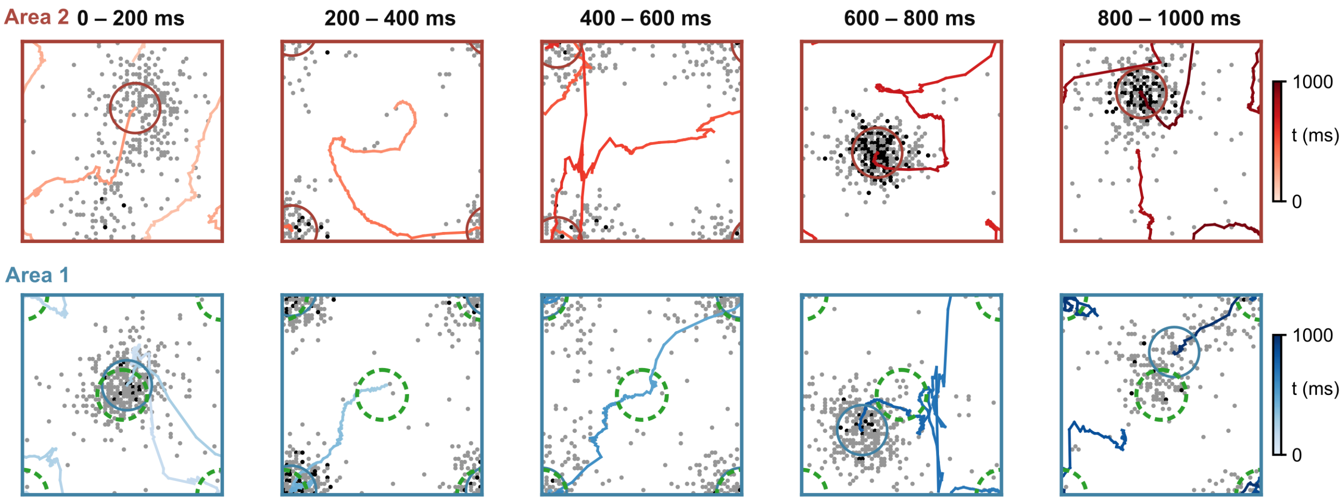

Supplementary Figure 10. Snapshots of wave pattern trajectories for the 2-input condition. Trajectories show the center of mass movement of the wave patterns in area 1 (bottom, blue) and area 2 (top, red) over a 1000 ms period. Two dashed green circles at the center and corner of area 1 indicate the locations of two external inputs. Due to periodic boundaries, the circle at the corner appears at all four corners of area 1. The spike counts of excitatory neurons during a 10 ms period are indicated by grey (1 spike) and black (2 spikes) dots.

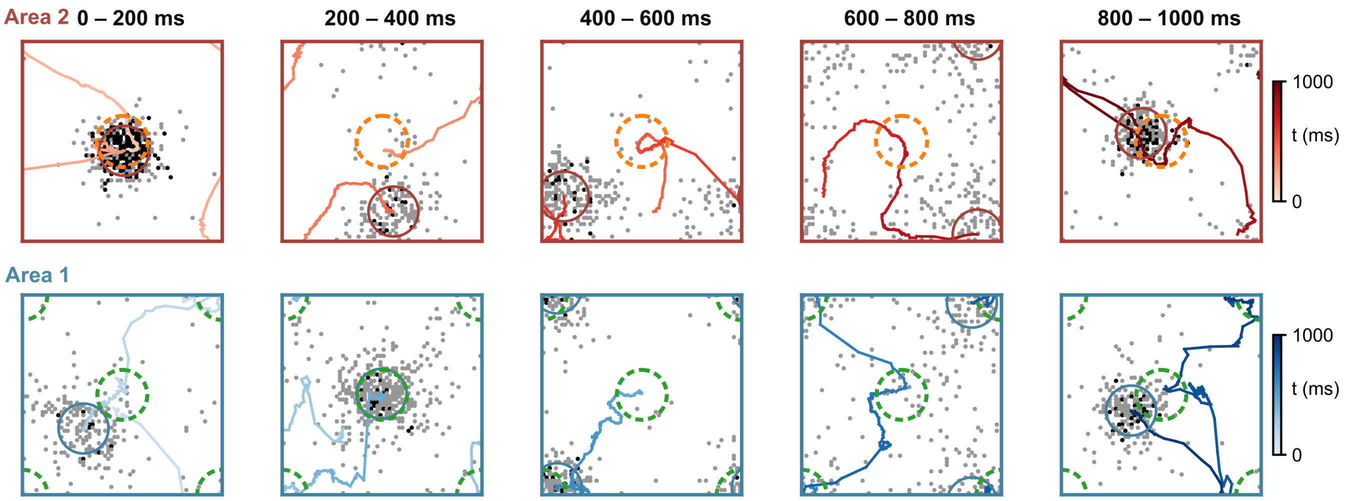

Supplementary Figure 11. Snapshots of wave pattern trajectories for the 2-input condition, with 1 input being cued. Trajectories depict the center of mass movement of the wave patterns in area 1 (V4, bottom blue) and area 2 (FEF, top red) over a 1000 ms period. Two dashed green circles at the center and corner of area 1 (V4) indicate the locations of two external inputs. The dashed orange circle at area 2 (FEF) indicates the cued location. The spike counts of excitatory neurons during a 10 ms period are indicated by grey (1 spike) and black (2 spikes) dots.

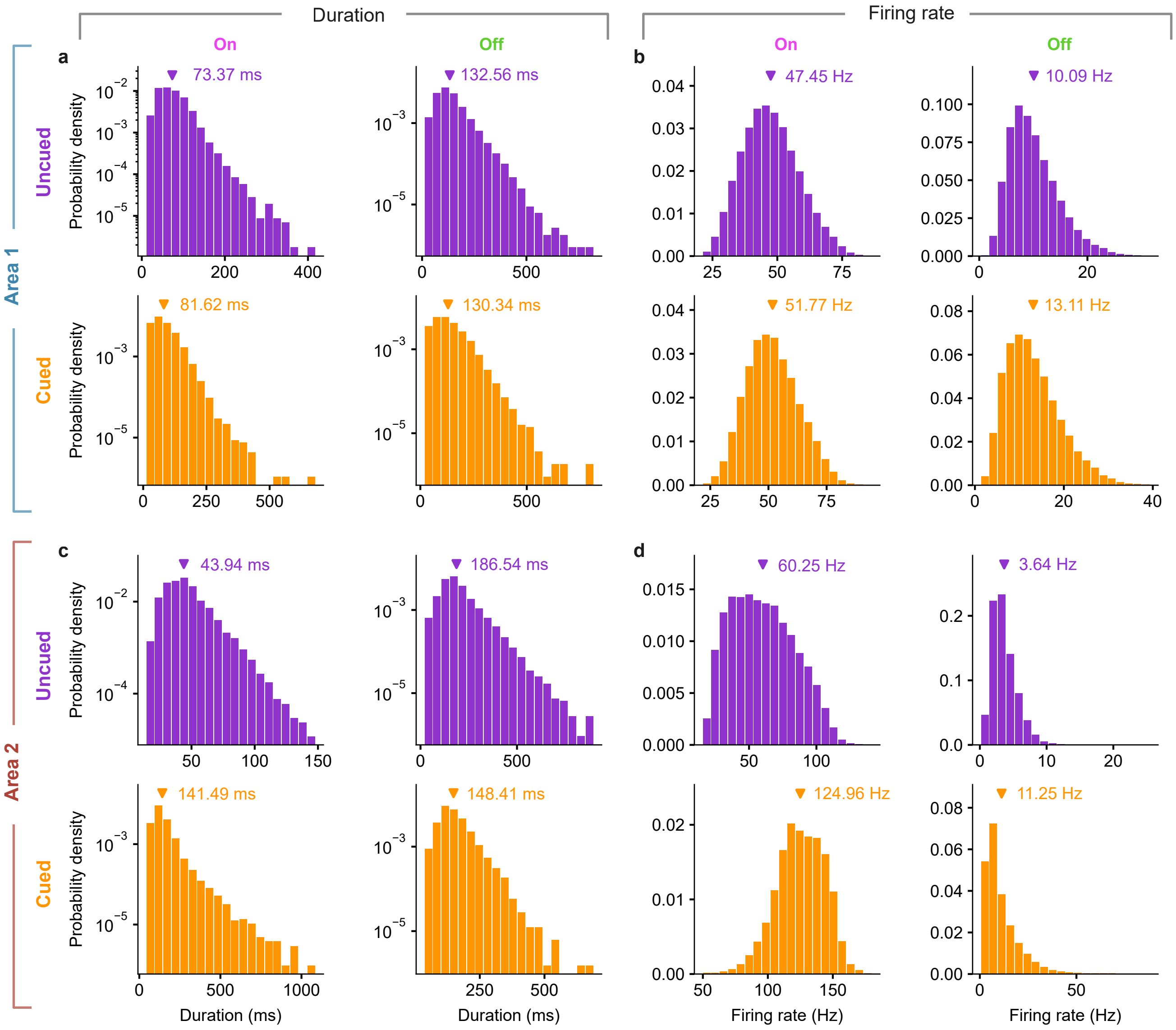

Supplementary Figure 12. Distributions of the duration and firing rate of On and Off states in uncued and cued conditions with two external inputs added. **a** Distribution of the duration of On (left column) and Off (right column) states at the center of area 1 (V4) when two inputs are placed at the center and corner of area 1, with the center input being uncued (top row) and cued (bottom row). The mean value of each distribution is indicated at the top (triangles). Data are from 30 random realizations of the network (196 seconds simulation for each realization; 5880 seconds in total) **b** Same as (**a**) but for the distribution of firing rate. **c**–**d** Same as (**a**–**b**) but for area 2 (FEF).

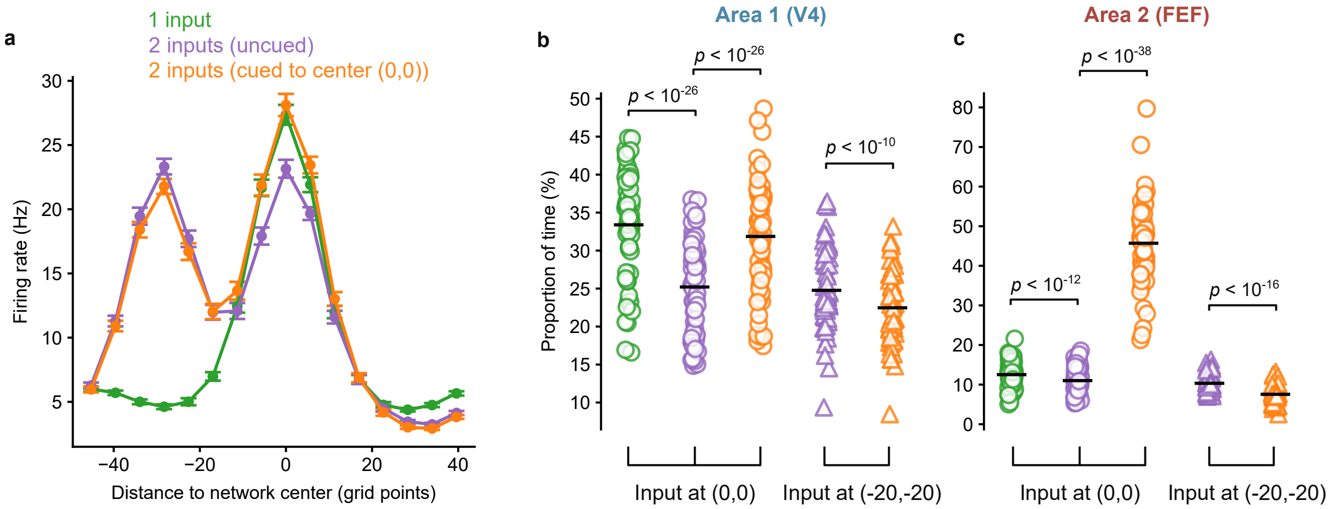

Supplementary Figure 13. Biased competition. a Neuronal response along the diagonal of area 1 circuit under different conditions. The response to a single input at coordinate (0, 0) (green) is suppressed when adding another input at a nearby position at (-20, -20) (purple). The top-down attention deployed to the (0, 0) position enhances the response to the attended input while suppresses the unattended one (orange). The data represents average firing rate across 60 random network realizations, with error bars denoting ±1 SEM. **b** The proportion of time that the wave pattern in area 1 (V4) samples each input (circles: input at (0,0); triangles: input at (-20,-20)) changes with the number of inputs and cue conditions. The pattern is regarded as sampling an input when its trajectory is less than 8 grid points from the input center. Each data point represents one random network realization ($n = 60$), with the black line indicating the mean. P-values are calculated using two-sided paired t-test. **c** Same as **b**, but for area 2 (FEF).

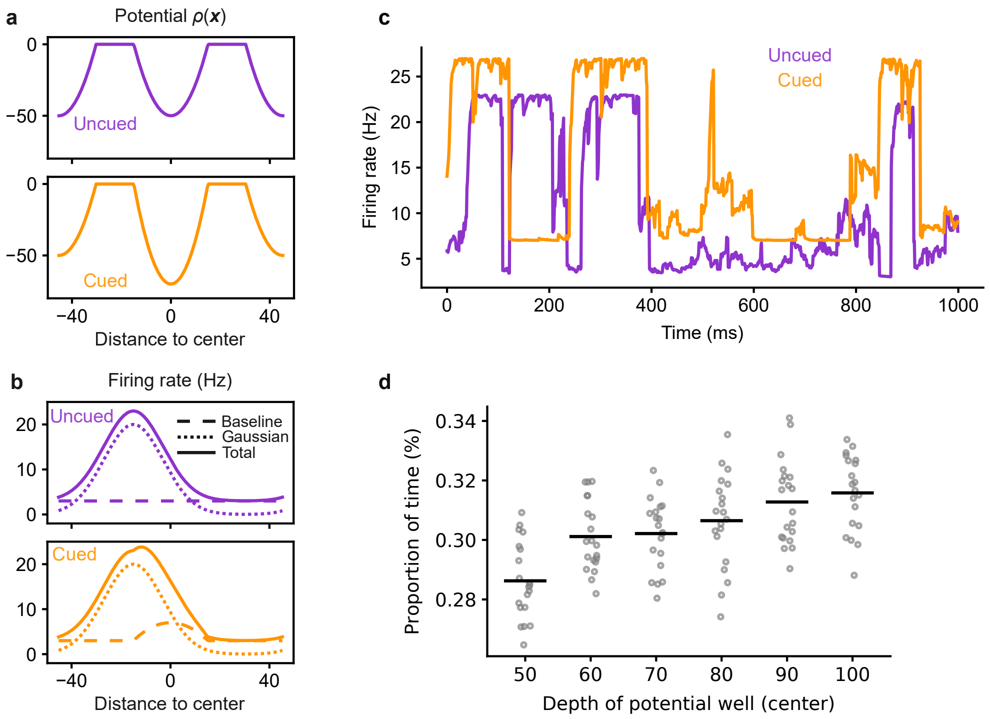

Supplementary Figure 14. Related to Figure 8 in the main text. Potential well and firing rate in the mathematical model. a Profile of the potential along the diagonal of the mathematical model in Figure 8. Under the uncued condition, two wells with equal depth are added at the center and corner to represent two inputs (purple). Under the cued condition, the depth of the center well is increased (e.g., to 70 in this example) to implement the top-down attention deployed to the center well/input (orange). **b** Baseline firing rate (long dashed line), a snapshot of the firing rate of the pattern with a Gaussian profile centered at the random walker located at the diagonal of the model (short dashed line), and the total firing rate (baseline + Gaussian, solid line) along the diagonal of the model in the uncued (purple) and cued (orange) conditions. Note that in the cued condition the baseline rate at the center potential well is increased proportionally to the increase in the center potential well depth. **c** Example traces of the instantaneous firing rate of a single neuron at the center of the model in the cued and uncued conditions illustrated in (**a**) and (**b**). **d** The proportion of time that the distance between the random walker and the center potential well is less than 10 grid points, serving as the probability that the random walker visits the center potential well/input. This probability is increased by the center potential well's depth (one-way ANOVA, $F_{5,114} = 12.48$, $p = 1.15 \times 10^{-9}$). Each data point represents the result obtained from one 200-second trial ($n = 20$); the horizontal line represents the mean across trials.

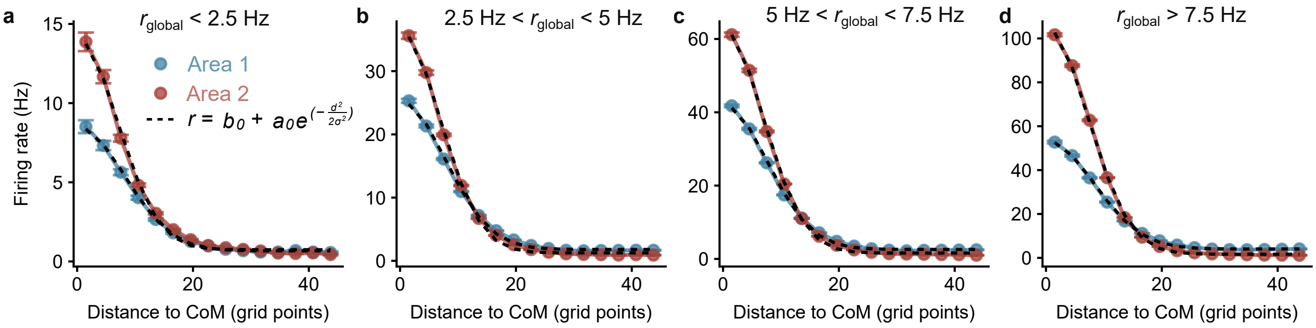

Supplementary Figure 15. Firing rate of neurons as a function of distance to the center of mass of wave patterns. a–d Average firing rate of excitatory neurons over 10 ms as a function of neurons' distance to the center of mass of wave patterns for area 1 (blue) and area 2 (red). The rate-distance relation is calculated for four ranges of global firing rate: $r_{\mathrm{global}} < 2.5$ Hz (**a**), $2.5 < r_{\mathrm{global}} < 5$ Hz (**b**), $5 < r_{\mathrm{global}} < 7.5$ Hz (**c**), and $r_{\mathrm{global}} > 7.5$ Hz (**d**). The dashed black lines indicate a fitting of a Gaussian function plus a baseline rate b_0 to the data. The results are based on 10-second simulations of spontaneous activities. Data are represented as the average across time points. Error bars indicate ± 1 SEM.

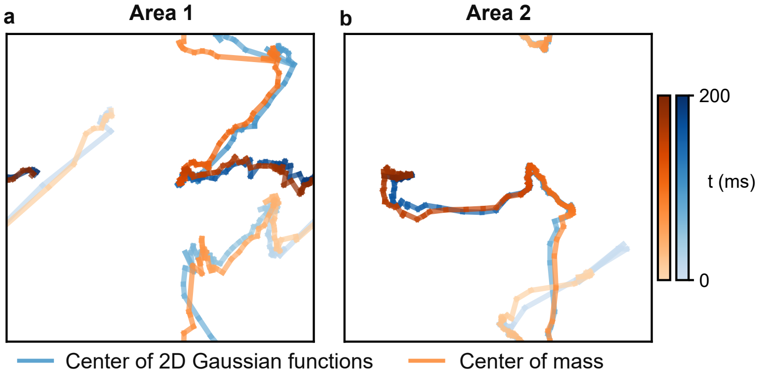

Supplementary Figure 16. Comparisons between different wave pattern detection methods. a–b The trajectories of the center of mass of global firing rate activities (orange) and the center of the 2D Gaussian functions fitted to the global firing rate profile (blue) in area 1 (**a**) and area 2 (**b**).

Supplementary References

1. Folias, S. E. & Bressloff, P. C. Breathers in two-dimensional neural media. *Phys. Rev. Lett.* **95**, 208107 (2005).

2. Siegle, J. H. *et al.* Survey of spiking in the mouse visual system reveals functional hierarchy. *Nature* **592**, 86–92 (2021).

3. Vinck, M., van Wingerden, M., Womelsdorf, T., Fries, P. & Pennartz, C. M. A. The pairwise phase consistency: a bias-free measure of rhythmic neuronal synchronization. *NeuroImage* **51**, 112–122 (2010).

4. Gu, Y., Qi, Y. & Gong, P. Rich-club connectivity, diverse population coupling, and dynamical activity patterns emerging from local cortical circuits. *PLoS Comput. Biol.* **15**, e1006902 (2019).
