# Supplementary material for: Distributed and dynamical communication: a mechanism for flexible cortico-cortical interactions and its functional roles in visual attention
Source: Commun Biol. 2024 May 8;7:550. doi: 10.1038/s42003-024-06228-z (PMC11078951; doi:10.1038/s42003-024-06228-z)
Supplement: Supplementary file 2 — Description of Additional Supplementary Files [file 42003_2024_6228_MOESM2_ESM.pdf]

# Description of Additional Supplementary Files

**File name:** Supplementary Data 1

**Description:** The source data underlying the graphs presented in the main figures.

**File name:** Supplementary Movie 1

**Description:** The movie that shows the spontaneous activities in our spiking neuron circuit model.

The spiking activities in areas 1 and 2 during spontaneous activity over a 1000 ms period are shown. No input or cue is included. The spike counts of excitatory neurons over a 10 ms period are represented by grey (1 spike) and black (2 spikes) dots. The red dot indicates the center of mass of the population's spiking activities.

**File name:** Supplementary Movie 2

**Description:** The movie that shows the activities in our spiking neuron circuit model during a simulated cued attention task.

This movie depicts the spiking activities in areas 1 (V4) and 2 (FEF) over a 1000 ms period. Two inputs are presented in area 1, at the center and corner respectively, with the center input being cued. The locations of these inputs are marked by green circles in area 1, and the cued location by an orange circle in area 2. The spike counts of excitatory neurons during a 10 ms period are indicated by grey (1 spike) and black (2 spikes) dots. The red dot shows the center of mass of the population's spiking activities.
